# Hfq and sRNA 179 Inhibit Expression of the Pseudomonas aeruginosa cAMP-Vfr and Type III Secretion Regulons

**DOI:** 10.1128/mBio.00363-20

**Published:** 2020-06-16

**Authors:** Kayley H. Janssen, Jodi M. Corley, Louise Djapgne, J. T. Cribbs, Deven Voelker, Zachary Slusher, Robert Nordell, Elizabeth E. Regulski, Barbara I. Kazmierczak, Emily Williams McMackin, Timothy L. Yahr

**Affiliations:** a Department of Microbiology and Immunology, University of Iowa, Iowa City, Iowa, USA; b Department of Medicine, Yale University School of Medicine, New Haven, Connecticut, USA; c Department of Microbial Pathogenesis, Yale University School of Medicine, New Haven, Connecticut, USA; University of Washington

**Keywords:** *Pseudomonas aeruginosa*, sRNA, Hfq, type III secretion, Vfr, ExsA, RsmA

## Abstract

Pseudomonas aeruginosa is a Gram-negative opportunistic pathogen causing skin and soft tissue, respiratory, and bloodstream infections. The type III secretion system (T3SS) is one important virulence factor. Production of the T3SS is controlled by ExsA, a transcription factor that activates expression of the entire T3SS regulon. Global regulators including Vfr, RsmA, and Hfq also contribute to regulation of the T3SS. Vfr is a cAMP-responsive transcription factor that activates *exsA* transcription. RsmA, an RNA-binding protein, inversely controls expression of the T3SS and the type VI secretion system (T6SS). Hfq is an RNA chaperone that functions by stabilizing small noncoding RNAs (sRNAs) and/or facilitating base pairing between sRNAs and mRNA targets. A previous study identified sRNA 1061, which directly targets the *exsA* mRNA and likely inhibits ExsA synthesis. In this study, we screened an sRNA expression library and identified sRNA 179 as an Hfq-dependent inhibitor of T3SS gene expression. Further characterization revealed that sRNA 179 inhibits the synthesis of both ExsA and Vfr. The previous finding that RsmA stimulates ExsA and Vfr synthesis suggested that sRNA 179 impacts the Gac/Rsm system. Consistent with that idea, the inhibitory activity of sRNA 179 is suppressed in a mutant lacking *rsmY* and *rsmZ*, and sRNA 179 expression stimulates *rsmY* transcription. RsmY and RsmZ are small noncoding RNAs that sequester RsmA from target mRNAs. Our combined findings show that Hfq and sRNA 179 indirectly regulate ExsA and Vfr synthesis by reducing the available pool of RsmA, leading to reduced expression of the T3SS and cAMP-Vfr regulons.

## Abstract (precis)

Control of gene expression by small noncoding RNA (sRNA) is well documented but underappreciated. Deep sequencing of mRNA preparations from Pseudomonas aeruginosa suggests that >500 sRNAs are generated. Few of those sRNAs have defined roles in gene expression. To address that knowledge gap, we constructed an sRNA expression library and identified sRNA 179 as a regulator of the type III secretion system (T3SS) and the cAMP-Vfr regulons. The T3SS- and cAMP-Vfr-controlled genes are critical virulence factors. Increased understanding of the signals and regulatory mechanisms that control these important factors will enhance our understanding of disease progression and reveal potential approaches for therapeutic intervention.

## INTRODUCTION

**FIG 1:**
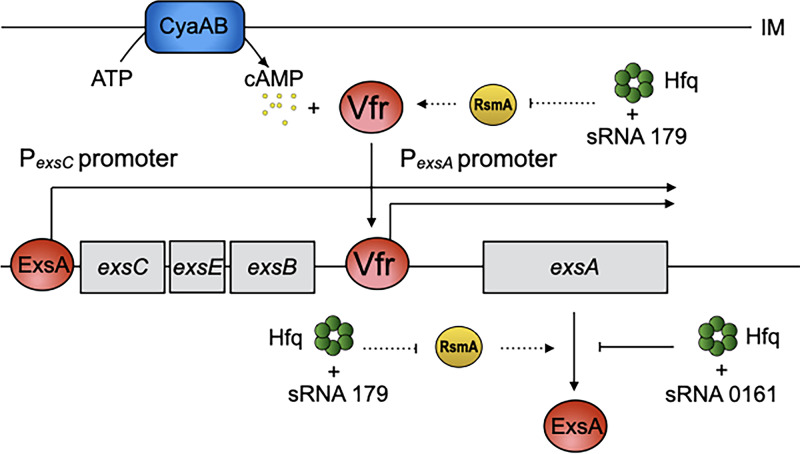
Model for control of T3SS gene expression by ExsA, Vfr, RsmA, and Hfq.

Pseudomonas aeruginosa is an important Gram-negative nosocomial pathogen that can cause skin and soft tissue, urinary tract, lung, and bloodstream infections (1). Many P. aeruginosa virulence factors are directly controlled by the cAMP-Vfr signaling (CVS) system (2, 3). The CVS consists of the CyaA and CyaB adenylate cyclases, a cAMP degrading phosphodiesterase, and the transcription factor Vfr (virulence factor regulator) (3). Vfr directly responds to increased intracellular cAMP pools to activate expression of the CVS regulon, which includes type IV pili, a type II secretion system, secreted factors (e.g., exotoxin A and protease IV), and the type III secretion system (T3SS) (4–11). The T3SS is an important virulence factor that contributes to the pathogenesis of human and animal infections (12–14). The T3SS is a needle-like apparatus used to translocate at least four effectors with antihost properties into eukaryotic target cells. The combined activities of the effector proteins promote phagocytic evasion and systemic spread of the microorganism (14). The T3SS consists of ∼40 genes that include structural components of the secretion and translocation machinery, the effectors and their cognate chaperones, and regulatory functions (15). ExsA is an AraC/XylS family protein that activates expression of the entire T3SS regulon (16). ExsA expression is highly regulated at both the transcriptional and posttranscriptional levels (17). ExsA autoregulates its own transcription through the P*_exsC_* promoter to generate a polycistronic mRNA encoding *exsC*, *exsE*, *exsB*, and *exsA* (Fig. 1) (16). A 297-bp intergenic region separates *exsB* and *exsA* and contains a Vfr-dependent promoter (P*_exsA_*) dedicated to *exsA* transcription (Fig. 1) (18). Several other factors contribute to *exsA* transcription, including PsrA, which stimulates transcription from the P*_exsC_* promoter, and MvaT/MvaU, VqsM, and Fis, which modulate P*_exsA_* promoter activity (19–21).

Once transcribed, translation of ExsA appears to be inefficient, possibly owing to an inhibitory structure in the mRNA that reduces the efficiency of ribosomal recruitment (22). At least two RNA-binding proteins promote T3SS gene expression by stimulating ExsA synthesis. The RNA helicase DeaD is required for ExsA synthesis *in vivo* and stimulates ExsA translation *in vitro* (22). DeaD likely functions by denaturing an inhibitory structure in the *exsA* mRNA leader region to enhance ribosomal recruitment (22). RsmA, a member of the Escherichia coli CsrA family of RNA-binding proteins, can have both positive and negative effects on gene expression through direct interactions with target mRNAs (23). RsmA has positive effects on the synthesis of Vfr and ExsA through mechanisms that remain to be defined (Fig. 1) (24). The activity of RsmA is controlled by RsmY and RsmZ, small noncoding RNAs (sRNAs) with multiple RsmA binding sites. RsmY and RsmZ function by sequestering RsmA from target mRNAs (25). Transcription of *rsmY* and *rsmZ* is controlled by the GacAS two-component system (23). Increased levels of RsmY and RsmZ lead to RsmA sequestration and reduced T3SS gene expression.

Host factor for bacteriophage Qβ RNA replication (Hfq) was identified in E. coli for its essential role in bacteriophage replication (26). Hfq is an RNA-binding protein that functions as an RNA chaperone to stabilize sRNAs and/or facilitate imperfect base pairing between sRNAs and mRNA targets (27). The actions of Hfq and sRNA can affect translation and/or mRNA stability and result in negative or positive effects on gene expression. Hfq regulates ∼5% of the P. aeruginosa genome, including genes involved in stress responses, metabolism, and virulence (28–30). A P. aeruginosa *hfq* mutant has reduced alginate production, lipopolysaccharide (LPS), and quorum sensing and increased type III secreted products (29, 31). The mechanism of T3SS control by Hfq is poorly understood (31). sRNA PA0161 was recently identified using the GRIL-seq method and was proposed to interact with the *exsA* mRNA (32). In this study, we report that Hfq works with sRNA 0161 to directly inhibit ExsA synthesis and with sRNA 179 to indirectly regulate both *exsA* transcription and ExsA synthesis.

## RESULTS

### T3SS gene expression is derepressed in the absence of Hfq.

Disruption of *hfq* in E. coli results in pleiotropic phenotypes resulting in delayed growth, decreased negative supercoiling, increased cell size, and sensitivity to UV light (33, 34). Previous studies found that P. aeruginosa *hfq* insertion and deletion mutants in strain PAO1 have a growth defect when cultured in LB medium (28, 35). In the current study, we constructed an Δ*hfq* deletion mutant (residues Δ2 to Δ82) in P. aeruginosa strain PA103 and observed a growth defect. In our subsequent studies with the PA103 Δ*hfq* strain, emergence of growth suppressor mutants was common. A recent study by Hill et al. also noted the emergence of growth suppressor mutants with the PAO1 *hfq* deletion strain (35), and sequencing of several of the suppressor mutants demonstrated involvement of multiple genetic loci. Although the nature of the PA103 Δ*hfq* suppressor mutants was not determined in the current study, each of the Δ*hfq* mutant phenotypes reported below could be complemented by expressing *hfq* in *trans* from a plasmid. For this reason, we believe that the growth phenotype of the suppressor mutants is unrelated to Hfq effects on expression of the Vfr and T3SS regulons as described below.

**FIG 2:**
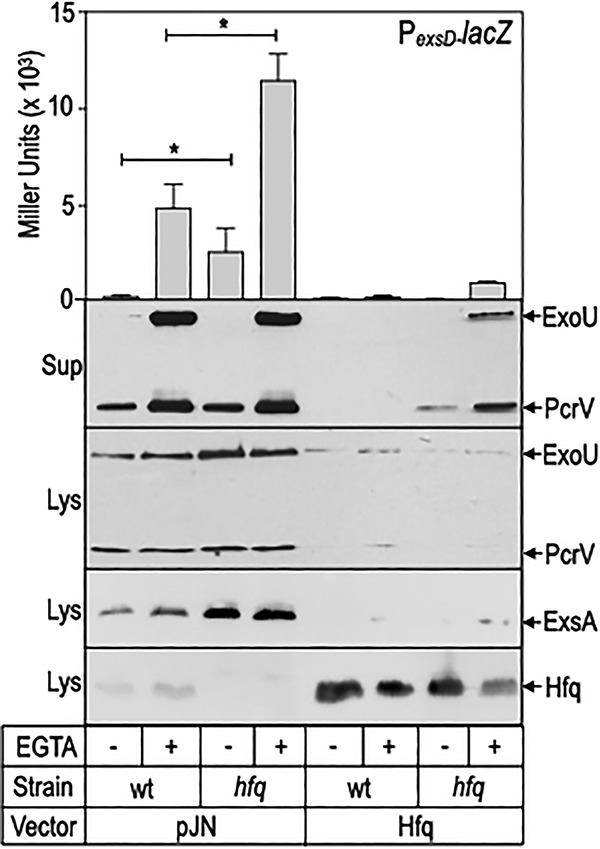
Hfq inhibits T3SS gene expression. Wild-type strain PA103 and an *hfq* mutant carrying an ExsA-dependent P*_exsD_-lacZ* transcriptional reporter were transformed with either a vector control (pJN105) or an Hfq expression vector (pHfq). Strains were cultured under noninducing (- EGTA) or inducing (+ EGTA) conditions for T3SS gene expression and assayed for P*_exsD_-lacZ* reporter activity. Beta-galactosidase activity is reported in Miller units with the standard error and represents the average of at least three independent experiments. *P* < 0.01. Cell lysate (Lys) or culture supernatant (Sup) fractions from the same cultures were immunoblotted for ExoU, PcrV, ExsA, and Hfq.

Previous studies found that Hfq contributes to P. aeruginosa T3SS gene expression (29, 31), but a regulatory mechanism was not described. To examine the contribution of Hfq to T3SS gene expression, a P*_exsD_*-*lacZ* transcriptional reporter was integrated into the ΦCTX phage attachment site of wild-type (WT) strain PA103 and the Δ*hfq* mutant. The P*_exsD_*-*lacZ* reporter is a measure of ExsA-dependent transcription and demonstrates strong induction when WT cells are cultured under inducing conditions for T3SS gene expression (low Ca^2+^, presence of EGTA) (Fig. 2) (36). P*_exsD_*-*lacZ* reporter activity increased significantly in the Δ*hfq* mutant under both noninducing (high Ca^2+^, no EGTA) and inducing conditions (Fig. 2). Corresponding immunoblots showed an increase in ExsA protein levels under both noninducing and inducing conditions in the Δ*hfq* mutant compared to the wild type. Whereas T3SS gene expression was derepressed in the *hfq* mutant, expression of Hfq from a plasmid inhibited P*_exsD_*-*lacZ* reporter activity as well as ExoU and ExsA production in both the WT and *hfq* mutant backgrounds (Fig. 2). Hfq protein was detectable in the WT strain, absent in the *hfq* mutant, and significantly elevated in strains carrying the Hfq expression plasmid (Fig. 2). These findings demonstrate that Hfq has a negative effect on T3SS gene expression.

### Hfq regulates T3SS-mediated cytotoxicity.

**FIG 3:**
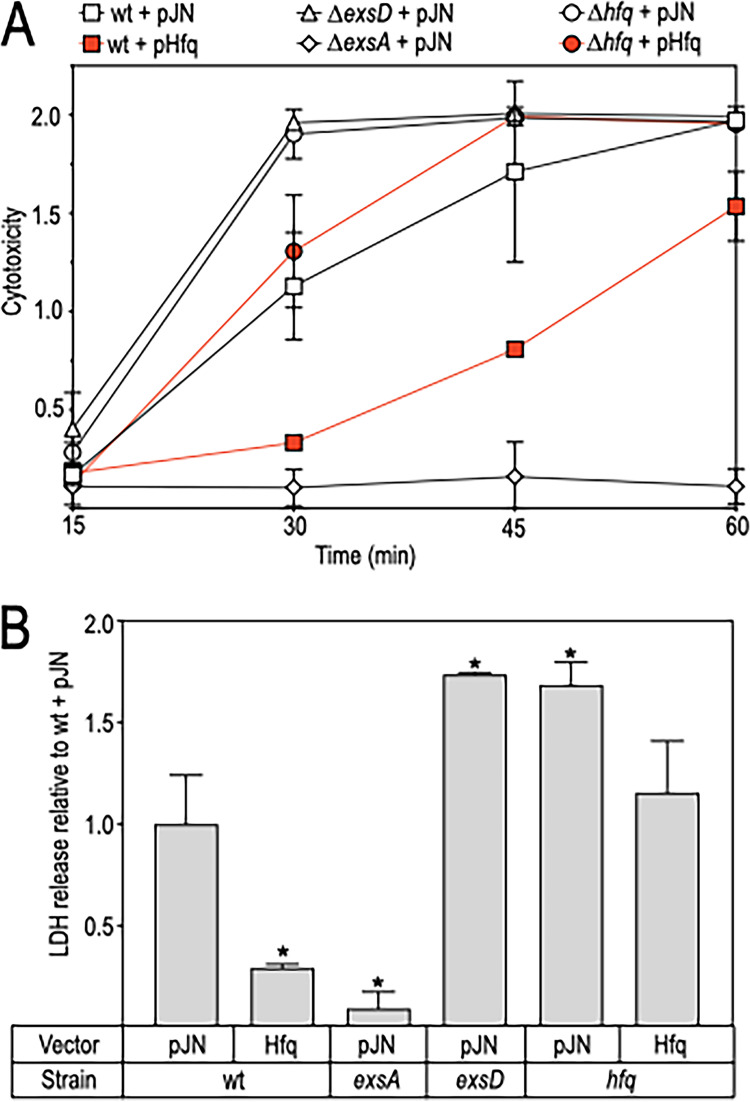
Hfq inhibits the T3SS-dependent toxicity of P. aeruginosa toward cultured cells. Chinese hamster ovary (CHO) cells were cocultured at an MOI of 10 with wild-type strain PA103, Δ*exsA*, Δ*exsD*, and an *hfq* mutant transformed with either a vector control (pJN) or an Hfq expression plasmid (pHfq). (A) Lactate dehydrogenase release as a marker of cytotoxicity was measured over a time course (reported in arbitrary units). The reported values represent the average of three wells with the standard error. (B) Lactate dehydrogenase release data from the 30-min time point in panel A relative to the wild-type PA103 vector control. *, *P* < 0.0001.

To verify that Hfq negatively regulates T3SS gene expression, we infected Chinese hamster ovary (CHO) cells with strains (wild type and *Δhfq*) carrying either a vector control or the Hfq expression vector. Previous studies have shown that coculturing CHO cells with P. aeruginosa strain PA103 results in acute cytotoxicity that is T3SS-dependent (37). In addition, the kinetics are advanced in strains derepressed for T3SS gene expression (e.g., an *exsD* mutant) (36). CHO cell lysis was measured by lactate dehydrogenase release following 15, 30, 45, and 60 min of coculture with P. aeruginosa (Fig. 3A). Relative to the parental strain, cytotoxicity was reduced with the *exsA* mutant, encoding a positive regulator of T3SS gene expression (Fig. 3A and B). In the absence of either *exsD* or *hfq*, cytotoxicity displayed enhanced kinetics, consistent with depressed T3SS gene expression. Expression of Hfq in the wild-type background resulted in an ∼4-fold reduction in cytotoxicity following 30 min of coculture relative to the vector control (Fig. 3A and B). Similarly, Hfq expression in the Δ*hfq* deletion strain also reduced cytotoxicity compared to the vector control, but not to the same levels observed in the wild-type background (Fig. 3B). These results suggest that Hfq negatively regulates T3SS-mediated cytotoxicity.

### Identification of sRNAs that regulate type III secretion.

**FIG 4:**
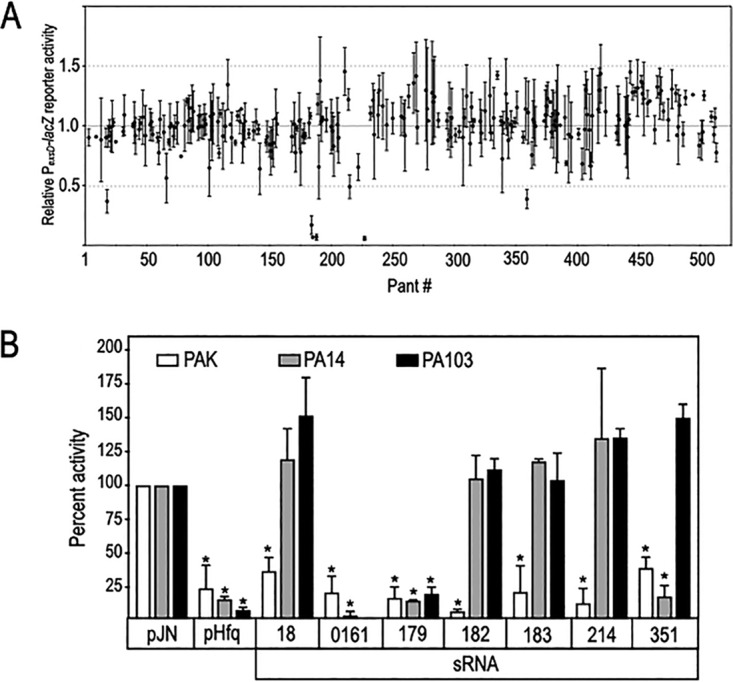
Identification of small noncoding RNAs that alter T3SS gene expression. (A) P. aeruginosa strain PAK carrying a P*_exsD_-lacZ* transcriptional reporter was transformed with a vector control and the sRNA expression vectors indicated in Table S1 (note that only 237 of the 512 putative sRNAs were tested). Strains were cultured under inducing (+ EGTA) conditions for T3SS gene expression in medium supplemented with 0.2% arabinose to induce sRNA expression and assayed for P*_exsD_-lacZ* reporter activity. The reporter values represent the activity of each sRNA relative to the vector control. (B) sRNAs that inhibited or stimulated P*_exsD_-lacZ* reporter activity greater than 2-fold (indicated by red lines in panel A) were selected for further analyses. Expression vectors for sRNA 18, 179, 182, 183, 214, 351, and 483 were introduced into strains PA14 and PA103 carrying the P*_exsD_-lacZ* reporter and assayed as described in panel A for reporter activity. sRNA 0161, previously shown to inhibit T3SS gene expression (32), and the Hfq expression vector were included as controls. The reported values represent the activity of each sRNA or pHfq relative to each parental strain carrying the vector control. The reported values with the standard error represent the average of at least three experiments. *, *P* < 0.01.

Hfq can inhibit protein synthesis by directly interacting with mRNA targets alone or by facilitating the base pairing of sRNAs with mRNA targets (27). To identify sRNAs that regulate the T3SS, we cloned and expressed a library of sRNAs in P. aeruginosa strain PAK and screened for effects on T3SS gene expression using the P*_exsD_*-*lacZ* transcriptional reporter. Gomez-Lozano et al. previously identified over 500 novel sRNAs in P. aeruginosa using three different methods to prepare transcript libraries (38). From this data set, we selected 240 sRNAs (referred to as Pants [P. aeruginosa novel transcripts]) in the original paper) that met two criteria, <200 nucleotides in length and known to originate from either the Watson or Crick strand of the genome (it has not been determined for all of the sRNAs). Each of the selected sRNAs was placed under the transcriptional control of an arabinose-inducible promoter. Six of the sRNAs (sRNA 18, 179, 182, 183, 214, and 351) inhibited P*_exsD_*-*lacZ* reporter activity greater than 2-fold in strain PAK (Fig. 4A and B).

10.1128/mBio.00363-20.3 TABLE S1 Strains and plasmids used in this study. Download Table S1, DOCX file, 0.03 MB. Copyright © 2020 Janssen et al. 2020 Janssen et al. This content is distributed under the terms of the Creative Commons Attribution 4.0 International license.

As a secondary screen, the six sRNAs identified using strain PAK were tested for activity in strains PA14 and PA103 bearing the P*_exsD_*-*lacZ* reporter (Fig. 4B). We also included sRNA PA0161 as a positive control. sRNA PA0161 (not included in our original library) was recently identified using GRIL-Seq as an inhibitor of T3SS gene expression that directly targets the *exsA* mRNA (32). Although sRNAs 18, 182, 183, and 214 possess regulatory activity in strain PAK, they had no effect on P*_exsD_*-*lacZ* reporter activity when expressed in strains PA14 and PA103 (Fig. 4B). sRNA 351 inhibited P*_exsD_*-*lacZ* reporter activity in both the PAK and PA14 backgrounds but was inactive in strain PA103. The Hfq expression vector inhibited P*_exsD_*-*lacZ* reporter activity in all three genetic backgrounds. Since only Hfq, sRNA 0161, and sRNA 179 inhibited T3SS gene expression in all three genetic backgrounds, the remainder of this study focused on those factors.

### sRNA 179 and Hfq function together to inhibit ExsA translation.

**FIG 5:**
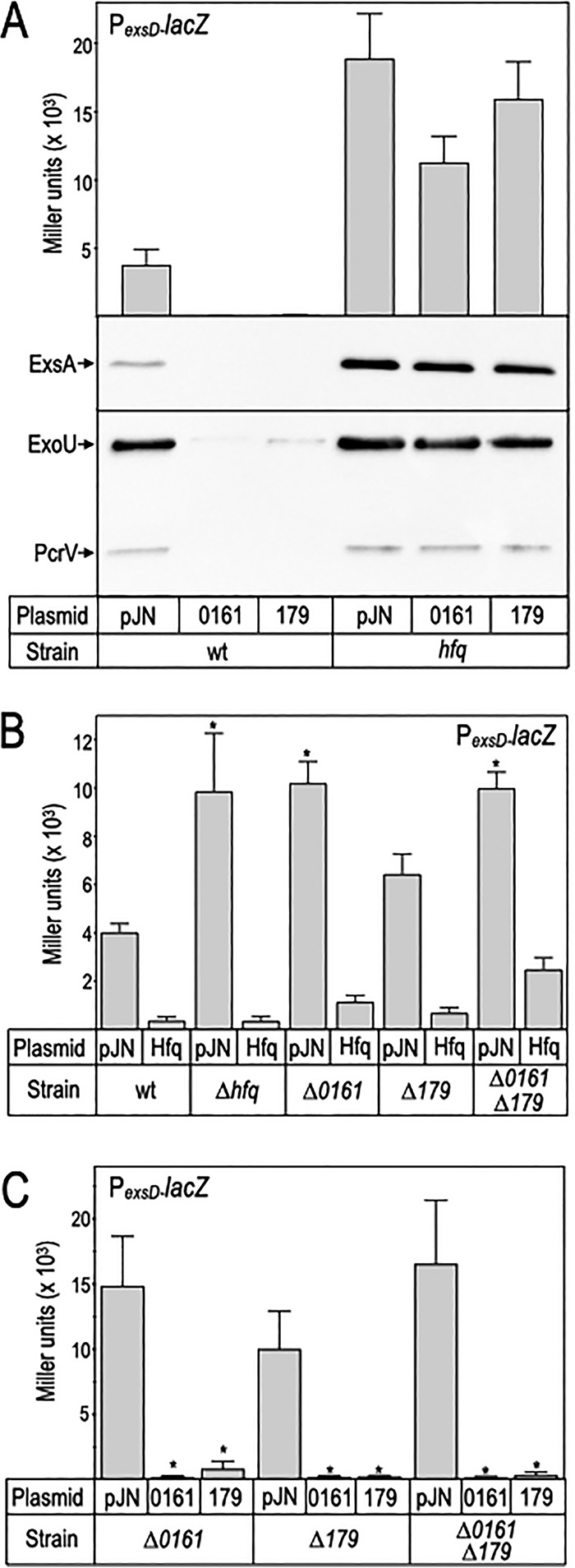
Inhibition of T3SS gene expression by sRNAs 0161 and 179 is Hfq-dependent. (A to C) The indicated strains carrying a P*_exsD_-lacZ* reporter were transformed with either the vector control (panels A to C), sRNA 0161, and 179 expression vectors (panel A and C) or an Hfq expression vector (panel B). Strains were cultured under inducing conditions for T3SS gene expression supplemented with 0.2% or 0.05% arabinose to induce sRNA or Hfq expression, respectively, and assayed for P*_exsD_-lacZ* reporter activity as reported in Miller units. Cell lysate or supernatant fractions from the samples in panel A were immunoblotted for ExsA or ExoU and PcrV, respectively. The reported values with the standard error represent the average of at least three experiments. *, *P* < 0.001 relative to the vector control in each case. (B) *, *P *< 0.005; (C) *, *P* < 0.05.

To determine whether the inhibitory activities of sRNAs 0161 and 179 are Hfq-dependent, both sRNAs were expressed in P. aeruginosa strain PA103 and the Δ*hfq* mutant. Whereas expression of either RNA in WT cells resulted in strong inhibition of P*_exsD_*-*lacZ* reporter activity, ExsA production, and ExoU secretion, the inhibitory activities of sRNAs 0161 and 179 were largely suppressed in the Δ*hfq* mutant (Fig. 5A). ExsA protein levels were reduced by ∼25% upon sRNA 0161 and 179 expression in the Δ*hfq* mutant relative to the vector control. In contrast, ExsA protein was reduced by >90% upon sRNA 0161 and 179 expression in the WT background. ExoU protein levels followed a similar trend. These data are consistent with sRNA 0161 and 179, both functioning as typical sRNAs that depend upon Hfq for facilitated base pairing with target mRNA(s).

The data presented thus far show that sRNAs 0161 and 179 expressed from a plasmid possess inhibitory activity. To examine the effects of sRNA 0161 and 179 expressed from their native chromosomal promoters, we constructed deletion mutants for each and a double mutant lacking both sRNA 0161 and 179. Deletion mutants lacking *hfq*, sRNA 0161, and both sRNAs (0161 and 179) carrying a vector control (pJN105) demonstrated significant depression of P*_exsD_*-*lacZ* reporter activity (Fig. 5B). Although reporter activity was consistently elevated in the single Δ179 mutant, that difference did not meet a statistical test of significance. To determine whether sRNA 0161 and 179 account for all of the Hfq-dependent activity, the Hfq expression plasmid was introduced into each of the sRNA mutant backgrounds. Hfq expression resulted in significant inhibition of P*_exsD_*-*lacZ* reporter activity in the single and double sRNA 0161 and 179 mutants (Fig. 5B). Finally, complementation analyses demonstrate that plasmid-expressed 0161 and 179 are sufficient to repress P*_exsD_*-*lacZ* reporter activity in their cognate mutant backgrounds and in the double 0161 and 179 mutant (Fig. 5C). These combined findings demonstrate that (i) Hfq functions together with sRNAs 0161 and 179 to inhibit T3SS gene expression, (ii) sRNA 0161 and 179 activities are independent of one another, and (iii) Hfq also inhibits T3SS gene expression in a manner that does not rely upon sRNA 0161 or 179. Such activity could reflect sRNA-independent activity or involvement of an additional sRNA(s).

### Hfq impacts ExsA synthesis at the posttranscriptional level.

**FIG 6:**
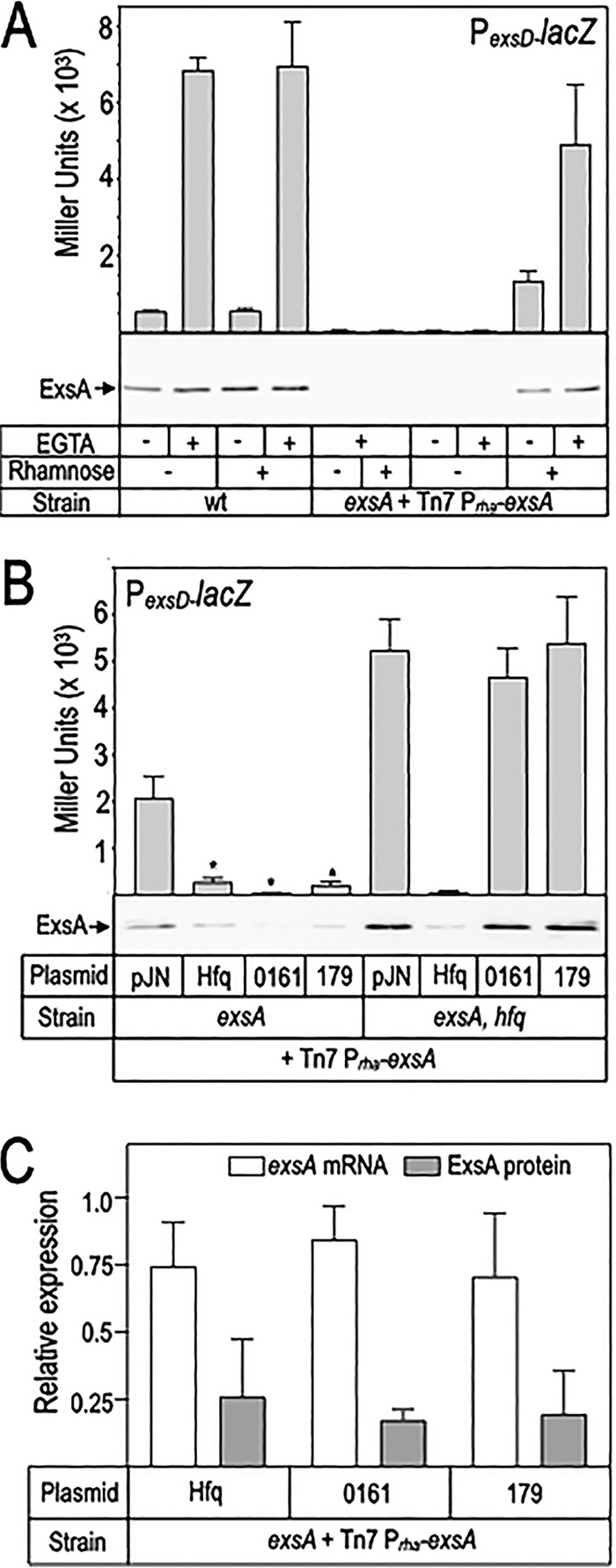
Hfq, sRNA 0161, and sRNA 179 inhibit ExsA synthesis. (A) WT PA103 and an *exsA* mutant carrying a rhamnose-inducible copy of *exsA* integrated at the Tn*7* site were cultured under noninducing (- EGTA) or inducing (+ EGTA) conditions for T3SS gene expression. Rhamnose (0.005%) was included in the growth medium to induce *exsA* expression from the Tn*7* integrant, and ExsA activity and protein levels were measured using the P*_exsD_-lacZ* reporter and ExsA immunoblots, respectively. (B) The indicated strains carrying the rhamnose-inducible copy of *exsA* were transformed with either the vector control, Hfq expression vector, sRNA 1061, or sRNA179 expression plasmids, cultured under T3SS inducing conditions, and assayed for P*_exsD_-lacZ* reporter activity and ExsA protein levels. (C) Comparison of *exsA* mRNA and ExsA protein levels in samples harvested from an *exsA* mutant carrying the rhamnose-inducible copy of *exsA* and either a vector control, pHfq, p0161, or p179. RNA samples were quantified for *exsA* using qRT-PCR and normalized to the *rimM* housekeeping gene. The reported values are relative to the *exsA* strain carrying the vector control.

Based on the previous observation that T3SS genes demonstrate increased mRNA levels in an *hfq* mutant (31), we hypothesized that Hfq, sRNA 0161, and/or sRNA 179, either directly or indirectly, regulate ExsA synthesis. Because ExsA autoregulates its own transcription, we established an experimental system to uncouple *exsA* transcription from ExsA synthesis. The native 101-nt upstream untranslated region (corresponding to the *exsA* transcription start site from the P*_exsA_* promoter) and coding sequence for *exsA* were placed under the transcriptional control of a rhamnose-inducible promoter (P*_rha_*) and integrated into the chromosome at the Tn*7* transposon insertion site of an Δ*exsA* strain. Titration of rhamnose identified conditions (0.005%) where P*_exsD_*-*lacZ* reporter activity and ExsA expression levels were similar to the WT PA103 strain (Fig. 6A; see Fig. S2 in the supplemental material). Expression of the ExsA-dependent P*_exsD_*-*lacZ* reporter was entirely dependent upon addition of rhamnose to the culture medium and remained responsive to calcium chelation by EGTA (Fig. 6A), thus validating the system.

10.1128/mBio.00363-20.1 FIG S1 (A) WT PA103 and an *exsA* mutant carrying a rhamnose-inducible copy of *exsA* integrated at the Tn*7* site were cultured under inducing conditions for T3SS gene expression. Rhamnose was included as indicated to induce *exsA* expression from the Tn*7* integrant (the concentrations used for the titration: 0.0025, 0.005, 0.01, 0.025, and 0.05%) ExsA activity was measured using the P*_exsD_-lacZ* reporter. Download FIG S1, TIF file, 0.3 MB. Copyright © 2020 Janssen et al. 2020 Janssen et al. This content is distributed under the terms of the Creative Commons Attribution 4.0 International license.

10.1128/mBio.00363-20.2 FIG S2 Hfq interacts with sRNA 0161 and the *exsA* mRNA leader region. (A and B) Electrophoretic mobility shift assays were performed by incubating radiolabeled sRNA 0161 (A) or *exsA* mRNA leader region (B) with the indicated concentrations of Hfq. Radiolabeled sRNA 0161 was also incubated with unlabeled *exsA* mRNA leader region to detect formation of an RNA:RNA hybrid. The positions of the respective complexes are indicated with arrowheads. Download FIG S2, TIF file, 0.3 MB. Copyright © 2020 Janssen et al. 2020 Janssen et al. This content is distributed under the terms of the Creative Commons Attribution 4.0 International license.

The Tn*7*-rhamnose system was then used to determine whether Hfq, sRNA 0161, and/or sRNA 179 effect ExsA synthesis at the posttranscriptional level. Expression of Hfq, sRNA 0161, and sRNA 179 each resulted in strong inhibition of P*_exsD_*-*lacZ* reporter activity and ExsA protein levels (Fig. 6B, lanes 1 to 4), and the activity of the sRNAs was entirely dependent upon Hfq (lanes 5 to 8). To verify that the reduction in ExsA synthesis was unrelated to changes in *exsA* mRNA levels, qRT-PCR for *exsA* was performed on RNA harvested from the samples shown in lanes 1 to 4. Whereas ExsA protein levels were reduced ∼4-fold in the Hfq, 0161, and 179 expression strains relative to the vector control, *exsA* mRNA levels were only slightly reduced (∼25%) (Fig. 6C). Some degradation of the *exsA* mRNA was expected owing to the lack of ribosomal protection that occurs during active translation. These findings are consistent with Hfq and sRNAs 0161 and 179 controlling ExsA synthesis at a posttranscriptional level.

### sRNA 0161 and the *exsA* mRNA interact with each other and Hfq.

Our finding that sRNA 0161 inhibits ExsA synthesis and the previous demonstration of a specific interaction between sRNA 0161 and the *exsA* mRNA by GRIL-seq (32) (Fig. 6B) was suggestive of a direct effect on ExsA translation. To measure the interaction *in vitro*, sRNA 0161 was *in vitro* synthesized, radiolabeled at the 5′ end, and incubated with an unlabeled portion of the *exsA* mRNA leader region prior to electrophoresis on a nondenaturing gel. The *exsA* probe (*exsA* 101) consists of the native 101 untranslated region and 20 bp downstream of the start codon. An sRNA 0161-*exsA* complex was observed when 20 nM unlabeled *exsA* mRNA leader region was added to the reaction (Fig. S2). We were unable to detect an interaction between sRNA 179 and the *exsA* mRNA using the same approach. We also assayed for Hfq binding and found that Hfq interacts with sRNA 0161 and/or the *exsA* mRNA (Fig. S3). These data are consistent with the previous GRIL-Seq findings of Zhang et al. (32) and suggest that Hfq directly interacts with sRNA 0161 and the 5′ untranscribed region (UTR) of *exsA* to reduce translation. Furthermore, the inability to detect an interaction between sRNA 179 and the *exsA* leader region suggested that the mechanism of inhibition by sRNA 179 may be indirect and distinct from the action of sRNA 0161.

### Hfq inhibits the Vfr-cAMP signaling system.

**FIG 7:**
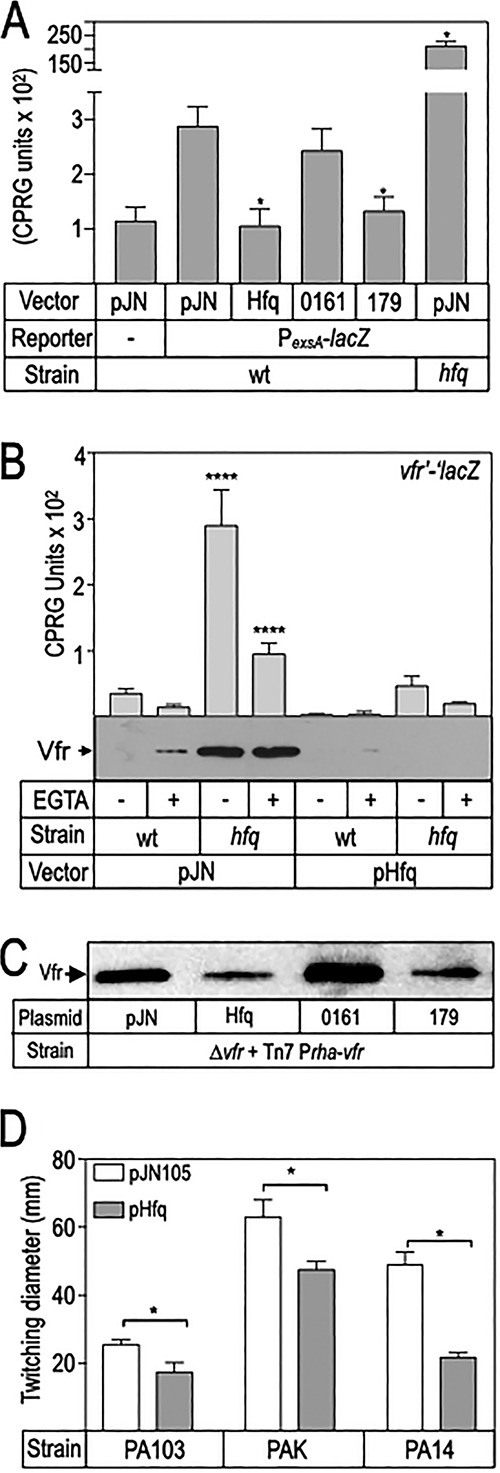
Hfq and sRNA 179 inhibit the cAMP-Vfr signaling system. (A) WT PA103 or an *hfq* mutant transformed with the indicated expression vectors were assayed for Vfr-dependent P*_exsA_-lacZ* transcriptional reporter activity. WT PA103 carrying a promoterless *lacZ* transcriptional reporter was included as a control for background activity. *, *P* < 0.05. (B) The indicated strains were transformed with a vector control or an Hfq expression vector and assayed for *vfr*’-’*lacZ* translational reporter activity and Vfr protein levels by immunoblot. *, *P < *0.001. (C) Twitching motility zones for P. aeruginosa strains PA103, PAK, and PA14 carrying a vector control (pJN105) or an Hfq expression vector. *, *P* < 0.05.

We noted that Hfq appeared more effective at inhibiting P*_exsD_*-*lacZ* reporter activity in the WT background (Fig. 2) compared to the *exsA* mutant carrying the rhamnose-inducible *exsA* allele (Fig. 6B). One difference between these strains is that *exsA* transcription is no longer controlled by the Vfr-dependent P*_exsA_* promoter. This raised the possibility that Hfq also impacts the cAMP-Vfr system. Indeed, P*_exsA_*-*lacZ* reporter activity was significantly elevated in the Δ*hfq* background and repressed upon Hfq or sRNA 179 overexpression (Fig. 7A). To confirm that the cAMP-Vfr system is altered by Hfq, we measured the activity of a *vfr-lacZ* translational reporter and Vfr protein levels by immunoblot analysis. Both reporter activity and Vfr protein levels were significantly elevated in the Δ*hfq* mutant and were strongly reduced upon Hfq overexpression (Fig. 7B). In addition to controlling T3SS gene expression, Vfr also regulates type IV pili biogenesis and twitching motility (2). Relative to strains carrying a vector control, overexpression of Hfq resulted in significant inhibition of twitching motility in strains PA103, PAK, and PA14 (Fig. 7D). These combined data suggest that Hfq/sRNAs have two effects on T3SS gene expression, inhibition of Vfr synthesis resulting in reduced P*_exsA_* promoter activity and inhibition of ExsA synthesis.

### sRNA 179 requires RsmY and RsmZ to control T3SS gene expression.

**FIG 8:**
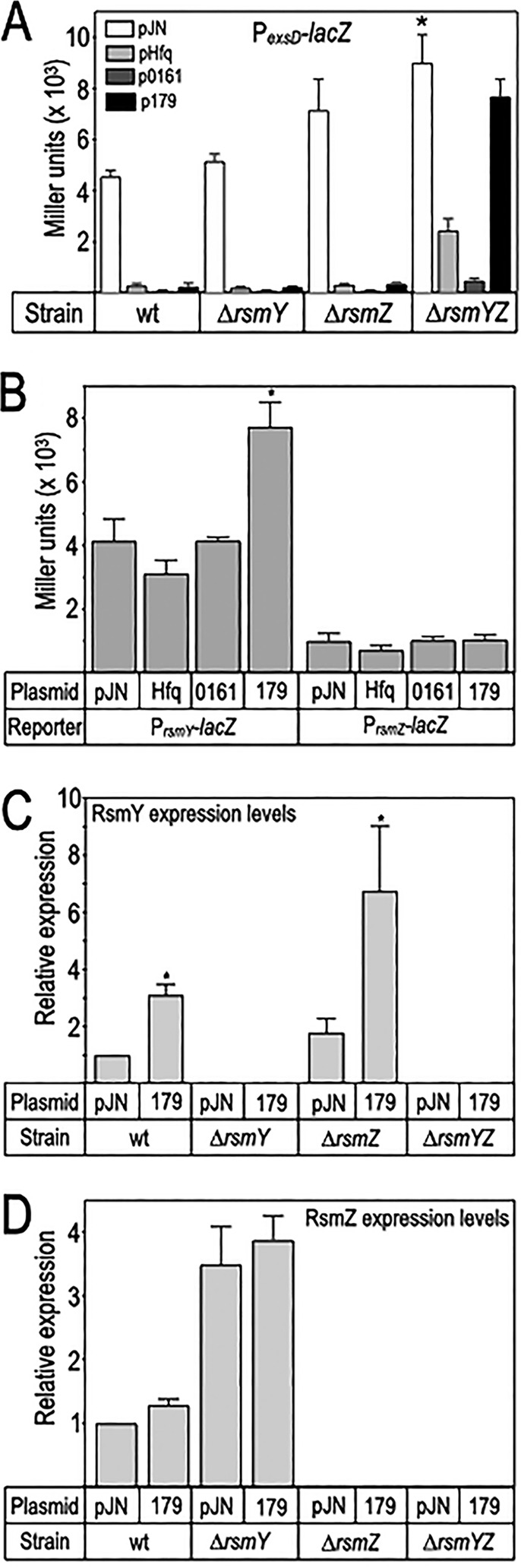
sRNA 179 modulates the Rsm system by increasing RsmY transcription. (A) The indicated strains carrying a P*_exsD_-lacZ* reporter were transformed with either the vector control (pJN), the Hfq expression vector, or sRNA 0161 and 179 expression vectors. Strains were cultured under inducing conditions for T3SS gene expression supplemented with 0.05% or 0.2% arabinose to induce Hfq or sRNA expression, respectively, and assayed for P*_exsD_-lacZ* reporter activity as reported in Miller units. The reported values with the standard error represent the average of at least three experiments. *, *P* < 0.05. (B) WT carrying either a P*_rsmY_-lacZ* or P*_rsmY_-lacZ* transcriptional reporter and the indicted expression vectors were assayed for reporter activity as in panel A. *, *P < *0.005. (C and D) Comparison of RsmY (C) and RsmZ (D) levels in RNA samples harvested from the indicated strains. RNA samples were quantified by qRT-PCR and normalized to the *rimM* housekeeping gene. The reported values are relative to each strain carrying the vector control.

Previous studies have shown that the small RNA-binding protein RsmA is required for the synthesis of both Vfr and ExsA (39). Our finding that Hfq and sRNA 179 overexpression inhibits Vfr and ExsA synthesis suggested involvement of the Rsm system. RsmA availability is controlled by the small noncoding RNAs RsmY and RsmZ (40). Both RNAs possess multiple RsmA binding sites that serve to sequester RsmA from target mRNAs (40). To examine whether Hfq and sRNA 179 function through the Rsm system to control T3SS gene expression, we expressed Hfq, sRNA 0161, and sRNA 179 in Δ*rsmY*, Δ*rsmZ*, and Δ*rsmYZ* mutants and measured P*_exsD_*-*lacZ* reporter activity. Although no significant differences were observed between the WT strain and the *ΔrsmY* and *ΔrsmZ* single mutants, P*_exsD_*-*lacZ* reporter activity was significantly derepressed in the Δ*rsmYZ* mutant owing to increased RsmA availability (Fig. 8A). Overexpression of Hfq, sRNA 1061, and sRNA 179 resulted in strong inhibition of P*_exsD_*-*lacZ* reporter activity when expressed in each of the strains tested, with one exception; sRNA 179 lacked activity in the Δ*rsmYZ* mutant.

We next tested the hypothesis that sRNA 179 stimulates *rsmY* and/or *rsmZ* transcription. Both transcriptional reporter and reverse transcription-quantitative PCR (qRT-PCR) data demonstrate that sRNA 179 expression results in a significant increase in P*_rsmY_*-*lacZ* reporter and RsmY RNA levels (Fig. 8B to D). The effect of sRNA 179 is specific, as reporter activity and RsmY levels were unaffected by Hfq or sRNA 0161 expression, and Hfq, sRNA 0161, and sRNA 179 had no effect on P*_rsmZ_*-*lacZ* reporter and RsmZ RNA levels. These combined findings demonstrate that sRNA 179 has indirect effects on both *exsA* transcription and ExsA translation through modulation of RsmY levels, thus impacting RsmA availability, which itself has positive effects on Vfr and ExsA synthesis.

## DISCUSSION

The previous finding that Hfq influences P. aeruginosa T3SS gene expression led us to investigate the mechanism (29, 30). Potential roles for Hfq include sRNA and mRNA stability control and mRNA translational control with sRNA-assisted targeting (41). We hypothesized the involvement of an sRNA(s) in the regulation of the T3SS. Screening an sRNA expression library resulted in the identification of sRNA 179 as an Hfq-dependent inhibitor of both the T3SS and CVS regulons. sRNA 179 is 134 nt long and located downstream of *acp1*, a putative acyl carrier protein. The reported 5′ end of sRNA 179 immediately follows the *acp1* stop codon (38) and may be generated by a processing event. sRNA 179 lacks an intact open reading frame containing a stop codon. The finding that sRNA 179 activity is Hfq-dependent supports our model that the RNA is the relevant gene product and is consistent with a recent paper showing that sRNA 179 coprecipitates with Hfq (42). sRNA 179 indirectly inhibits ExsA and Vfr synthesis at the posttranscriptional level and likely works through the Rsm system. Data supporting this include (i) the lack of inhibitory activity when sRNA 179 is overexpressed in a Δ*rsmYZ* mutant (Fig. 8A), (ii) increased P*_rsmY_*-*lacZ* transcriptional reporter activity upon sRNA 179 overexpression (Fig. 8B), and (iii) increased RsmY RNA levels upon sRNA 179 overexpression (Fig. 8C). Our working model is that the sRNA 179-mediated increase in RsmY expression titrates RsmA from some target mRNAs, leading to reduced T3SS and CVS expression, as both ExsA and Vfr synthesis are dependent upon RsmA (Fig. 1) (39). The RsmA regulon consists of ∼500 target genes and includes the T6SS (43). To examine whether sRNA 179 has a general effect on the RsmA regulon, we immunoblotted culture supernatants for the T6SS secretion substrate Hcp1 upon sRNA 179 overexpression but observed no change relative to the parental strain (data not shown). This was not entirely unexpected, as we previously demonstrated that T3SS gene expression is more sensitive to changes in RsmA availability relative to T6SS gene expression (39).

sRNA 179 inhibits the T3SS when overexpressed in the WT, Δ*rsmY*, and Δ*rsmZ* single mutants but lacks activity when expressed in the Δ*rsmYZ* mutant (Fig. 8A). These data suggest involvement of both *rsmY* and *rsmZ* in T3SS gene expression by sRNA 179 and may reflect the redundant function that RsmY and RsmZ play in RsmA sequestration. The homeostatic relationship between RsmA availability and RsmY/RsmZ-mediated sequestration of RsmA is a complicating factor. Because RsmA has a positive effect on *rsmY* and *rsmZ* transcription (39), deletion of either may result in a compensatory effect wherein reduced sequestration of RsmA stimulates transcription of the remaining gene. Transcription of both *rsmY* and *rsmZ* is directly controlled by the GacAS two-component regulatory system (44). It is unclear how sRNA 179 stimulates *rsmY* transcription, but modulation of GacAS signaling seems unlikely since *rsmZ* transcription is unaffected by sRNA 179. In addition to GacAS, other factors, including HptB, MvaT, and MgtE, also influence *rsmYZ* transcription (45–47). HptB is a promising candidate, as both HptB and sRNA 179 effect *rsmY* transcription only. While it is clear that *rsmYZ* are required for sRNA 179 activity, they may not be sufficient. Our finding that P*_rsmZ_-lacZ* reporter activity and RsmZ levels are unchanged when sRNA 179 is overexpressed in the *rsmY* background (Fig. 8B and D) suggest additional mechanisms of inhibition by sRNA 179.

In addition to sRNA 179, sRNA 0161 was recently identified with GRIL-seq as another inhibitor of the T3SS (32), and we confirmed that sRNA 0161 is also Hfq-dependent (Fig. 4A). The GRIL-seq data suggest that sRNA 0161 directly targets the *exsA* leader region through imperfect base pairing and is consistent with our data showing that sRNA 0161 inhibits ExsA synthesis at the posttranscriptional level (Fig. 5B and C). In addition, Hfq coprecipitates with the *exsA* leader region (42). The simplest model to account for the inhibitory activity of sRNA 0161 is occlusion of the ribosome binding site. Alternative mechanisms may involve RsmA and DeaD, both positive regulators of ExsA synthesis (22, 39). Rather than blocking ribosome access, sRNA 0161 may instead prevent RsmA and/or DeaD from binding to the *exsA* leader region. Another possibility is that RsmA and/or DeaD activate by displacing/preventing sRNA 0161 from base pairing with the *exsA* leader region. Testing these models will be the subject of future studies.

sRNA 0161 and 179 function independently from one another, and either is sufficient to inhibit T3SS gene expression (Fig. 4C). The primary implication is that together they provide both direct (through the activity of sRNA 1061) and indirect (through sRNA 179) Hfq-dependent mechanisms to inhibit ExsA synthesis. Transcription and synthesis of *exsA* and ExsA are major inputs for upstream regulatory factors (17). Although seemingly redundant, sRNA 0161 and sRNA 179 may differentially coordinate and/or respond to distinct signals that influence T3SS expression. Future studies to understand transcriptional control of sRNA 0161 and sRNA 179 will be important.

Regulatory control of the T3SS by Hfq likely extends beyond the mechanisms described here. Expression of Hfq in an sRNA 0161 179 double mutant still resulted in significant inhibition of T3SS gene expression (Fig. 4B). This may reflect sRNA-independent activity or involvement of an additional sRNA(s) in control of the T3SS. There are over 500 predicted sRNAs in P. aeruginosa (38), only a small fraction of which have been characterized. Most sRNAs require a chaperone to facilitate interactions with their target mRNA. sRNAs that sequester Hfq (CrcZ) and RsmA (RsmY and RsmZ) result in indirect regulation of the T3SS and other virulence factors controlled by Hfq, RsmA, and Vfr (30, 48). Finally, Hfq is known to stabilize RsmY (49). Hfq stabilization protects RsmY from RNase E-mediated cleavage and thus enhances RsmA sequestration by RsmY. Regulation of the T3SS is complex, with numerous regulators that directly or indirectly alter *exsA* transcription and ExsA translation (18–20, 36). Hfq can now be added to that list of regulators, highlighting the complexity of regulatory mechanisms that tightly control T3SS gene expression.

## MATERIALS AND METHODS

### Strain and plasmid construction.

The P. aeruginosa strains and plasmids used in this study are listed in Table S1, and the cloning details and primers are provided in Tables S2 and S3. Routine cloning was performed with E. coli Top10 or DH5alpha cultured in LB-Lennox medium with tetracycline (12 μg/ml) or gentamicin (15 μg/ml) as required. Primers were used to amplify the sRNAs represented in the library (listed in Table S3), followed by cloning into the XbaI/SacI restriction sites of pJN105. The *Δhfq* mutant was generated by PCR amplification of 5′ and 3′ flanking regions using primers N1/N2 and C1/C2 using PA103 genomic DNA as a template. The C1/C2 and N1/N2 PCR products were sequentially cloned into the HindIII/EcoRI and EcoRI/BamHI sites, respectively, of pBluescript SK. The combined flanking regions were then excised from pBluescript SK by digestion with XmaI and cloned into pEX100T. The sRNA 0161 and 179 allelic exchange vectors were generated using the primer pairs listed in Tables S2 and S3 and cloned by isothermal assembly into pEXG2Tc (50). The Δ*hfq*, Δ0161, and Δ179 mutants were generated by allelic exchange and *sacB*-mediated resolution as previously described (51). The *hfq* gene from P. aeruginosa from strain PA103 was cloned into the XbaI and SpeI sites of pJN105 (52).

10.1128/mBio.00363-20.4 TABLE S2 Plasmid construction details. Download Table S2, DOCX file, 0.01 MB. Copyright © 2020 Janssen et al. 2020 Janssen et al. This content is distributed under the terms of the Creative Commons Attribution 4.0 International license.

10.1128/mBio.00363-20.5 TABLE S3 sRNA sequences and primer pairs used for PCR cloning (the underlined sequence is the sRNA sequence followed by 100 nt of downstream sequence). Download Table S3, DOCX file, 0.1 MB. Copyright © 2020 Janssen et al. 2020 Janssen et al. This content is distributed under the terms of the Creative Commons Attribution 4.0 International license.

### Growth curves.

PA103 strains were grown overnight at 37°C in LB containing gentamicin (80 μg/ml) as required. The strains were diluted to an absorbance (A_600_) of 0.01 in a 96-well plate. A_600_ was measured every hour using a Tecan plate reader (Tecan Trading AG, Switzerland) until the stationary phase. Background absorbance measured from wells with only LB was subtracted, and absorbance was plotted against time.

### β-galactosidase assays and immunoblots.

PA103 strains were cultured at 37°C overnight in LB containing 80 μg/ml gentamicin as required. The next day, strains were diluted to an absorbance (A_600_) of 0.1 in tryptic soy broth (TSB) supplemented with 100 mM monosodium glutamate and 1% glycerol. Arabinose was added to induce Hfq (0.05%) or sRNA (0.2%) expression from the P_BAD_ promoter, and rhamnose (0.005%) was added to induce *exsA* expression from the P*_rha_* promoter as appropriate. Cultures were incubated at 37°C and harvested when the A_600_ reached 1.0. β-galactosidase activity was determined as previously described with the substrates ortho-nitrophenyl-galactopyranoside (ONPG) (36) or chlorophenol red-β-d-galactopyranoside (CPRG) (18) as previously described. CPRG activity was determined by measuring product formation at 578 nM and defined as CPRG units (i.e., A_578_/culture A_600_/time [min]/culture vol [ml]) × 1,000. Statistical analyses were determined with one-way analysis of variance (ANOVA) using GraphPad Prism version 5.0c for Mac OS X (GraphPad, La Jolla, CA). Immunoblots using rabbit immune serum to ExsA, Hfq, ExoU, PcrV, and Hcp1 were performed as previously described (36).

### Cytotoxicity assays.

Chinese hamster ovary (CHO) cells (ATCC CCL-61) were cultured in Ham’s F-12 medium (Invitrogen Corp., Carlsbad, California) supplemented with 10% fetal calf serum, 50 units/ml of penicillin and streptomycin, 2 mM l-glutamine, 0.12% sodium bicarbonate, and 2.5 mM HEPES at 37°C in 5% CO_2_. For cytotoxicity assays, CHO cells were seeded at 8 × 10^4^ cells/well into 24-well tissue culture plates (80 to 85% confluence) and incubated for 18 to 24 h at 37°C in 5% CO_2_. P. aeruginosa strains were grown on Vogel Bonner Minimal medium plates overnight at 37°C, washed with PBS, diluted in prewarmed Ham’s F-12 medium, and added to the CHO cells to a multiplicity of infection (MOI) of 10. Plates were centrifuged (500 × *g*, 5 min, 25°C) and incubated at 37°C in 5% CO_2_ for the indicated times. At each time point the plates were centrifuged (500 × *g*, 5 min, 25°C) and 50 μl of the supernatant was transferred to a 96-well plate and assayed for lactate dehydrogenase (LDH) release using the CytoTox 96 nonradioactive cytotoxicity assay (Promega, Madison, WI). The percent cytotoxicity was calculated by subtracting the optical density at 490 nm (OD_490_) of an uninfected control from each sample and using WT PA103 as the positive control normalized to 100%.

### Hfq purification.

E. coli Tuner(DE3) expressing histidine-tagged Hfq(pET23bHfq) was cultured overnight on LB with ampicillin (200 μg/ml). Cell suspensions were prepared and used to start a 2-liter culture at an A_600_ of approximately 0.1 in LB with ampicillin (200 μg/ml). At an A_600_ of 0.5, isopropyl-β-d-1-thiogalactopyranoside (IPTG) was added at 1 mM to induce protein production. Once the culture reached an A_600_ of 3.0, cells were harvested by centrifugation and suspended in nickel nitrilotriacetic acid (Ni-NTA) binding buffer (20 mM Tris-HCl [pH 7.9], 300 mM NaCl, 5 mM imidazole) supplemented with 1 complete protease inhibitor cocktail (PIC) tablet (Roche). Cells were lysed by passage through a microfluidizer at 17,000 lb/in^2^ (Microfluidics, Westwood, MA). Cell lysates were cleared by centrifugation and immediately loaded onto a 1-ml HiTrap His column (GE Healthcare Life Sciences) and eluted with binding buffer containing 300 mM imidazole. The peak elution fractions were pooled and dialyzed against Ni-NTA binding buffer containing 5 mM dithiothreitol (DTT) for 4 h at 4°C. The resulting protein was flash-frozen in 1-ml aliquots. Two 1-ml frozen aliquots of Hfq were buffer-exchanged by diluting to 10 ml with Ni-NTA binding buffer and then concentrated to 1 ml using an Amicon Ultra centrifugal filter (nominal molecular weight limit, 10,000 Da) and repeated five times. The concentrated Hfq samples were then combined and exposed to 0.15 ml of preequilibrated Talon beads (Clontech) for 10 min, rocking at 4 degrees. Beads were then washed with 5 ml of Ni-NTA binding buffer three times and eluted with 10 ml of binding buffer containing 0.5 M imidazole. The eluted Hfq was concentrated again and buffer-exchanged three times to storage buffer (20 mM Tris-HCl [pH 7.9], 300 mM NaCl, 1 mM DTT). Protein concentration was determined using the Bradford assay.

### Electrophoretic mobility shift assays.

RNA was generated from DNA templates encoding the *exsA* UTR region and sRNA 179 by *in vitro* transcription and end-labeled with [γ-^32^] ATP as previously described (24). Purified Hfq_his_ at the indicated concentrations was incubated with the RNA probes in 1× binding buffer (10 mM Tris-HCl [pH 7.5], 10 mM MgCl_2_, 100 mM KCl, 3.25 ng/μl total yeast tRNA [Life Technologies], 10 mM DTT, 5% [vol/vol] glycerol, and 0.1 unit RNaseOUT [Life Technologies]). Reaction mixtures were incubated at 37°C for 30 min and then mixed with 2 μl of gel loading buffer II (Life Technologies) and immediately subjected to electrophoresis on 10% (wt/vol) native polyacrylamide glycine gels (10 mM Tris-HCl [pH 7.5], 380 mM glycine, and 1 mM EDTA) at 4°C. Imaging was performed using an FLA-7000 phosphorimager (Fujifilm), and the images were analyzed using MultiGuage v3.0 software.
