# Supplementary material for: Hfq and sRNA 179 Inhibit Expression of the Pseudomonas aeruginosa cAMP-Vfr and Type III Secretion Regulons
Source: mBio. 2020 Jun 16;11(3):e00363-20. doi: 10.1128/mBio.00363-20 (PMC7298702; doi:10.1128/mBio.00363-20)
Supplement: TABLE S1 [file mBio.00363-20-st001.docx]

Table S1. Strains and plasmids used in this study.

**Strain/Plasmid Relevant characteristics Source**

*Strains*

*E. coli*

DH5alpha *supE44* D*lacU169* (f80 *lacZ*D*M15*) *hsdR17* (1)

SM10 *thi thr leu tonA lacY supE recA*::RP4-2Tc::Mu Km (2)

Top10 F- *mcrA* Δ( *mrr-hsd*RMS-*mcr*BC) Φ80*lac*ZΔM15 Δ*lac*X74 *rec*A1 Invitrogen

*ara*D139 Δ(*araleu*)7697 *gal*U *gal*K *rps*L (StrR) *end*A1 *nup*G

*P. aeruginosa*

PA103 wt parental strain isolated from human sputum (3)

PA103 ∆*hfq* deletion of *hfq* This study

PA103 ∆*exsA* In-frame deletion of *exsA*  (4)

PA103 ∆*exsD* In-frame deletion of *exsD* (5)

PAK wt parental strain (6)

PA14 wt parental strain from a burn wound (7)

PA103 ∆0161 deletion of sRNA 0161 This study

PA103 ∆179 deletion of sRNA 179 This study

PA103 ∆0161/179 deletion of sRNAs 0161 and 179 This study

PA103 ∆*exsA*, ∆*hfq* In-frame deletion of *exsA* and *hfq* deletion This study

PA103 ∆*exsA*/0161/179 In-frame deletion of *exsA* and sRNAs 0161 and 179 This study

PA103 ∆*rsmY* deletion of *rsmY* This study

PA103 ∆*rsmZ* deletion of *rsmZ* (8)

PA103 ∆*rsmY*/*rsmZ* deletion of *rsmY* and *rsmZ* This study

*Plasmids*

pJN105 Arabinose inducible expression vector (9)

pBlueScript SK Standard cloning vector Stratagene

pEX100T Cloning vector for allelic exchange (10)

pHfq Hfq expression vector, pJN105 backbone This study

p0161 sRNA 0161 expression vector, pJN105 backbone This study

p179 sRNA 179 expression vector, pJN105 backbone This study

pEXG2Tc Cloning vector for allelic exchange (11)

pEXG2Tc ∆0161 Allelic exchange vector for construction of ∆0161 mutant This study

Table S1. Strains and plasmids used in this study (cont).

**Strain/Plasmid Relevant characteristics Source**

pEXG2Tc ∆179 Allelic exchange vector for construction of ∆179 mutant This study

pEX18Gm ∆*exsA* Allelic exchange vector for construction of ∆*exsA* mutant (4)

mini-CTX-lacZ Cloning vector for chromosomal integration at CTX site (12)

mini-CTX-P*_exsD_*-lacZ mini-CTX-lacZ with P*_exsD_*-lacZ fusion (5)

mini-CTX-P*_exsA_*-lacZ mini-CTX-lacZ with P*_exsA_*-lacZ fusion (13)

mini-CTX-P*vfr’-‘lacZ* mini-CTX-lacZ with P*vfr’-‘lacZ* This study

mini-CTX- P*_rsmY_*-lacZ mini-CTX-lacZ with P*_rsmY_*-lacZ fusion (8)

mini-CTX- P*_rsmZ_*-lacZ mini-CTX-lacZ with P*_rsmZ_*-lacZ fusion (8)

pUC18-mini-Tn7T-Gm-LacZ10 standard Tn7 cloning vector (14)

pScRha source of RhaR and P*_rha_* promoter (15)

pUC18-mini-Tn7 P*_rha_*-*exsA* rhamnose inducible *exsA* expression This study

pUC18-mini-Tn7 P*_rha_*-*vfr* rhamnose inducible *vfr* expression This study

pET23b E. coli expression vector Novagen

pET23b-Hfq Expression vector for histidine-tagged Hfq This study

**REFERENCES**

1. Hanahan D. 1983. Studies on transformation of Escherichia coli with plasmids. J Mol Biol 166:557-80.

2. Simon R, Priefer U, Puhler A. 1983. A broad host range mobilization system for in vivo genetic engineering: transposon mutagenesis in Gram negative bacteria. . Nature Biotechnology 1:784-791.

3. Liu PV. 1966. The roles of various fractions of Pseudomonas aeruginosa in its pathogenesis. 3. Identity of the lethal toxins produced in vitro and in vivo. J Infect Dis 116:481-9.

4. Intile PJ, Balzer GJ, Wolfgang MC, Yahr TL. 2015. The RNA Helicase DeaD Stimulates ExsA Translation To Promote Expression of the Pseudomonas aeruginosa Type III Secretion System. J Bacteriol 197:2664-74.

5. McCaw ML, Lykken GL, Singh PK, Yahr TL. 2002. ExsD is a negative regulator of the Pseudomonas aeruginosa type III secretion regulon. Mol Microbiol 46:1123-33.

6. Takeya K, Amako K. 1966. A rod-shaped Pseudomonas phage. Virology 28:163-5.

7. Rahme LG, Ausubel FM, Cao H, Drenkard E, Goumnerov BC, Lau GW, Mahajan-Miklos S, Plotnikova J, Tan MW, Tsongalis J, Walendziewicz CL, Tompkins RG. 2000. Plants and animals share functionally common bacterial virulence factors. Proc Natl Acad Sci U S A 97:8815-21.

8. Intile PJ, Diaz MR, Urbanowski ML, Wolfgang MC, Yahr TL. 2014. The AlgZR two-component system recalibrates the RsmAYZ posttranscriptional regulatory system to inhibit expression of the Pseudomonas aeruginosa type III secretion system. J Bacteriol 196:357-66.

9. Newman JR, Fuqua C. 1999. Broad-host-range expression vectors that carry the L-arabinose-inducible Escherichia coli araBAD promoter and the araC regulator. Gene 227:197-203.

10. Schweizer HP, Hoang TT. 1995. An improved system for gene replacement and xylE fusion analysis in Pseudomonas aeruginosa. Gene 158:15-22.

11. Jorth P, Staudinger BJ, Wu X, Hisert KB, Hayden H, Garudathri J, Harding CL, Radey MC, Rezayat A, Bautista G, Berrington WR, Goddard AF, Zheng C, Angermeyer A, Brittnacher MJ, Kitzman J, Shendure J, Fligner CL, Mittler J, Aitken ML, Manoil C, Bruce JE, Yahr TL, Singh PK. 2015. Regional Isolation Drives Bacterial Diversification within Cystic Fibrosis Lungs. Cell Host Microbe 18:307-19.

12. Hoang TT, Kutchma AJ, Becher A, Schweizer HP. 2000. Integration-proficient plasmids for Pseudomonas aeruginosa: site-specific integration and use for engineering of reporter and expression strains. Plasmid 43:59-72.

13. Marsden AE, Intile PJ, Schulmeyer KH, Simmons-Patterson ER, Urbanowski ML, Wolfgang MC, Yahr TL. 2016. Vfr Directly Activates exsA Transcription To Regulate Expression of the Pseudomonas aeruginosa Type III Secretion System. J Bacteriol 198:1442-50.

14. Choi KH, Schweizer HP. 2006. mini-Tn7 insertion in bacteria with single attTn7 sites: example Pseudomonas aeruginosa. Nat Protoc 1:153-61.

15. Cardona ST, Valvano MA. 2005. An expression vector containing a rhamnose-inducible promoter provides tightly regulated gene expression in Burkholderia cenocepacia. Plasmid 54:219-28.
