# Supplementary figures and images for: Hfq and sRNA 179 Inhibit Expression of the Pseudomonas aeruginosa cAMP-Vfr and Type III Secretion Regulons
Source: mBio. 2020 Jun 16;11(3):e00363-20. doi: 10.1128/mBio.00363-20 (PMC7298702; doi:10.1128/mBio.00363-20)

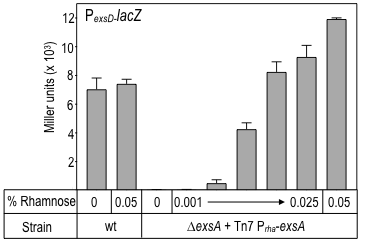

Supplement: FIG S1 [file mBio.00363-20-sf001.tif]

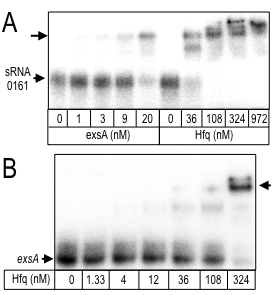

Supplement: FIG S2 [file mBio.00363-20-sf002.tif]
