# Supplementary material for: Hfq and sRNA 179 Inhibit Expression of the Pseudomonas aeruginosa cAMP-Vfr and Type III Secretion Regulons
Source: mBio. 2020 Jun 16;11(3):e00363-20. doi: 10.1128/mBio.00363-20 (PMC7298702; doi:10.1128/mBio.00363-20)
Supplement: TABLE S2 [file mBio.00363-20-st002.docx]

Table S2. Plasmid construction details.

Plasmid Primer Pair Vector

Hfq pJN105

p0161 165361355-165361356 pJN105

p179 156853106-156853107 pJN105

pEXG2Tc ∆0161 167509086-167509087 and 167509088-167509089 pEXG2Tc

pEXG2Tc ∆179 165555383-165555385 and 165555384-165555386 pEXG2Tc

mini-CTX- P*vfr’-‘lacZ* 132725051-132725052 mini-CTX-lacZ

pUC18-mini-Tn7 P*_rha_*-*exsA* 196423235-196423236 and 196423237-214219975 pUC18-mini-Tn7T-Gm-LacZ10

pUC18-mini-Tn7 P*_rha_*-*vfr* 214512370-196423236 and 196423237-202175347 pUC18-mini-Tn7T-Gm-LacZ10

pET23b-Hfq 144514727-144514728 pET23b

Primers used in this study

Primer ID Primer name Primer sequence (5’ to 3’)

165361355 Sr0161 Xba GGATCCACTAGTTCTAGAGCTCAATGGATAATTGTCGCTGGCTG

165361356 Sr0161 Sac CTATAGGGCGAATTGGAGCTCCGGAAAGCGTGTTGTGGTGTCGTTCC

156853106 Pant179For ggatccactagttctagaCTCCCCCTGGACCACCGGCCGAT

156853107 Pant179Rev ctatagggcgaattggagctcCCCTTGTTCGGTCGACCGGCGAG

167509086 5UpDel0161Hind GACTAAATGTAAAGCAAGCTTAGCACGGCGTGCCACGCTCGGT

167509087 3UpDel0161 AAAGGCCGCTGGTATCAGCGGCCTTCCACGTCCCGCTCTTGCATGG

167509088 5DownDel0161 CCATGCAAGAGCGGGACGTGGAAGGCCGCTGATACCAGCGGCCTTT

167509089 3DownDel0161Xba CGAGCCCCGGGGATCCTCTAGACCTTCTGCAGCACCGCGGTGACG

165555383 Pant179 US Hind GCATAAATGTAAAGCAAGCTTCAGCCCCGGTACTGATGGAGAAGCC

165555384 Pant179 DS Sac TAAGGTACCGAATTCGAGCTCAAGGGAAACCTTGAAAGCCAGCAGCC

165555385 Pant179 US GGCTGCCTCGCTACCGCGGTGGTCAGGTCGGCACGGCTTCCTCGAG

165555386 Pant179 DS CTCGAGGAAGCCGTGCCGACCTGACCACCGCGGTAGCGAGGCAGCC

196423235 exsA 101 for Prha fusion ATTCAGGCGCTTTTTAGACTGGTCGCACGTGCTCATGGCTTTGAAAATCAA

196423236 pRha for 101 cloning TTGATTTTCAAAGCCATGAGCACGTGCGACCAGTCTAAAAAGCGCCTGAAT

196423237 pRha for Tn7 cloning AAGCTAAATCGATCATGCATTTAATCTTTCTGCGAATTGAGATGA

202175347 vfr UTR Pml TTTTAGACTGGTCGCACCGCGGTTTACTGGCACACTTCCTGAT

214219975 exsA SacI for Tn7 Rha ACCATCCAGTGCAGGAGCTCTCAGTTATTTTTAGCCCGGCATTCG

214512370 vfr 3’ Sac Gibson ACCATCCAGTGCAGGAGCTCTGGTGCTGGGCGCCGATCCTGACTGATCC

144514727 5 Hfq his6 pET23b CCACAACGGTTTCCCTCTAGATACGATCCTTAAAGGAGTGCGGCA

144514728 3 Hfq His6 pET23b ACGGAGCTCGAATTCGGATCCGCGTTGCCCGGCTCGGCCGGCT

NA N1 GTCGGATCCCGGGTGTCGATGAGCGACCTG

NA N2 GGAGAATTCATGTGCCGCACTCCTTTAAG

NA C1 CCTGAATTCGCTTGACGGGAGTCCGCTTTGTTC

NA C2 GCGAAGCTTCCCGGGTGGCGAACAACTGGTTG

132725051 Vfr_5'ecoRI_3UY51 CGTATAATGTGTGAATTCGGTTTACTGGCACACTTCCTGATC

132725052 Vfr_3'_bamHI_3UY51 TGTAAAACGACGGGATCCACTTCGCATTCCACCTTGGCACGA
