# Supplementary material for: Hfq and sRNA 179 Inhibit Expression of the Pseudomonas aeruginosa cAMP-Vfr and Type III Secretion Regulons
Source: mBio. 2020 Jun 16;11(3):e00363-20. doi: 10.1128/mBio.00363-20 (PMC7298702; doi:10.1128/mBio.00363-20)
Supplement: TABLE S3 [file mBio.00363-20-st003.docx]

**Table S3. sRNA sequences and primer-pairs used for PCR cloning.** The underlined sequence is the sRNA sequence followed by 100 nt of downstream sequence.

**Pant3**

GTTAGGTTTTTCGGCACGCCCAGATGGCCTTGTGCCTTACCTCCGGATTGTCCGACTCATGGAAGACGGATTTCGGAATATTCCTGCTTACCCGCTGTAGCCGCTGGGCCAACATCGCGCCTTTCTGTATTTCAAGGAATGAAATCGTGGCTCTCCATCGCCTGGCCTTTATCTTCCTGTGCCTGCCGCTCATGGCCAGCGCCGCCCCCTTCACCTCCCCCGGCGACCGCGACCTGATCCGCGA

F – ggatccactagttctagaGTTAGGTTTTTCGGCACGCC

R – ctatagggcgaattggagctcTCGCGGATCAGGTCGCGGTCGCC

**Pant9**

ACGGGTGCGAAGTCCGGCCGTGCACCCACCGACGCCCCACAACAGCAAGAACAACGATCGGTGCATCCATGGCAAAGGACCTGACTTTCCCAGACGCCTCCGCGTCGTCCCGCAACCCTCCCTCCGCGCGCCTCCGTCTACCGGCGACACGCGCCCCCTAGCACCGCCCTCCCGTCCGCCACCGCAGCAACCCGCGACGCACCTTGCGTCGGTCCCGGCCCCCGCCGCCGGCGGGCGCCGCTCGCCCGCGCTCCG

F – ggatccactagttctagaACGGGTGCGAAGTCCGGCCGTGC

R – ctatagggcgaattggagctcCGGAGCGCGGGCGAGCGGCGCCC

**Pant13**

TTTCGCGTCACCTTTCGCCGTTCCTGCCATGGACGACCTCTGGGAAGCCCCGTTCCGGGGCTGAACGGGTCGCTATTACAAGAAAAAACACACTGCGCACACACAACCGGCGCCGGGCCTGGTCCTGGCGTCGCTCCCTCGTCGTGGAAAAAATTCGCAGGAGAACTGTGCCATGCATGTTTCAAAGGTGTTTCCGTCCC TCCGGGACACGTTGCCGCGGGACCTGATGGCCTCCGTGG

F – ggatccactagttctagaTTTCGCGTCACCTTTCGCCGTTCC

R – ctatagggcgaattggagctcCCACGGAGGCCATCAGGTCCCGC

**Pant17**

GTTTGATGGGGGTCAACGGACGCCTCCCGGCAGGGGCATAGAGTGGGGGACTGCATAGCTGAAAACGTAGAACGGAAGGAGTCCCGCATGAGCGAAGAACCCACTGTCAGTCCCCCCTCCCCCGAGCAACCCGCCGCGCAGCCGGCCAAGCCGGCCCGGCCAGCCGCCCGCCGCGCCCCGCGCAAGCCGGCGACCCGCCGCCCGCGAGTGGCCAGCCCGGCGCAGAAGGCCC

F – ggatccactagttctagaGTTTGATGGGGGTCAACGGACGCC

R – ctatagggcgaattggagctcGGGCCTTCTGCGCCGGGCTGGC

**Pant18**

CTACCCATCGCCCTCCGGGAGTAATGTCAGGTACCCATCGGCGGGTTCTGGGTACGCCCTTGCCGCTCCGGCAAGGGCGCCCTTCCTTCCCGCGCGATCGATGCGCCTAGAAGTCGAAGAACACCGTCTCGTTGTCGCCCTGGATGCGGATGTCGAAACGGTAGGCGAGCTGTCCGTCCACT

F – ggatccactagttctagaCTACCCATCGCCCTCCGGGAGTA

R – ctatagggcgaattggagctcAGTGGACGGACAGCTCGCCTACCG

**Pant19**

GTCGCCGTAGCAACGCCGGGCATACCCTGCCCGGCGTCGGGGCGGATGGCCCACCCGCCGTCCGCTCTCCTTCCCCCCGCACAGACTCTGCTACGGCGGCGCCCCCTTCCCCTTACGCCGCGCTCAGCGTCCCGGTTCCGGAATCCGGTACCAGGCGACGTACAGCGCCGGCAG

F – ggatccactagttctagaGTCGCCGTAGCAACGCCGGGCAT

R – ctatagggcgaattggagctcCTGCCGGCGCTGTACGTCGCCTG

**Pant20**

GCTTCTCGCCCGAACGCCGGGGCGGTAATGTGTAGAAAGACCACGCACTGTTTTGTGTACAAAAGTCTGCTGATTCTTTTTGTCACAAATCGTTTTTGTGTATCCAGTTTTCTTCGCGCTGCCAACCCAAAGTCGAGCACGCCCCTCCCGACCACGCACCAGTGCAAGGCAAAAACTGTCCTTGGGGTCAGGTTTTTGCCGTCGTAGAGAGGTTGGAGGCATTTCGCCGCCTTT

F – ggatccactagttctagaGCTTCTCGCCCGAACGCCGGGG

R – ctatagggcgaattggagctcAAAGGCGGCGAAATGCCTCCAACC

**Pant21**

CTGTCCTTGGGGTCAGGTTTTTGCCGTCGTAGAGAGGTTGGAGGCATTTCGCCGCCTTTGTCCAAAACACTGAAAAAACAGGCGTTCCCTTCGCCGCAGGAAACCCGGCGAAGCGCCCGCGCCGTCTTGGCTTCGTTCTTGCTATCCATGGCGAGGCCTGACCGCTCGGACAGGTTCGCCCACTAGAAGAAAAGCTATTCACTCCAGGAGCGTTTCAATGAGCCGCACACTCGCCACCGTGA

F – ggatccactagttctagaCTGTCCTTGGGGTCAGGTTTTTGC

R – ctatagggcgaattggagctcTCACGGTGGCGAGTGTGCGGCTCA

**Pant22**

CGACGATGAGCCGCGAACGCGAACTGGACGCCTGGATCGACGGCCTGCTGGCCGATCCGCAGTTCCACGGGCATCCGCTGCACCAGGCGCTGGCCCGCCTGCGCCAGCAATCGCTGGAGCAACTGGTGCGGCTGGAGCGCATCGCCCGGATCTCCGACGGTTTCCAGTCGATGGCGCGGGAGCAGAACCTGTCGCTGTCCGAGCGCTACCACAAGC

F – ggatccactagttctagaCGACGATGAGCCGCGAACGCGAA

R – ctatagggcgaattggagctcGCTTGTGGTAGCGCTCGGACAGC

**Pant23**

GCTATCCCTATCAGTTTCTCGCCCGGCCCGCGGCCGAAATCATGGATCGAAAGCGTGTGTCGAACTTTTGGCGAAAGGGCCAATTGTGCAGATGTGCATTATTTCAGGCAACGTAATTTGGTTTTATTTCGCCATTAATTTGACGTCACAATGATGGCGAGCAGGCAAAGGCCC

F – ggatccactagttctagaGCTATCCCTATCAGTTTCTCGCCC

R – ctatagggcgaattggagctcGGGCCTTTGCCTGCTCGCCATCA

**Pant25**

GCTCTTATTCCGTCAACAAAGAACAAATTAGAAAAATATGCAATTTAGTAATAAGCATAGATAGACCCCAGGAGCTGAACATGGTCCACCGTGCCTTTGCCGCCCCACCTCCGCCGTTCCGAATGCCCTTCCGCGTCGCCGGCGGCTGTCGCCCCTGACAGTCGCCCGGGGCTGAGCCGTCCGCCGGCCCCCTTCGAACCTTTCGC

F – ggatccactagttctagaGCTCTTATTCCGTCAACAAAGAACAAAT

R – ctatagggcgaattggagctcGCGAAAGGTTCGAAGGGGGCCGG

**Pant31**

CTGTGGGAGTCCTGGTAGGAACGGCATCCGAGCATGCAGAACTATAGACGTGAAACGGCCAGCCCCGAGGAACTATCCGCCGATCGACCCTCCATCTGGCCCCGGCCTTGGGTGCCATTCCCGGCCGGACAGGATCGGTTGCTCTGGTCTCTGCGGCGAGGGACAGGCGCTGCTCGGCGGAGCGGGTGGCGGTGAGGCAGGTCTTGTCTAGTCCTGAAATAGGTTTACACCTGCTCAGGTAGGCCGATAGGCCTCAAAACGCCCGATGCACCGCATCGGGCGTTTTGTTT

F – ggatccactagttctagaCTGTGGGAGTCCTGGTAGGAACGG

R – ctatagggcgaattggagctcAAACAAAACGCCCGATGCGGTGCAT

**Pant32**

TCAGCTTTCTCTCTCGTTGGCGTCCTTTTGAACTGCCCTGTGGGGCAGGCGGCCGAGTCGCCGCTATCCTCGATGCGCTGCTTTGCCAGTCCGGCCACGTCCGTTACCGCCTACGCCCCGGCCAAGGAGGCCACCATGTCCGCTGATGCTTATCACCCACCGACAACTTCGCCGCGCCTGGAAACCCTGGACGTGCTCAGCATAGGCATGAGCCTGGATGTGTTTCGCCAGGGGC

F – ggatccactagttctagaTCAGCTTTCTCTCTCGTTGGCGTC

R – ctatagggcgaattggagctcGCCCCTGGCGAAACACATCCAGG

**Pant39**

TTTTGCCCGTTCGTCCAGACGCTTCCTCCTGGGGGCCCGACGACCGGACGCAGTATCGACGACGAAGACGCTGCCATGGGCGCTGCGCCAATCTGCCGCGCCGGGGCCGGTCGTGTCGCTCGCACCGGGGCCGAGGGCCCGGAAAACCTGAAACCAACACGCCGGTAGGGAACCCAGCTCACTCATGTTTCCCGGCGGAATCGTCGGGTTGTCATG

F – ggatccactagttctagaTTTTGCCCGTTCGTCCAGACGCTT

R – ctatagggcgaattggagctcCATGACAACCCGACGATTCCGCCG

**Pant40**

CGCACCGAGTCGGACAAGCTCAGCAAGGAAACCCTCAAGCTCCTGCTCGACTACGCCCAGGCCTACAACAGCGGCAACGTCAGCGACGACCAGGCGAGCAAGCTGATCGAGCGTGTCGACGACCTCCAGGAGGACCGTCTGGAACTGCGCGACAAGTACGTCAAGCGCATCGCCAAGAACGTCTCGCCCAAGCGCGCCATGCGCTTCCTGCAGATCGAGATCCAGCTCGACGCCATCGCCACCCTGGAGATCGGCCGTCAGGTACCGCTGGTCGAGTAAC

F – ggatccactagttctagaCGCACCGAGTCGGACAAGCTCAG

R – ctatagggcgaattggagctcGTTACTCGACCAGCGGTACCTGAC

**Pant41**

CCGTTTTCCGTGTGAATGCCATGACTCCCCGCCGCAAGACGCTCCTCGTCAGCCTGGTCGGCCTGCTCTGGGCCGGCGGCCTGCTCGCCGCCTACTGGTGGTTCGAGATCCGCTACATCCGCCCGTTCAGCGAGCAGACCACCCTGTTCTCCGGCGACAGCCTGCGCCTGCCGGCCGAGCTGGCCGGGCCGGGGGCGATCCGCCTGGTGCATTTCTGGGACCCGGCCTGCCCCTGCAACGTCGGCAACCAGCAGCACCTCGGCGAGCTGATCGAACGCTTCGCCGGCAAGGGC

F – ggatccactagttctagaCCGTTTTCCGTGTGAATGCCATGAC

R – ctatagggcgaattggagctcGCCCTTGCCGGCGAAGCGTTCGA

**Pant44**

TAGAGTCGAACCGCGCCCTCCTCGAGGGGTCGCCCGGAGCCGCGGAAACGGCGCCGGCAGAGCCCAAGGGAACCTCGCGACCGCGAGCTAGAAATCCAACAAGAACGCTTCCTGAGAACGCCATGAACACTATCGACGCCCCAACGCCCGTGCCCGCACACGCCGCGCGCCCTCTCGTCACTGCCATTTCCACCGACCCGGCGGAAACCCCAGCGGTTTTCCACTAGCCCGTCCA

F – ggatccactagttctagaTAGAGTCGAACCGCGCCCTCCTCG

R – ctatagggcgaattggagctcTGGACGGGCTAGTGGAAAACCGCT

**Pant45**

GGAAACGGGCTGCGCCCGACCTTTCCTACGCAAGCTACGGCTCCAGGCTGAAGCCAAGCTTAAAGTCAGCCAGCCGCCCGGGAGACTCGGACAGGTCCGAGTTTCCTGTCCGAAAGAGACGACAAGAGAGCATCCGCAGTCTTCTTCCAGCGCCACCAGATTGGCCTGTTCGATCCCTTATTGGCCGCATCTATCGTTCTGCGCCCGCGGCGCCCCGGTCATTATCGAACCGCAGCAGGTGAAGCGGGCCCCAGAAACCGCAAAACCTCAACAATAACAACTGCT

F – ggatccactagttctagaGGAAACGGGCTGCGCCCGACCTT

R – ctatagggcgaattggagctcAGCAGTTGTTATTGTTGAGGTTTTGC

**Pant47**

TCGTGAGCGTCGAAGGAACCGGATGCAAACGGGGAATAGCCGAGTGTACGGAGTTGGCCGGGGTCTGAATGGGGTCACCATTGGCGACGGCCTGGGCTCACTGCCCGCCGGGGGATGATTGGCGAGGAATGCCCGGATTCTAAGCCCAGAGCGGGCGAATGGGGAAGGCTGGCGGTCGGCGGAGCGGCGCGTCGCGACCAGGGTTGGCCTCTTGCGCCGCCCGTCGCGCCG

F – ggatccactagttctagaTCGTGAGCGTCGAAGGAACCGGAT

R – ctatagggcgaattggagctcCGGCGCGACGGGCGGCGCAAGA

**Pant49**

CCGCGAGGTCGGCCCACTCGACTGCACCCTGGAGAGTGCCGGCAACCCATTTTTCCTGCCGTCCCTGGCCCGCCTCTGGCAGCGCCAGGCCGGCTATCGCTGGCACGGCATCAGCGGCGAGCGCCTGTCGGGCTGGCAGGACGACTGGCTGGTGGTGGCCGACCAGGGCGCCGACCCGTTCATCCTCGAGACCGGCAGCGGGCGCATACTCTTCGACCTGCATGGCGGCAGGGGCTGGGACCCGGCGCCGTGCT

F – ggatccactagttctagaCCGCGAGGTCGGCCCACTCGACT

R – ctatagggcgaattggagctcAGCACGGCGCCGGGTCCCAGCC

**Pant53**

TTTCGTCGCTAAAAAGTTGGCATGTTGAGGGATTTGCCGATCCGGACACGCGCGGGGAAGGCGAGAGCCACTGGACTAGACTGAGAAGTGCGCGGCGGGGCCGGTCGGTTGCCGGCCCGGTGCGCGGGACGAGCGCCTTCGTCCCGGGGTGGCCCGGCCGGGCGGTCGGGGAGGTCGATATGCGGATGCCGTGCCGGCTCGCGCTGGCGCTGTTGCTGTGCTTCGCCGGCCCGGCCTG

F – ggatccactagttctagaTTTCGTCGCTAAAAAGTTGGCATGTT

R – ctatagggcgaattggagctcCAGGCCGGGCCGGCGAAGCACA

**Pant54**

GTACCGTTCGTCCGAAAAACGAAAAAAGCGCGTTTCTGTATTTTTATACAGGTTCGCTTTCCTCAGTTTTTTGTCGTAGAGTGCTGACACTGTGTTTGCATGGGTCGCCGTCGATCGTGACCTGATGCGGTCGGAAATTCCAGCCCGTCCTGCTCGACTCCTTGTAACTCCTTGCAACTCAGTTA

F – ggatccactagttctagaGTACCGTTCGTCCGAAAAACGAAAAA

R – ctatagggcgaattggagctcTAACTGAGTTGCAAGGAGTTACAAG

**Pant59**

CCGGAGCTGGACCGCACCCTGCAGGCGAAGATGGACACCGGCGCCTACACCTCCTCGCTGTCGGCCAAGGACATCGAGTTGTTCCAGCGCGACGGCGAGGAGTGGGTGCGCTTCCGGCTGGCCACCAAGGAGGCCGACGGCTCGGTGTTCGAGCACAAGCTGGCGCGCATCGGCAAGATCAAGAACCGCGCCGACAACCGCAGCGGCGAGGACGAG

F – ggatccactagttctagaCCGGAGCTGGACCGCACCCTGCA

R – ctatagggcgaattggagctcCTCGTCCTCGCCGCTGCGGTTGT

**Pant60**

TTCGAGCACAAGCTGGCGCGCATCGGCAAGATCAAGAACCGCGCCGACAACCGCAGCGGCGAGGACGAGGATGAGGACCGCCTGAGCGAACGCCCGGTGATCGACCTGCAGGTCTGCCTGGGCGGAGCGATGAAGACCATCGAGGTCAACCTCACCGACCGCAGCGCCTTCAACTATCCGTTCCTGATGGGCACCAAGGGCTTGCGCAAATTCCACGTCGCGGTCGATCCCTCCGAGC

F – ggatccactagttctagaTTCGAGCACAAGCTGGCGCGCATC

R – ctatagggcgaattggagctcGCTCGGAGGGATCGACCGCGAC

**Pant61**

TTTTGTGAGCAACTGTGGATGAACCGGAAGAGCCATCGATCAAAAAACGTACGGATACTTGGAGTGCCGATGGATAAAGGCCTGCATGGATTCTTCCCCGGCTTACCCACAGACTGCTCCACGTTATTTGTGCACAGGGGCAATGACTGCGGCGAAGGTTAATCCACAAGAAAAAGCGCAAGAGAGGCTGCTCAGAAAACGAGCAAACCCTTGTATCG

F – ggatccactagttctagaTTTTGTGAGCAACTGTGGATGAACC

R – ctatagggcgaattggagctcCGATACAAGGGTTTGCTCGTTTTCT

**Pant62**

CTGAACAGGCGCTTTGCAGGGCCTCTTCCGACCAGTTATCCACGCGCCTGCGGGGAGGAGAAGGCAGGTCTATGCACAAAGTACGTGCATGAACCTGTGGAAAAGCCTGGGGTGGATCGCTGAGCGTCAGTATTGACAGGGGCTAGGAAGGAGTGCGCGTTTTTTCGCCAGGCTTGCGGAGTTCCGCAAGCCTGGGGATAACGTCAGCCCGGTTGCGGCAGGCGGCGCAGGCTGCGCCAGGCGTCGAAGCTGTAGACGGCCAGGGCGAGCCAGA

F – ggatccactagttctagaCTGAACAGGCGCTTTGCAGGGCCT

R – ctatagggcgaattggagctcTCTGGCTCGCCCTGGCCGTCTAC

**Pant64**

CTTTGTCACGCCGGCTGCCGCCTGCCTCATCCGCCTGCCAGGACGCTCGGCCGAGCCGTCGTCCGGCAACCGCACCGGCCGTTCGATCAAGGAGCCAACCGATGTCCATTCGTCCTTCACTGCCGCTGGCCGGGCTGCTGCTCGCCGTCGCCCTGCCGCTCTCTGCCGCCCAGCCGGGCGCCGCCAAAGGCGCCCCACTCA

F – ggatccactagttctagaCTTTGTCACGCCGGCTGCCGCCT

R – ctatagggcgaattggagctcTGAGTGGGGCGCCTTTGGCGGCG

**Pant66**

TCAGGCCATCCAAAACAGGGGAGAGCCGGCACACCGGCTTCCGGGCCATCGGCTGTGACCGTTAGGAGCGCTGGCGGAAAGGGAAGCACCGTGCAGCGACGGAGTCTGGGGGATTCTCGCCGGCCTGGCTGGCGGCCCCCCCGCACCCGCACGACCGATCCCGCGCCGGCTCACTGCATCGAGCGAGGAGACGTCATGAGCATTTTCAGTCACTTCCAGGAACGCTTCGAAGCGACCCGCCAAGAGGAAT

F – ggatccactagttctagaTCAGGCCATCCAAAACAGGGGAGA

R – ctatagggcgaattggagctcATTCCTCTTGGCGGGTCGCTTCGAA

**Pant68**

ATTACGTATCTATCGAGAGAATGGGACCGCCGCTGTAGATATAACTGACAGGGCGTTGCGGGTTGTATATTCCAGACGGGTTGATGCAGTTTCGAAAGGAGAAGCTGCCACGCCCGGCTTTGGCCCGAACAATGCTTCCGTCTACGTGATTGGTGATGAGCACAATAAGCGTCCGGTCTGGGCCCGAATGGGTGATGGCGTTGTTCAATGGGGCTACGAGGATTGGTGGCCATCACTATTCCATACTTCTGGAACCTTATATGTGGTGGCCAAGGTATGAGTGAGTAT

F – ggatccactagttctagaATTACGTATCTATCGAGAGAATGGGA

R – ctatagggcgaattggagctcATACTCACTCATACCTTGGCCACCA

**Pant69**

ATGGCTCCGTGAGACCGCTATCGTGATCCACCTCAGCAGCATCATCACCTCCCTCATCAAGGCATTCGCCCGTTGGCGCTGGCGCGCCTGACTTCTTCTTCGCCGGCCTGGCCGGTCACGTCTCCCTGCGTACCCCGTTTTCCACAGGCATAATGCCGACGATCCACTCCCCCTTCCTCCGCGAGCGCGCCGGGAAACGGGTGTGGCGAAAGC

F – ggatccactagttctagaATGGCTCCGTGAGACCGCTATCGT

R – ctatagggcgaattggagctcGCTTTCGCCACACCCGTTTCCCG

**Pant70**

GGTTTACTGGCACACTTCCTGATCTGCCCGGTTGGGGGGAGCTCTCCGCTGAGCTCCCGGGCCCTGGCAGGTCTCTCCATAAGAACTCAAAAAAACATCGCCTTGTACGTCAGGCATATTCTTTATTCTTTCGGGACTCGGGCATGGTAGCTATTACCCACACACCCAAACTCAAACACCTAGACAAGCTGCTCGCACACTGTCACCGCCGCCGCTACACCGCAA

F – ggatccactagttctagaGGTTTACTGGCACACTTCCTGATCT

R – ctatagggcgaattggagctcTTGCGGTGTAGCGGCGGCGGTGA

**Pant72**

ACAAAAGATGGCTTCCGATGAAGGGAATCGCTTGATTTACTGTATGAATAAACAGTATCTTGAGCGCTCCCCCCTTCGTGGCCATCCCTGTTGGCCATCCCAAGGCAGCTCGTCTCTCGAGCTGCCTTTTTCTTTGTCTCAATGGAAATCCCGGCTGCGGACGTCCAGGCCGGTGAGTAGAGGGGTCAGGTCATGCAGGCGCCCGGCGATGACGTGGCGCACCTCACCGGAGG

F – ggatccactagttctagaACAAAAGATGGCTTCCGATGAAGGG

R – ctatagggcgaattggagctcCCTCCGGTGAGGTGCGCCACGTC

**Pant73**

ACGGAACGGTTGACGGAAGGTACGCGATGACGGAGCGGCATTGGCAGGGCGAGGTCTGGCTGGCGCGGGACCACGCGCTGTTCGCCGGGGTCAACGGCGATACCCGCGAGCACGCGCACTATGCCCACCAGTTGCTGCTGGCGGAGACGCCGCTGCGGGTGCGCGTCGCTGGCCAGGTACTGGAGGCCCGGCGCTTGCTGGTGCCCTCGCTGCAAGCCCATCGCGTCGACGCGTCGCAGCCGTTGCTGGCGCTGTACGCCGAGCCGCTGGCGTTCGACAGCGA

F – ggatccactagttctagaACGGAACGGTTGACGGAAGGTACG

R – ctatagggcgaattggagctcTCGCTGTCGAACGCCAGCGGCTCG

**Pant74**

ATGCCGCAACACAAACGCACTCGGAAAAATCGGTAACAGCGGAACCACTGCACAGACCAGAGAAAAAGCCGCCACCAACCAGGCCTGGCGGCGACACCGGAAGTACCCTCGGCGGCCGATTGCCGAGCCGATCCCCTACCTGCCCCCTGGGTTTTCCGACGAAAGACCTTGATTCGTGGGAGGTAGGGTCGTCTCCGCTA

F – ggatccactagttctagaATGCCGCAACACAAACGCACTCGG

R – ctatagggcgaattggagctcTAGCGGAGACGACCCTACCTCCCA

**Pant75**

GCATCGCAGCACGCTTTCGTGGCTGCGCCCTCCCCGATCCGAGGCTTGCATGTTGGAGATAGTAGTCATCCGATAACCGCCACTTCGTCATCCCGCACGAGGTGTCGCCGGTTATCTGCCTTCGCCATGGCTCGCACGCGCCTTTTCGCGCCGTGCGGCCGCGTTGTCACACCGATGATTACAGGTTCTACCGACATGAACGTTTCCAAGCCGGAACAACGGACGCTTCACGCCTTGGCCCAGGGCGGCCATATCGCCTTCCTTCGCGAC

F – ggatccactagttctagaGCATCGCAGCACGCTTTCGTGGCT

R – ctatagggcgaattggagctcGTCGCGAAGGAAGGCGATATGGCC

**Pant78**

TGCTCTGGTCGGTGTGGTTGGGGGCGTTCTTCGCCGGCGCCTTCATCACCGGGTACCGGACCGGCGAATTCTTCTAACCGAACAGACCGAGGCGGAAGCCCCCTCCGGAGTTTCCGGCAGGGGGCTTTTTTGTGTGGGGTCTTACGATGAAGTTTGCGAGCCTGATTCTGATGCTTCTCTTTGCCACGGTGG

F – ggatccactagttctagaTGCTCTGGTCGGTGTGGTTGGGGG

R – ctatagggcgaattggagctcCCACCGTGGCAAAGAGAAGCATCA

**Pant81**

GCCGGGTTGCAACCGCCGCAACCGGCTTCCCGCTCCATCGGGATTTTGTCCAGGCGTGGTTCGGCTGCGTTCTGCGATGAAGGAGAATGCTGATGGTCATGCGATTGTTCGGGCTGCTACTGGCGGCCTTCCTGATGAGCGGTTGCAGCGTTCGGGTGCCGGGCGCCTACGTCGAACTGGATCCGTTCGACGATGGCTACTACGGCTACGATCGCGGCGGCCCGCCCCATTGTCCTCCGGGCCACGCCAAGAAAGGCTGGTGCTGAGCCCACTTCCTTTTCCCGAGTCTTCACGTCGTATGCGCGCCTCGCGGCGTCGCCGGTCGTCCGGCCGGTCGGCAGCGGGGCCGCAGGAGGCTGCCATGTGGCGCACATTCCTGA

F – ggatccactagttctagaGCCGGGTTGCAACCGCCGCAACC

R – ctatagggcgaattggagctcTCAGGAATGTGCGCCACATGGCAG

**Pant83**

TCAGTCAGTCAGGACCGTCATCATAGAACTCGGTCCGAGGCTTGTCAGCGGGCAAGAATGCCTGGCGCATCCGCCTTGGCGGTTGGCGGACACTTCCGAGGCCGCCGTCGGCATGGGCCTGCGTCCCGCGCGGATACCCTTGGTTTGCCGGGCAATACGACCAAGGGCGGACGCCGGCGGAGCGACGCCTCGTCATCGGGCCGCGCCATTCGAGCGCA

F – ggatccactagttctagaTCAGTCAGTCAGGACCGTCATCATA

R – ctatagggcgaattggagctcTGCGCTCGAATGGCGCGGCCCGA

**Pant84**

ATTCTGTACGAAGACCGACGTCCTGCGCCGTGGCGACCTGGCCTATCCTGACAGGCCCCGTCGCCCCGAGGACTCCATGCTCCGCGCCTCCTTCGTCCTGCTCGCCCTGTCCTGCGCCGCTCCTCTGCTCTCGCTGGCCGAACCGTTGCGCTTCGCAGGCGACGACGGCTGCCC

F – ggatccactagttctagaATTCTGTACGAAGACCGACGTCCTG

R – ctatagggcgaattggagctcGGGCAGCCGTCGTCGCCTG

**Pant85**

ATCCGCTGGTGCCTCGCGCCGGCGGAATGGGCCTGACCCATACTGATCCGTATTCCCGCGCCACAGGATGTGCCGCGCCGCCGGCACAACCCGCCGGCGGCGGTGCCCCGGGCAATGGACGCCTGGCCGGACTTCTTTCAATAACAATGGCTCCACAAGAGGAGGGCGTGATGTTCAAAGCCAGTCACGTCTTGCAGGGCGAGGACCAGACCCGGTCCGCAGCCGAATTCTTC

F – ggatccactagttctagaATCCGCTGGTGCCTCGCGCCGG

R – ctatagggcgaattggagctcGAAGAATTCGGCTGCGGACCGGGT

**Pant86**

TAAAGGACGATTTTCCCTGCCCGACAGGGGAGGGATCGTGAGGCGTGGGCCTGTTCGGACTCGTTTGAGGCCACCCACGTTTCCGGCTTGCTCCGGCAAGCGCTCACGGAGTGACGCCACAAGCGCCATTCTGCCAACCAAGGGCCCGCTACCCGTTGCTTGCACACGCAAGGGCCCGGATAAAAACAAGCAATAGGGGAGTCCCTATGAGTGTCAATACGCCCACGCTGATCACCTTCGTGATCTACATCGCGGCCATGGTCC

F – ggatccactagttctagaTAAAGGACGATTTTCCCTGCCCGAC

R – ctatagggcgaattggagctcGGACCATGGCCGCGATGTAGATCA

**Pant87**

CTGAGTTCCTTGTGTTTCGGGTCCGGAATGTCGATGCGCACACTTTTCTCCCTTCTCGTCGGTGTCGTCCTGGCGGGTTTCTCCGGCCTCGCGGCGCAGGCCGCGCCGGCGCCCTTCTACAAATGGCAGAGCAAGCTCGACGGTCAGGTCGCCTGCATGCAGACCTCGCCGGGAGACGGCTGGGTCAGGCTCGACGGCCCCTACCGCGATCTCCGCTGCCGCGAACCCCTGCGCTGACCCCATGCAGCGCAGCCGGCTGCTCGAGCGCAGCTGGCGCCTGCAAG

F – ggatccactagttctagaCTGAGTTCCTTGTGTTTCGGGTCC

R – ctatagggcgaattggagctcCTTGCAGGCGCCAGCTGCGCTC

**Pant88**

GACCTCAGTTGTAGTCGACTCCCGCAATGCCCACGTTCGCTCCACGAGAGCGGACAGCCTCTTCCGGTGTGCAGTCCTGGAACCTATGACGCCCCGTCGTCGCCTGCTGTGCGGTATCGCGCAGGGGCCGCGCCGTTGGCGAAATCCGCTTCCCTACCCCGACCGCACGAGCGAGTCCGCAACTGATCTATCTCAATAACAAAATGAGGTTGTTCCCATGTCGTCTGTCGGCGAACAGAAGGTTCAAGACGG

F – ggatccactagttctagaGACCTCAGTTGTAGTCGACTCCC

R – ctatagggcgaattggagctcCCGTCTTGAACCTTCTGTTCG

**Pant91**

GGTTTGTTTTGCCGATTTGGCAAGCAGCTCGTCGACTCCCGCAAAACAGACCGGGAAAACCGGCCCACAGTAGTGAGGTCGCGCTTTTCCAGCCATCGACCCGCCCGAGCATCGTGGCCGGGCGGGTCCGCCACGAGGACAGGACCATGCAGACATCCCCCGCTCCGGCCATCGTCGCCGGCGGCGCCTACCAGCCGGTGGTGCTCCACGCCGGCATCGCCTACGTCAGCGGCCAGTTGCCGCGCC

F – ggatccactagttctagaGGTTTGTTTTGCCGATTTGGC

R – ctatagggcgaattggagctcGGCGCGGCAACTGGCCGCTGAC

**Pant92**

GTCTGGCTAGGCTGTTGTGGTGAATTGGGCATGCCTTTTCGCTACATGCCTAACGATCTCAAGTAGGTATAGGTTTCTGGTAGAGGGGCATTGCTCCCGCTCTTTGCCAGGTTCGATTTTGGGCAGGTGCCTGATAGGCTGTCGCTCAACCATCATGAAAGGACTCTGCAGCTATGCCCGCCGTTATCGCTGAGAATGATGTGTCGATCTGGGACGACGAAACTGGAGCTGT

F – ggatccactagttctagaGTCTGGCTAGGCTGTTGTGGTGAAT

R – ctatagggcgaattggagctcACAGCTCCAGTTTCGTCGTCCCAG

**Pant93**

CGTCATGCGCCATGCCTGGGCTCTTTACCCCGTCCCTGGGGATGCGCCGCAGCCAGGGTTCTTGCCCGGTGCGGGGCTTGGCACGTTGTGTCGCTGAATGCGCAAGGAGTACGTCATGAGTGCCTTTCACGATCTCACCCTGCAGGGGTTGGATGGACAGGACCTGCCGCTTTCCCCGTTCAAGGGCAAGGTGCTGCTGG

F – ggatccactagttctagaCGTCATGCGCCATGCCTGGGCTC

R – ctatagggcgaattggagctcCCAGCAGCACCTTGCCCTTGAACG

**Pant94**

GTCATACAATGCCGGTTTCGGACTTTGAAGTCAGCCGGTCGCAGGGCGGGTTTTCGGAAAAAATCACGGCCATGAAAAATCCCACCCCGAGGGGTGGGATTTCCTTGCCGGCGCAGCGCCGGACCGGTGTGTCGCGGACGCGCCTTCAGACCAGGCTGGCCAGGGCCTCGCGGCTGAACGGCTTGATGTCGCCGATGCGCCCCTCGCGGACCTTCACCGCCCACTCCGGATCCACCAGCAGCGCG

F – ggatccactagttctagaGTCATACAATGCCGGTTTCGGACTT

R – ctatagggcgaattggagctcCGCGCTGCTGGTGGATCCGGAGT

**Pant96**

ATGGTGCGCCTGGTCGAGGCCCTGCAGTTCACCGAGGAACACGGCGAGGTCTGCCCGGCCGGCTGGCGCAAGGGCCAGAAAGGCATGAAGGCGAGCGCCGAGGGCGTGGCCTCGTACCTGGCCGAGAACGCCGAGGCACTGTGATCCCGATGCCGGGCGCGAAGTGTGCCCGGCCGACCTTTTCCCGGGGCGACCGCTCCTTCCAAGCCTTCCCCATGTGCTTTGGTGGCCCCGTTTTTATTCGCGCCGACGCTGATGTCGGCAGGAGTCCGACGAGATGCC

F – ggatccactagttctagaATGGTGCGCCTGGTCGAGGCCCT

R – ctatagggcgaattggagctcGGCATCTCGTCGGACTCCTGCCG

**Pant97**

AGCGCCGGCCTTCCGCCGGCGTTGTCGTTTCCATGGCGCCAGCGGCTGGCGCTTTTCGTTCCGGTCCGCCCTTTACGCGGGCGACCACCTGGAGGCTATATCCCCATGACATCCATCGTCGTGGCGGCGCTGTACAAGTTCGTGACTCTCGACGATTACGTGGCGCTGCGTGAACCCCTGCTGCAAACCCTGCTCGACAAC

F – ggatccactagttctagaAGCGCCGGCCTTCCGCCGGCGT

R – ctatagggcgaattggagctcGTTGTCGAGCAGGGTTTGCAGCAG

**Pant98**

TTTCCGTTCCAGCGGCGCCGATCGCGCCAGCCTTGCCCGCACCGCCGAGCCACGAGCCTGGCGGCCCATTCACGTCACGTACAACAACAAACAGGTGACGCATGATCCGTGAGTCCTCCCTCCGCCCAGCCGACCGCCCGCCGCCACGCGCCCCGCATGGCGCCCGCCGTGCGCCTGCCGCCTGAGCCGCACGGCCCCGTTTCGC

F – ggatccactagttctagaTTTCCGTTCCAGCGGCGCCGATC

R – ctatagggcgaattggagctcGCGAAACGGGGCCGTGCGGCTCA

**Pant101**

GTGACAGGACGCCGCCCCGCACAGAAAAAATCTGCTGCGACCACTTGGGAACTCGCCCGAGAGGCCATACTCTGTCGCGCAAATTCCCATGAGGTGGCTGATGCGCACCGACGCTCCCGTTCCAGAACATCCCGCTCCACCCAGCTCTCCCGCGTCCCCACAACGCATCCGGCTGAT

F – ggatccactagttctagaGTGACAGGACGCCGCCCCGCACA

R – ctatagggcgaattggagctcATCAGCCGGATGCGTTGTGGGGAC

**Pant102**

TTTGCGGCGAACGATGTGCATGAGGCGCTGCATGCTGCCGGAGGAACCCCAGCAAGCGCAGGGAAAGCGCTGTTGGCACGCTTCTGGCAAACCCTCGTGTGACAGTCGTTTCACTCCGTAGTTCCGCCGAGGTTCCCGTTCATGCTCCCAATGTCGAACAAGGTCGTCCATTTGCATC

F – ggatccactagttctagaTTTGCGGCGAACGATGTGCATGAG

R – ctatagggcgaattggagctcGATGCAAATGGACGACCTTGTTCGA

**Pant103**

GCACGGAGTTTGCTTATACCTCTTTCATCACAGTAAGAGGGGCCGTACGGAACATGACATTTTTATTACAAGGCCCCGCCAATCGGGAAAAGCGACTTGAGAAGCGACCTCAACAAGAGTGACCAACCCCGCGACATACGTCATTTTTTCAACTGCGCACCTACGCAGATGCGACATGCGTCATGCAATTTTGCGACAGCACGGTAAAGAATCCGTCGCTTCGGAACCTCAACTA

F – ggatccactagttctagaGCACGGAGTTTGCTTATACCTCTTT

R – ctatagggcgaattggagctcTAGTTGAGGTTCCGAAGCGACGGAT

**Pant104**

TACTGCAATCGTCACATCCAACCCGCCCGCTGCAGGCCGGCGCAGCCGCCCTGCTGGTCCTGGGCGTCGTCCTGCAGGCACTGATCCTGCTGGCGCCGCTGCCGACCATCGCCGACCAGCCGGCGATCGTCCTGCCCCTGCTAAGCCTGGTGCTGGCCACGGCCCTGCAACTGGCGGGCAGCCTGAAACCCGGCAAGCCGGTCTCGCGCAGCGCCAGCGCCCCGGCAGC

F – ggatccactagttctagaTACTGCAATCGTCACATCCAACCCG

R – ctatagggcgaattggagctcGCTGCCGGGGCGCTGGCGCTGC

**Pant108**

CTATTCGTCGCGGCGCTGGCTACGGCCGGCAGGACGCTGCGAGCGGCATGCCGCTGACCTGCTCCGTGGCGCTCCGGAGGAGGGCGGCAAGCCGCCTTCCTTCATTTCTACTCGTTGCCGAGCATCGCATCCAGATCGCGCATGGTCTGTTTCTTGGCCTGGACGCCCTGCAGTTCGACGAACATTGCCTGGACGGTCTGCAC

F – ggatccactagttctagaCTATTCGTCGCGGCGCTGGCTAC

R – ctatagggcgaattggagctcGTGCAGACCGTCCAGGCAATGTTC

**Pant109**

GCCATCTCATGGGTTCGGACGAGGCCTCGAGCAAGGGTTGTAACGGTTTTTGTCTGGCCAATGGGCTCTTGCGTAAAAAGGCTGCCGCCCTTCTTGCTTGGTTGCCGTTCTCGGATCCCGCGCAGCCCGGTGGGTGTGCCAAATTTCTCGCGGTTTGGATCGCGCCGATTGCCGCGGCCTACGAAGCCCGTGGTTCTTCTCCCCGAAACTTTTTCGTTCGGACTCCGAATATCGCGCTTCGCCCAGCGCCGCTAGTTTCCCGTTCCTGACAAAGC

F – ggatccactagttctagaGCCATCTCATGGGTTCGGACGAGG

R – ctatagggcgaattggagctcGCTTTGTCAGGAACGGGAAACTAGC

**Pant110**

ATTCCTTTTATTGGGTGGCGCGTGCCGCTTCCCTTGATCGGGTCAGGTTGCCGCTACTGTGGAAGAAGCGTCGAGGACTCGATAGATAGCGCCCGAGTGTTTCAACTTGTCTTCTGGATGACGTTTTCATCGGGGAAACCTCCCGTCGGTCAGTGAAGCGCAAGGGCTGGCGTGCGAGGGTGGAATCGGCCGCCGGCTCGCTTTCTGCGCGGCGGGCGCACGGCACGGGGAGTCGT

F – ggatccactagttctagaATTCCTTTTATTGGGTGGCGCGTGC

R – ctatagggcgaattggagctcACGACTCCCCGTGCCGTGCGCCC

**Pant112**

TTCTGGCCTTCCGGAACGTACAACCGGGGACGGAAGCAGTATCCGGTTCGGCGTCCAGCGCGATGCACTTTGCAGCAAAGGTCGACAAGAACAAAGGAGAAACCTCGTGGCAGATCTTCCCGATGGCAGTACTCCGGGACCTGAGGCGCAGTACCTTGCGTTTCTCGCCCAGGGCCGTTTCATGTTGCAGCGCAGCCGTTCCAGCGGTCGCCATGTGTTCTACCCGCGCCAGTTGGTGCCGGGTAGCGGCG

F – ggatccactagttctagaTTCTGGCCTTCCGGAACGTACAAC

R – ctatagggcgaattggagctcCGCCGCTACCCGGCACCAACTGGC

**Pant114**

TAAACACACATGAGGAGGTCGTCATGAGCGCTCTCATCAAGGAACGTCCCAGCGCCGATGCCGTCCTGGCCAAGGCCGTCCTGGCCGCGCGCGAGCAATTGGGGTGACGCAGCTCGAACTGGCCGGCATCGTCGGCGTCGATCGCAGCGCCATCAGCCGCTGGAAGACCCAGGGCTTGCGGGTGGACAGCAAGACCGGCGAG

F – ggatccactagttctagaTAAACACACATGAGGAGGTCGTCATG

R – ctatagggcgaattggagctcCTCGCCGGTCTTGCTGTCCACCC

**Pant116**

CTTGTCGGCTCGACGCTAAATTTCATTGCCAAATAATGGCTTAGCGACACAATCTAAAGCGTCGTATGAGATAGGGCGTTGCCGGGGCAGTGGATCGACTCCGGCATCGCTGCGATGTGACCGGCGGTTGAGCCGAACGTTCCATCGGGCTCGCCTTCTTTCACTTCCAACGGACCCCGACGTATGAAACTGCTTAAGACCTCGACGCTTCTTCTCTGCATGACCGCCTCCGGCTGCAGCTATTTCCAGACCCAGGACGCTTCCTCGCAGCCAGCCAAGGCT

F – ggatccactagttctagaCTTGTCGGCTCGACGCTAAATTTCA

R – ctatagggcgaattggagctcAGCCTTGGCTGGCTGCGAGGAAG

**Pant118**

AGTCTCGCGGCCGCGGGGGCCGGAGGAGACCGGGTGATCGTCATGGACGGACCCCGGGAACTGTACCGGTACAGTGTAGAGCGGCGCTTCGTCGCTTGAAAAGCACGGCACGGCGAGCTCCCGTGAGTCGTGTCGCCTGGTTGACATAAACGTATAGAAAAACGGCGCGGACCTGCCTCACCTCCCTGGGCCCGGTTTCCCTTCGGCTGTCGTGGGCTACCGGCGGGCGTTATCATGGCGCCCATGC

F – ggatccactagttctagaAGTCTCGCGGCCGCGGGGGCCG

R – ctatagggcgaattggagctcTGCATGGGCGCCATGATAACGCCC

**Pant119**

TTTTCTTTTTCTCTTCCTCTTCAGTGGGTTACATGCTTCGAAGAGGACTCGCCAATATCGGTCCTGAACCGCCCCCGGTTGGTCCGGCAGAGGGTGGAAGTTCCGACACCCATTGCATCGACCCCCCGGCGATTTTCTTTAGCCCTCCCGTGCAACAGGGCACGCCGGGCATGAAAAAAGGCCGATGGCTTGCGCCACCGGCCTCGTTCGAGAAAAACCCGGATTCAGTCCGGCAGCCAGGC

F – ggatccactagttctagaTTTTCTTTTTCTCTTCCTCTTCAGTGG

R – ctatagggcgaattggagctcAGCCTGGCTGCCGGACTGAATCC

**Pant122**

ACACCCATCGTCGGGCGCACGAGCTGCCGTAGCGAGCTCTCCACGTTGGCCAGCAGCCCACCTCTCCCTACTGGAAAGTCAGGTCAATAACACTGGTGCCACGTAAGGATGGGCGTTCCTACTGGCCCCTCATACGCCAGGCTGCTACGGCAGATCGTGTCGCTAACCTCAAGGGACACAACCCGCAAGAACGCGCGTCCCTGAAAAAGCGCCTGCTGCGCAAGTGGATGAGCAAATGAGTACATCCAACATCCAGGCATTGGCCAGCACTGGGC

F – ggatccactagttctagaACACCCATCGTCGGGCGCACGAG

R – ctatagggcgaattggagctcTGCCCAGTGCTGGCCAATGCCTG

**Pant124**

AGCGGAATCGTCGTGTAGGAGGCCGAATGCAGGGGCAAGCGCTTTGGTTACTTTCTCGGGGCCGGCCATCCTGCGAGGCCGAGAAAAAGACTTGGTAGCCGGGGTAGGGCGAATAACGGCACAGCCGTTATCCGCCAGCAGACTCAAACCCGCTCGGCCGCCCGGACCTCCAAGGAAGGATCACCGGCCCAATCCTCGGGATAAAGCCCA

F – ggatccactagttctagaAGCGGAATCGTCGTGTAGGAGGCC

R – ctatagggcgaattggagctcTGGGCTTTATCCCGAGGATTGGGC

**Pant126**

CCCGAAAGGTTGGGGAGTAGGTCTGCGCCGGTGCTTTCCGAGGGCGACGAATGGCATTCTCCAGCGCTGCCGCGGCCTACGGACGCTTCCTAGGACTCACGCACAGAAGACCCACACTTTATCCACAGGTTTCCCCCAGACTGTCACCTTGCAAACACCCCATTAGCGCATTATCTTGTATCCCCATCGTCGCCACCCCCTATATCTTGGGTTT

F – ggatccactagttctagaCCCGAAAGGTTGGGGAGTAGGTCT

R – ctatagggcgaattggagctcAAACCCAAGATATAGGGGGTGGCGA

**Pant127**

ATCAACAGCAGCGGACAACGGCCGGGGGCTCGCCGATGAAATCCATCTTCCTGCGCATCTATGGCGGCATGCTCCTGGCCCTGCTGCTGGTCAGCGGCCTGGGCTTGCTCGGCCTGCACTGGATCAACGAGGTGCGCGGCGAGCACTACCGCGAGGAACTGGCGCGCGGCACCTTCCGCCTGATGGCCGACAACCTGCAACCGATGACCGCCACCGAGCGCAAGCGCGCCCTGGCGCAGTGGAGCCGCTTGCTGGGGATTCCCCTGCAACTGCG

F – ggatccactagttctagaATCAACAGCAGCGGACAACGGCCG

R – ctatagggcgaattggagctcCGCAGTTGCAGGGGAATCCCCAG

**Pant128**

AATTATTGACGGCGAACTTTTGACTTGCAAAGAACTACGCTGGCCAGCGGCAAGAGTGAATTTCATCCAGCCAGGGCTATACCAATCGTGCAAACCCGGAAGGGCGCTGCGCAAAACGTCACATTTATTCGGGACGATACGCCAAGGCGGTTCCCTGTACTGAAGTACCAGAATCGCCAGATCGCGCGGCCTATTTCCCCTTGGAAAAATACGGATATTTTTTTCCGGGCCAGGTTTTCCTTCTTCCGCGCCTGCCAGATCTGGCAGTTGCG

F – ggatccactagttctagaAATTATTGACGGCGAACTTTTGACTTG

R – ctatagggcgaattggagctcCGCAACTGCCAGATCTGGCAGGC

**Pant129**

CCGTGTGGCTGACTCGACCAGTCGAACGCCGATCACCGAGTCAACCGTACAGGCCACCAGGCCTTCGGCGCCGGGGGAAGCCCGCCGGTGCCATGGATGACCGTTCACCGTACAAGGAAACCCCATGCGCAGCCTTCTTCTCTCCTCGCTGGCCCTGCTACCCGCCCTGGCCCTGGCGCAACCCGACGCCTCGAGCTTCCCTTCCTGCCTCGC

F – ggatccactagttctagaCCGTGTGGCTGACTCGACCAGTC

R – ctatagggcgaattggagctcGCGAGGCAGGAAGGGAAGCTCGA

**Pant130**

GTTGATATATAAGGATTTTATTTAGATAAAGAGCATCCTGGCACAGCCTCTGCAATGCCTGGGCAAGGTTCCGCCGCGGCGGTTCACGACAAAAAATCTCCCCAGCAAGGGGGGTAACGCTACCGGTGAGCGCGTTCCAGGGAACTGGCGAAGCGCCCCCGTGAGGACTCTGCCCATGACCAAACAACCCTTCTACAAGA

F – ggatccactagttctagaGTTGATATATAAGGATTTTATTTAGATAA

R – ctatagggcgaattggagctcTCTTGTAGAAGGGTTGTTTGGTCATG

**Pant133**

GGGTTTGACAGCCCGAAGAGACGGCGCAATGCGCCGAGCGGTGCATGCGAAAGCAGCGAATGCTTTTGCAGGCATCGGCTCGACCGCAGTTTTCCAACTGCGGGCCTTCATGGCGGATCGCGTGGGGAGACCCTCGTGGTCTGCCGGTTCCGTTTCCCGGTCTGTCAACCCTGCGCGATCCTGCCTCCATCGATTGACAGCGAAGGCGGCAGGTCATGTGACTCTCAGAAACGGAGTACCCAGCATGACGAAGGATGAAAAGGAAAAGACCCACGTCGACGCAATCATCG

F – ggatccactagttctagaGGGTTTGACAGCCCGAAGAGACGG

R – ctatagggcgaattggagctcCGATGATTGCGTCGACGTGGGTCT

**Pant137**

GGTGTCTTCCACAGGAGCGAAGTTGTGAACTGTGGCGCGCCCGTGGGCCTGCGACCCGGCGGACGAACGGAGGTCCGCGGCGCCTGGCGCGCGCTGGCGTTCGAAGCGAGGGTGGCGGGCGGCGTTCGCCGTGTTATCCGATGTTCCAGGCTTGAAGGAGATCTCTCGATGTCGGCAAGCGCGAAGCTCTCCAGGATGGTTTGCTTGCT

F – ggatccactagttctagaGGTGTCTTCCACAGGAGCGAAGTT

R – ctatagggcgaattggagctcAGCAAGCAAACCATCCTGGAGAGCT

**Pant139**

GAACGCTTGGCGCGTGCCGCTGCCGGTTGGCGCCTTCTGCGCAGGCGTGTTGGTCGGCTCCAGTGGGCCTCCGTGGCGCATGTTTCGCAGGGCGAAACGCTCTTCCGGAAAGTTCGCGGAGAGGCTGGACAATAGAAAATTAGCTTGCTAATAATTCGCTCGCTCATGTATTTCGGGGCGTCCGCGATGGCCAAGCCTTCCTTCTTTTCCTCCGACCTGGTGCAGGTGCTG

F – ggatccactagttctagaGAACGCTTGGCGCGTGCCGCTGC

R – ctatagggcgaattggagctcCAGCACCTGCACCAGGTCGGAGG

**Pant141**

CAAACTACCAAGATTCCCAGACCGGCGCCAGTGTTCCGAAGTTCCGCAGGGTGCGGAACAGAGCGGTTGGCTCCGGTGCCAGTGAAGCGAAGGTAGCGCAACCGTTCGTGTCTGTATGTAAAAAATTTTCTGACCGGTTGGTCTGTTTCGGACGCGGGATTTTTCCATTCGCCGTCAGGCGACCTGGCTGAGGCTGCCGATGCAGCGTTGCC

F – ggatccactagttctagaCAAACTACCAAGATTCCCAGACCGG

R – ctatagggcgaattggagctcGGCAACGCTGCATCGGCAGCCTC

**Pant142**

AAGATGTCGCACGATAGCGGAACCCCTGGCAGCGACCGTGGTCTACCAGCGCCAAACCTGCTGCTCTCGAGGGCTTCCCTCCAGGAGCCGTGATGCATGAAAGAACAATATTCTCCGGCCTGATCGCCAGCCCTTGTCCGGCTTGGCAGTGTGACCCATGCGCTTGCTGATTCTCGAACGTGACCACGCTCTCTATGCCGCGCTGCTGATGGCGGCCGACCCCAGCCTGAAGGTGGTGGC

F – ggatccactagttctagaAAGATGTCGCACGATAGCGGAACC

R – ctatagggcgaattggagctcGCCACCACCTTCAGGCTGGGGTC

**Pant147**

AAGGGCGTACAAACCTGAAGACTGAGTTCCCGATGCAGAAGGTCAAGCTGACAACGGAGAGACCAACCTGAAGAGTTCTGGAGTGCCTACTTCCGGCCGTAAGGCTCCGGCGATCGGTTGAATCCAGAGTCGCTAGCGGTCTCTGACCAAGGGCAGCAACCGGACCAGGCTGTGTAAAAACGTGGCTA

F – ggatccactagttctagaAAGGGCGTACAAACCTGAAGACTGA

R – ctatagggcgaattggagctcTAGCCACGTTTTTACACAGCCTGGT

**Pant148**

TCTTCAAACGTCTTTGCTGTGGTGCCTACGAAATTAGGGGCGATTCAGCCCCATGTGGAGCATTGCAACCTCGAGATTTAGACAGCTCCTAAGCACCACCTCTTAGCTCGCATTTTCTTGAATCGCTCCTGCCATTACTGAGTTCCACTGCGGGGCTAGAGGAACACACGTAAGGTGGAAAATAAACCAACACGCATATATG

F – ggatccactagttctagaTCTTCAAACGTCTTTGCTGTGGTGC

R – ctatagggcgaattggagctcCATATATGCGTGTTGGTTTATTTTCCA

**Pant150**

GCAGGCCAAACTAGCCTAGCCTACGGAAAATCCTACCGAAACTTGGGTCAATCTCTCGGGATGGCGATTGCTGAATAATCAGCTAGGCGCTAGTCTTCCCGAAAGAAAAGGACTCCGAGCAAACAGTGAAAGAAAAATTGATTATTATGAACGCGCACACCACTTCATTGCAAGCTTGCGCGCTTTTATTGAAAAAATAGCTTATTAATAAGTGCCTTCGAACACTTAGGCGCACAACTTTAGAGCCAACTGATTTAAGGGCGTGAAATGTCAATAGATATTCTGGTAACTACCGACAG

F – ggatccactagttctagaGCAGGCCAAACTAGCCTAGCCTAC

R – ctatagggcgaattggagctcCTGTCGGTAGTTACCAGAATATCTAT

**Pant151**

ATTTCCCGTCTAGCTTAGCTGGACCAAGACCAGAGTGGAGGACCAAACCAATTCGGCGTCCGCTTCTGGCCGAAATCGGCCATCGAACGCCCACTCCACAAGCAATTTCCCGCTCCCACGAATCCAGATCGTGCCTACACACCGTCACGACGCAGTTACAGCATTAGGAAAACCCACACGCGCTGGTGGGGCATGC

F – ggatccactagttctagaATTTCCCGTCTAGCTTAGCTGGACC

R – ctatagggcgaattggagctcGCATGCCCCACCAGCGCGTGTGG

**Pant152**

CTGTCAAACAGGCTGGCCGCGTCGTCACCAGTATAGCGGCCGTGTTTATCCCGACTTTAGGGTCTGTTCCGTGAAGGCCTCCGGCGCCGCGACGAGGAGAGACGCTTGGCGATGTTGTTCGATGTCCGATCCTGCTGAAGTCCCCACAGCCAGCCCCTTGCTGGAAACCGTCGGGCTCGCCTGCGAGCGCGACTGGCGCATGC

F – ggatccactagttctagaCTGTCAAACAGGCTGGCCGCGTC

R – ctatagggcgaattggagctcGCATGCGCCAGTCGCGCTCGCA

**Pant153**

CAATCGGTTGGGGAGGTGCGGCGGTGAAGGCGAAGTATCTGGGTCTGGCGATTCTCCTGGGCGGTCTCGGCGGGTGCGTGGTGCATCCGCCGCAGACCAGCGAACCGCCGGCCTCGCTGCCGCCGCAGAGCCAGCCGCAGCCCGGTCCGTCCAGCGCGCCCGGCGCCACCCCGCCGCGCATCACCCCGCAACCGCAGACTCCGGCGCAG

F – ggatccactagttctagaCAATCGGTTGGGGAGGTGCGGCG

R – ctatagggcgaattggagctcCTGCGCCGGAGTCTGCGGTTGCG

**Pant155**

CCATACGGTCAAGTAGGCGGCCAATCCAAGAGCGTACCTGCCTTACGGACCTGTGGGATTTTCCGCCGGGCCTGCGCAAAGCCTCGCCCCGCCCCCTTCCGCCCAGCGCCGCCAGGCGCTACCGGGCGATTCACGGTAACAATCGCCGCACCAGGATGCGGCCCATGGGAGAACACGATGCGCCCCGAGGAACGCCGCCGCCACATTCTCGAATTGCTGCGCCAGCGCGAG

F – ggatccactagttctagaCCATACGGTCAAGTAGGCGGCCAA

R – ctatagggcgaattggagctcCTCGCGCTGGCGCAGCAATTCGA

**Pant156**

CTTGTCGGACCATGGCCTCCCTCGCGCACTCCCATGCAGGCCATGGACATTCAAGGCCTCCGGTTCAACTCCTGCCGGAGGTCTTTTTTTCTTCTTGGAACGCCCTGCATGCATGACCCTCGCCTGGCCTTCTCGTCCGGGGTGCGTCGCCTCCGCGCCAGACCCTACAATGACC

F – ggatccactagttctagaCTTGTCGGACCATGGCCTCCCTC

R – ctatagggcgaattggagctcGGTCATTGTAGGGTCTGGCGCGGA

**Pant168**

GCAGTACGGGTATTGACGCCGATCAAGCGGGACAAGATGTTAATGAATTTCGCATGAATTCATTCGCCCCCCTCGCGGCGGCATGCCCGCTCGATCCGCTCGGTTTCGCGCGGCTGAACATCGGCCATCGTCGCCGGAAAGCAGCGTCACCAGTGGTCCCGACAGGACAGGAAGGAAATGAACCTGATCGCGCACGTGGAAATACCGGTGTCCGACCTGGAGAGGGCGATGCGTTTCTACGCCTCGGT

F – ggatccactagttctagaGCAGTACGGGTATTGACGCCGATC

R – ctatagggcgaattggagctcACCGAGGCGTAGAAACGCATCGCC

**Pant170**

AATCTGCACAGGAGACGGAAGGACTCCCCATGCCCGGAACCGCCACGTAAACTGGCGGTTTTCCCGCGAGGCACACGCCATGTTGCTCAAGGCAGTACGTAACGGACTGGGTCAGGCGATTATCCTCGCCGACTTCGTCAGCCGCCCCCGCAAGCTGCAGCGCAGCGCCGAG

F – ggatccactagttctagaAATCTGCACAGGAGACGGAAGGACT

R – ctatagggcgaattggagctcCCTCGGCGCTGCGCTGCAGCTTG

**Pant171**

ATCCAGGACGAGAGAGCATGAGGCTCTACCGAATCCATGTCCGGGACACCTAGGGAAGCAGTACCGGACCTGGAGACCGGCGAGATGCCGGTCGATCTGCCGGGGACAGGATGCAAACGGCAGTGCCCTGGGGCAAACCACACCTGAACACTGGAGAAATAACAATGAAAAAGCACCTGTTCCTTGCAACTGCTGTTCTGGCTGC

F – ggatccactagttctagaATCCAGGACGAGAGAGCATGAGGC

R – ctatagggcgaattggagctcGCAGCCAGAACAGCAGTTGCAAGG

**Pant172**

ACATGTCGATGAAATGGACCGAGCAGCGCTTGCGCAAGGCTCTCAAGCAGATGGCGAACAATCATGAATCGGCTGCGGTCGAGGTCATGCGCGCCGTCGAGCGGGCGAACGATCCGAAGCTGGCGCAGCGCCTGCTCGAGGTGATCGAGCAGATGCACCAGGATGCCGATGCGCTGCGCTCCATCGACGACGAAATCGCCAGCGGCGTGATCCGTTGCCAATGAGGCCGTAGACGCTCCGCAGTTCAGGACTTCCCGGCTGAAAC

F – ggatccactagttctagaACATGTCGATGAAATGGACCGAGCA

R – ctatagggcgaattggagctcGGTTTCAGCCGGGAAGTCCT

**Pant174**

CTGTCCCGGCCCTCACAAGCCGCCGCCAGGCGTTATGGGCAAGCGTGTTGCCGCTCCATCACGGTCCCTGATCGTCGGCCTGGAGGAATGCCGGACGAAAAAAAACCGGAGCCGCGAGGGACTCCGGTTTTCCTTCTGGCATGGCGCCGGAAACGGTCAGACCGGGAACAGTTCGCCCAGCTTCATCGCCAGCATCATGTCGCCTTCGGCGCGCAGCTTGCCGGCCATGAAGGCC

F – ggatccactagttctagaCTGTCCCGGCCCTCACAAGCCGC

R – ctatagggcgaattggagctcGGCCTTCATGGCCGGCAAGCTGC

**Pant175**

GCTCCCAGGCCCGCCCTGGCGATCAAGGACGATGACTTCGAGGAAACAGGGATATGTTTCGACTCGCCGCGCCCCATCCGCGCCGGTTCGCGTGCGCCCCACGTCCCCTCTTCGCCGCTTTCCGCCTGCTGTCGTCAGGCGGCGCTTCCGTTCGCGCCCGGCGCGGCCTGGTCCATCCTTTCGCCGCCCTGGCGGCCCAAC

F – ggatccactagttctagaGCTCCCAGGCCCGCCCTGGCGA

R – ctatagggcgaattggagctcGTTGGGCCGCCAGGGCGGCGAA

**Pant177**

AATTATTGAATTGAAACGGAGGAGGACTTCCGGGGCAGGGCGCACAACCGCTTCGTGGGATGTGCGCCAGGGCGGCTATCCGCCCTCCGCGATGGCGCTGTCGGCGATGGCAGGCTCAGCCAGTCTTTTTCTTCTTCGGCAGCAGGCGCTTGACCAGCAGCACCCCCGCCAGCACCACGGCGCCGGCGACGATGCCGAACAGGAAGTTCAGC

F – ggatccactagttctagaAATTATTGAATTGAAACGGAGGAGGAC

R – ctatagggcgaattggagctcGCTGAACTTCCTGTTCGGCATCGTC

**Pant178**

GATCCACGACAAGGGCCAGCGTCCCGGCATGGCGCCGCGCGGTACCCGTCGTTCGATGGGCAAGCGCTGAGGCCTTTCCCGCGCCTGGCGTTTTGTGGGCCAGTTCCCGCCACGAAGGATCAAAGCGGGCAGCCTTAACAGTTTTGCCCTTTCGCGGCCGTAGGCCGCAGCCCCATCCCTTATGCTCGCCGCCCCTGGGCGAGCCGCGTCGATGCAGCGCGCCCGTTTGCCGGAATCCGCCTGCTGCATGTCGCGTCACCTTCCATCGTCTTTC

F – ggatccactagttctagaGATCCACGACAAGGGCCAGCGTC

R – ctatagggcgaattggagctcGAAAGACGATGGAAGGTGACGCGA

**Pant179**

CTCCCCCTGGACCACCGGCCGATGCCGTGGTCATTCCCTGCGAAACGCTGTGCGCCACCTCCCGCCCGTGCCGGCGGCAAGGTGCGCGACGTCCCGCGCTTCGATTACGCCGGATCGTCCAGCCCGCCCCGGGCCGATCCGGCTTTTTTTTGCCCGCCGCCGCGATTGCGAGGCGGCCGCCGTCGGTTGGATTTCCTGAATCTTCCCGCGCCTCGCCGGTCGACCGAACAAGGG

F – ggatccactagttctagaCTCCCCCTGGACCACCGGCCGAT

R – ctatagggcgaattggagctcCCCTTGTTCGGTCGACCGGCGAG

**Pant182**

GTCCCCTGTCAAATCTGGTTACAACTGGGTTTCAGGCGAAACATTCGGTCATGGCAATTCGGCATTAGTTGAAACTTTGGAGACGCTCCGAAGCGGGCAACTTTTGCCCGGAAAAAGCTTCACGGCAATTTCTCCGGCCTGTCATCCCGATGTCTTCTTTCCGGTATGGATGCCAGTCGATTCGAACTGGCGGAGATTCGCACCATGCGAGAGTACCA

F – ggatccactagttctagaGTCCCCTGTCAAATCTGGTTACAAC

R – ctatagggcgaattggagctcTTGGTACTCTCGCATGGTGCGAAT

**Pant183**

AACTAGGAATCGAGGAGGTGCTCTCGTACGATCGTATGAGTCTTGTGAAGGACAGGCCTGGTCGGGACTGCCCGCCCTGTCCGAGCGAGCTGGCAGGCCCGAGCGGCGCCGCTGAACCGGAGGCTCAGGCGTCGCGCAGTTCGAAGGCGGGAAACAGCTCTTCCAGTTCTCGCCATAGCTGCGCAGCGCCCTGCGGCGC

F – ggatccactagttctagaAACTAGGAATCGAGGAGGTGCTCTC

R – ctatagggcgaattggagctcGCGCCGCAGGGCGCTGCGCAGCT

**Pant184**

GTTTCCGTTTCTCTTGGTGCTCGCCGTGGAAGTAAAGGTCAATTTTCTCGATAACCTTCGACTGGAAGCCAGGTTCGATGATTTCACGGTGATCGCCGACCAGCCGATCCGCTACAAGGGCGACGGCTCCGCGCCGGGGCCGTTCGACTACTTCCTGGCGTCGTCGGCGCTGTGTGCCGCCTACTTCGTCAAGCTGTACTGCCAGACGCGCAACATCCCCACCGACAACATACGCCTGTCGCAGAACAACATCGTCGACCCGGAGAACCGCTACCGGCAGAT

F – ggatccactagttctagaGTTTCCGTTTCTCTTGGTGCTCGC

R – ctatagggcgaattggagctcATCTGCCGGTAGCGGTTCTCCGG

**Pant185**

ACAACATCGTCGACCCGGAGAACCGCTACCGGCAGATCTTCAAGATCCAGGTGGAACTGCCGGCCGACATCTCGGAGAAGGACCGCCAGGGCATCCTCCGCTCCATCGACCGTTGCACGGTGAAGAAGGTGGTGCAGACCGGACCCGAGTTCGTCATCGAGGAGGTCGACAACCTCGATGCCGACGCCCAGGCACTGCTGATGCCCGGCACCGATGCCGCCGGCTGCACGCGCATCCCGGGCAAGGACCTGCCGCTGGAGCAGACCATCGCCAACCTGTCGGCC

F – ggatccactagttctagaACAACATCGTCGACCCGGAGAACC

R – ctatagggcgaattggagctcGGCCGACAGGTTGGCGATGGTCT

**Pant188**

GCTTTTCTTATCGTTGTATTGAGGCTTTTCCAGGAGATTATCCAGACGCAAACGTTTGCGTTAGGTGAATATGCGTCGATCTGACAGATTCTGTCAATCGCCACGAGCAACTGTCGCGAACCGGTCGGGATCGGTTGGATTCTAGTTCGCTACGGCTGGGAAACGCAGGATATTTTGCCTGGCTGGCAGATCTCCCGCCGCCCACTCAGCGCAACCGGTGCTTGTGCAACAGGCGGTAGAAGGTCGGCCGCGACACCCCGAGCA

F – ggatccactagttctagaGCTTTTCTTATCGTTGTATTGAGGCT

R – ctatagggcgaattggagctcTGCTCGGGGTGTCGCGGCCGAC

**Pant189**

CGAAAATGCCGTGGTCGCGGGATAGACATTGGTTTGGCTGTCCTTTTGTTGAAAAACGACGTCGCAATCAGAACAGTCCGTAGGCACTGGCGCCGGAGCGCTCCGCGTTCCTTGCCGCTTGCTGTCTTCCGCCCGCATCGGCGGCGCCATGCGGGGGGATGCCGGCGAACCCGGAGAGCTCCCGGAAAGCTTCCCGCGAACTTTTTGCAATCCGGCTGAAAGCCCCGGAATACAAGGGTTGCGGCGCTTTCGGAGGGTTACGTTTTCTGTA

F – ggatccactagttctagaCGAAAATGCCGTGGTCGCGGGATA

R – ctatagggcgaattggagctcTACAGAAAACGTAACCCTCCGAAAG

**Pant190**

ACGAGGTATCGGCGGATAACGCCTGTGGCGTTATCCGCCCTACGCAATGGGGCGGGCGAACTCCGGATGGTTCCCTCTCCCTTCAGGGAGAGGGTCTGGGGAGAGGTTGGCCGCCCCGGCTACTGCGCCACCCAGCCGCCATCCATGTTCCAGGCCGCGCCGCGCACCTGGTCGGCGGCTTCGCTGCAAAGGAACAGCGTCAGTCCG

F – ggatccactagttctagaACGAGGTATCGGCGGATAACGCCT

R – ctatagggcgaattggagctcCGGACTGACGCTGTTCCTTTGCAG

**Pant191**

TTTATTGCTGTTGTAGGGTTGGCACAGCTTAGCCTTGTTCAATTTTTCGTACAACGGAGCCTGTAAAATATCTGCTGCACCAGGTTGAACACCTTGCCTGGCCTGGCTTTGGCCGCTTTCGCGATGCGCTGGAAGGCCCTGTCTACGGCCATTTCGCCGGCCGCGAGGGCGATGAGAGGGCTATTGACAACAAATTGGCTTTCCGATTGACTCTGGCTCCAGCCCCCGCATGATTGAAATTTTTAACAAAAAAGGTGTTGCATCATGTCGGTACCC

F – ggatccactagttctagaTTTATTGCTGTTGTAGGGTTGG

R – ctatagggcgaattggagctcGGGTACCGACATGATGCAACACCT

**Pant192**

CCCGTTCTTCCGTCGAGGTTCCCACCCTTGCTGAAGGTAGACGGCGGCAAGCCTGCGGTTTCCGCCCGTCCGCCTTCCTTTGCTGTGCATTAGAAAAAGAAGAAATCGAGGCAAGCATACCCATGTCGAATCCTCCCCCCACCGCAGAACGAACCCAGCTTCGCAAGTCCCTCAAGCTGTGGCAGAT

F – ggatccactagttctagaCCCGTTCTTCCGTCGAGGTTCCC

R – ctatagggcgaattggagctcATCTGCCACAGCTTGAGGGACTTG

**Pant193**

TTGGTAATCTTCACGAAATTTTCACGGTGACGGGTCAATCATAGACCCATGGCTAACGGACTAGCCCCCCATCTGAAGCCCGCCTCATGGCGGGCTTTGGCTATCTGCAGACAGCGAATTTCGCGTTTTCCCTCCTTTTCACAGGGATTTCCGGCCTCCCGGCTGGCAATGCGCCTGCGACGACATGTCCGAATCCAGACAGAATTGGACGCCCCCAAAAAGCGCAGACACGGCTAGAATCCACGCCGTCCCGATCCGGTTCGATGGCCG

F – ggatccactagttctagaTTGGTAATCTTCACGAAATTTTCACG

R – ctatagggcgaattggagctcCGGCCATCGAACCGGATCGGGAC

**Pant196**

TCATTACTCGGGCACCTGTATTACGAAAACGGTCAATTGAAAGAACACTTCCTGCTGACCCACAAAGGTTCGCTAGTCAATCTGGATGGCGAGCTTTCCGATGGAGATGAAGTCGAAGTCATGCTCGCGACATCTGGCGGTTCTGGCGTTGAAGCGTTGAGCGACGAAGAAGTTCAACGCTACGTTCGCCACATCACGTTGCCAGGC

F – ggatccactagttctagaTCATTACTCGGGCACCTGTATTACG

R – ctatagggcgaattggagctcGCCTGGCAACGTGATGTGGCGAAC

**Pant200**

GAAGCCGTGGTTCGAGTTGTTGACTTGTCGCAAGACGGATTCTCCGAACAGCATCGGCGCAGCGCCCACTTGCAACGAGAGTGCGCACGCCGCCGTTGGCACTCGTCTGCGCACCGGACTTCTGAGCGGCGGCGGAAAAAGCTTGCTGCGCAAGGAGAAGCGCCTGCGGGTGAACATGGAAATTGCCCGGGAAAAGTCGACGGT

F – ggatccactagttctagaGAAGCCGTGGTTCGAGTTGTTGACT

R – ctatagggcgaattggagctcACCGTCGACTTTTCCCGGGCAATTT

**Pant201**

CGTCATCCGTGTAATGCATGGATGGTGCCATGCAGTTAACTTCCAAGTTGGAGTATGCACTTCCCTGTTATTCAACGAATAATTACCGTCGACTTTTCCCGGGCAATTTCCATGTTCACCCGCAGGCGCTTCTCCTTGCGCAGCAAGCTTTTTCCGCCGCCGCTCAGAAGTCCGGTGCGCAGACGAGTGCCAACGGCGGCGTGCGCACTCTCGTTGCAAGTGG

F – ggatccactagttctagaCGTCATCCGTGTAATGCATGGATGG

R – ctatagggcgaattggagctcCCACTTGCAACGAGAGTGCGCACG

**Pant202**

GCATGAGGCGCGGGATGCAGAATGCAAGAAGTCGTGCAGAACGCTCATGTCGGAGTGAGATCATGACGGGCGAAAAGCCCGTTCGGCGCTCCTTATGGGCATTCGAGAGAACTTTTCGCCCATATGGTTATATGGATTTTCTCGATATTTCCTCTAGGAACCTCGCCGGCTCGTTGGCGAAGAAGCCGATCTTTTTTGCCGGGTTT

F – ggatccactagttctagaGCATGAGGCGCGGGATGCAGAAT

R – ctatagggcgaattggagctcAAACCCGGCAAAAAAGATCGGCTTC

**Pant205**

AATGACGTCTAAACTTTAGTGATGTTGAACATGAAGTGCTGGTAAAACAGAATGACTCTTACAAGTCTGCTTGTGTTTTTTGGGCGGACTAGCCTGCTCGACGGAACTTATAGATCAGTCGATCTGACCAGCAAAAAACCATCCTATATGGGATCACCCAGCGCCCCACCTAGCTGCTTTCATGCAGGGCATAAACATGAAGCTCAAAAGCATTAGCATTTTTGCCATACTCACCCTTTACTACAATATCACTCTTTGTGT

F – ggatccactagttctagaAATGACGTCTAAACTTTAGTGATGTTG

R – ctatagggcgaattggagctcACACAAAGAGTGATATTGTAGTAAAGG

**Pant206**

AGTAGATGGTGAAATGCCTTGATCTTCACCTTTAAGAGAAAAGTCCTTAAGGACTTAACCTTGCTCCACTACCGGATAGTCACTGGCTGTTAGCTGGGTCGAGACGAGCTAGCACAAACTGAGAGATCGACAAGAATGATAAATAAGAGAAATGCTATTTTCCTCTCAATGATTCTCATCTTTTTTAGCATCGCCTGCAT

F – ggatccactagttctagaAGTAGATGGTGAAATGCCTTGATCTT

R – ctatagggcgaattggagctcATGCAGGCGATGCTAAAAAAGATGAG

**Pant211**

ATTAACCATTGTTCTGCTTGGCTCAGATCATGAGTCCTCTTCATAAACCCTTGCTCCTTGCAAGGTTATCGAGGAAGTAGAGCGGTCGCTGCGTGCGGCCAGTCGACCATTCGCAACGAGGAAGCGTTCGCGTGAAATCCCATCTTCTCCGCCCGGCCCTGCGCCCCGTGGCCGGCGGCCTATTGTCCGCCTCCCTGCTGTGCAACGCGGTGCACGCCGCCGAGG

F – ggatccactagttctagaATTAACCATTGTTCTGCTTGGCTCA

R – ctatagggcgaattggagctcCCTCGGCGGCGTGCACCGCGTT

**Pant214**

GTCTCGCGACCTGGGCAATCGCCTGAGGCGGCCTGTCCGCCACTGCCCGCGCGGGATTCGACAGACCACAACGACAAGAAGGCGGAGCCAGGCTCGCGCGATTTTTTCCAGGTCGCAAAGATAGCGCTGTCTCACGCTTGAGGTGTTCCATGCATCCACCCCTCCAAGCCCTGGTCATCATGGGTGTCGCCGGTTGCGGCAAGTCCAGCGTCAGCCAGGCCCTTTGCCAGCGCAGCGG

F – ggatccactagttctagaGTCTCGCGACCTGGGCAATCGCC

R – ctatagggcgaattggagctcCCGCTGCGCTGGCAAAGGGCCTG

**Pant215**

TGTAGTTCGTCGCCAGTGACGGACCAGAGGGATCGCCAAGGACAGGTTCTGCGCCGTTTTCCCTGTCATGCCTGTGATCCAGCAAGGGACTGCGATGATCAGGGCATCCCCCCGATGCATTGCTCCCCCACCATTGCATCGGAGTCAGCCGCCATGGCCGAGAGTACGCAGCACAAGCTGGACCGGGTGCGCCCGCCGCGCGTGCAGATCA

F – ggatccactagttctagaTGTAGTTCGTCGCCAGTGACGGAC

R – ctatagggcgaattggagctcTGATCTGCACGCGCGGCGGGCG

**Pant222**

AAGCTTACCCAATCGAAAGGCGCCGCAGCGTTCGTTCTCAGCGAAAACGGTTTTCGCCTGGACGCGCTACCGGGAAGGCTGTCGGGGCTTTTACCCTGTAGCCGCGACTCGTCGCTCCGTGGCCGACCGCACCTGCCCGCCCCCGTAGGCGGCCACGGATTTCCTTGCGCCTTCTAGACTGGAAGCGAACCCGCCGCATGAACCCCGAAC

F – ggatccactagttctagaAAGCTTACCCAATCGAAAGGCGCC

R – ctatagggcgaattggagctcGTTCGGGGTTCATGCGGCGGGTT

**Pant227**

GTAGGCGGGCCGAAGGGCGGTTCCTGGCCGGGCATGGCTTCGACCTGCCGGTGGGCGCCGCGACCCGAACCTCGGCCACGCCATTCCACCCATGCCGCCCGGGCACCGGCGTGCCCGTGGCAGCCCAGGCTACGGGCGCCGCGTAGCCAGCCAAGCGGGAGTCGACATGGCCTCGTGGACCTTGGTGGACGCCAGTGCGAT

F – ggatccactagttctagaGTAGGCGGGCCGAAGGGCGGTTC

R – ctatagggcgaattggagctcATCGCACTGGCGTCCACCAAGGT

**Pant232**

CTTTAGGGAAACTCTGAAAAATCACACCCGAAATCAGCGGCAATCCGTGCGTGGCTGATCCCGGCCCCGGGGTTTTGCCGGCCTTTTGCGGATAGGTGTAGCATAGCCGGCAGAACTCTTCAGGCGGCTCCATTGGAGCACGCATTTTCTTATGTGACAAAGGCTTATGGAGTCTTTTTATGAGTGAAGTCAAGCATTCGCGCCTCATCATCCTGGGCTCCGGTCCGGCCGGCTATACCGCCGCCGTGTACGCCGCACGCGCCAATCTCAAGC

F – ggatccactagttctagaCTTTAGGGAAACTCTGAAAAAT

R – ctatagggcgaattggagctcGCTTGAGATTGGCGCGTGCGGCG

**Pant234**

TTTCGATTCAACCCGATGTTGAGCGAAGGCATCGCGTCCACCAACGCGTATCGAGACTCTGTGCGCGCCCAGCAAAAAAGAGAGTTACCGTAGATGTCCATCCGCTCGAAGATCACCTACACCTTCACCGACGAAGCACCCGCGCTCGCCACCTACTCGCTCCTCCCCATCGTCAAGGCCTTTGCCGCTTCCG

F – ggatccactagttctagaTTTCGATTCAACCCGATGTTGAGCG

R – ctatagggcgaattggagctcCGGAAGCGGCAAAGGCCTTGACGA

**Pant235**

GGCCTCCACCGTGTTGATCTGCCCACAAGGCGGCTTGACGAGATTGCGACTGGAACGAAGTTGTCCTGAGGGACGGGGAAGCGTTTACGTATCAGCCAGATCGAAAGACTCGACAGCGAGTCGCCCACCGGCAACCACGAGCCGAACGGCACTTGATCCAGTCGAGAAGCACGGTCCGCGAACTGTTCGTCTCCACCCCCTCCTCGGCGTCATTCTTCGCCACGGTCATTCTCTCGCCATGCAGCGGTGAGCCACCCGCAAGACAACCTTCACCAGCA

F – ggatccactagttctagaGGCCTCCACCGTGTTGATCTGCC

R – ctatagggcgaattggagctcTGCTGGTGAAGGTTGTCTTGCGGG

**Pant238**

GAGTTGTCGCAGGACCAAGCGCTTTCGAACGCCGGCGAGCCCCATCGCAGAGGCTTTCCGTTACCCTATGGCGCGCCCCGGCCATGTCCTTGCGGCAAATTCAGCTTCAATTAAGAGCGCCCGGTTAATCTGCAAACCGTCGAAAGCAACACGATTGATTCCCACGGAGAGATTCCCATGAAGAAACTGACTG

F – ggatccactagttctagaGAGTTGTCGCAGGACCAAGCGCTT

R – ctatagggcgaattggagctcCAGTCAGTTTCTTCATGGGAATCTCT

**Pant239**

CGGCCCGCCATTGTCGCATTCGCGCAGCGAAGAACGGCCAGTCTGGTCCGGCCTGTAAACAAGTGCAAAATTTTACTTCATGGTCGGCTTGGCACTCCGAGCCGGCTCGTCCATGCTGGAGCACAACTTTCCCATGGATACCATTGCCATGAAGTTCCTGCTGCCCCTGCTGCTGTTATTCCCGCTTTCCGC

F – ggatccactagttctagaCGGCCCGCCATTGTCGCATTCGC

R – ctatagggcgaattggagctcGCGGAAAGCGGGAATAACAGCAGC

**Pant242**

CTTCGAGGACGCCCTGAGCCGCTCCTACCGCGAGTGGGAATACTGTGTGCAATACCGCGAGACCAGCTTCGATTTCGTCAGCCGGCTGATGGAGCAGGAAGGCATCTACTACTGGTTCCGCCACGAAAAGAAGCGCCACATCCTGGTGCTCTCCGATGCCTACGGCGCGCACCACAGCCCGGCCGGCTACACCAGCGTGCCCTATTACCCGCCGAGCCTCGGCCATCGCGAGCGCGACCACTTCTTCGACTGGCACATGGCGCGCGAGGTCCAGCCCGGCTCGCTGAGCC

F – ggatccactagttctagaCTTCGAGGACGCCCTGAGCCGCT

R – ctatagggcgaattggagctcGGCTCAGCGAGCCGGGCTGGAC

**Pant245**

ATTCCATGCGCCTGATGATCTCGTGACACTTTTCTCTCGACGCGAAAAACGACGCACGAAAGCTCCCCCAGAGATTAATCCGAGGGAGCCGTTCCGGGGAAATCTAACCGACGAAGATAACTACCAGATTACTGCGCTGTTACTCGATGATATAACGCTCAACCCTTTTCCTGTTCCGACGGGCCAGCGGCCTGGTCGAGGCGATGCCGACCCGGTAGTTGCCCCAGCACATGGCCGACCG

F – ggatccactagttctagaATTCCATGCGCCTGATGATCTCGTG

R – ctatagggcgaattggagctcCGGTCGGCCATGTGCTGGGGCAA

**Pant250**

TATAATTTCATCATAGAATATTGAGCTGCAAGCTAAAGAATCAATGATAGATTCAACATGAGGCGCTACTGAGTTATCCAGCACAACAGAAAGCTTTTTCACCCATGTTTAACCAATATCTCTCTGCTGCTTTTATTTTTGGTGAGAGGTATAGTTATTTGGCTCTCATATATTTTGTGCGAGTTTGCTGTGCGGCGGTAGTCGTTGCTTACTGGATTGAGTATTCCTCAGGTACGTCCCAATCCTTAAAGCGCGCCTCGCCAGTCT

F – ggatccactagttctagaTATAATTTCATCATAGAATATTGAGCTG

R – ctatagggcgaattggagctcAGACTGGCGAGGCGCGCTTTAAGG

**Pant257**

GATGAAAAAGACTGTTGTGCGCGGCGCGCTTCATGGCGAAGGGGCGGGCGCTTATTATTAGCAAGCTAATGATAACCGGAGTCAGCCTATGTCCAGAATCGAAGTCGCCGTGCTGGTGGGGACGTCCGTTCCCGAATCGCTGCGCAGCAAGGGATTGCTGGCCTGTTGGATCCTGATGGTGGACGGCGCCATGTGGGCCGGCCCCTTCACCAGCCGTGACGAGGCGGAAGCCTTGAAGGGAACCTGGGAACTGAGTTGGCCGCTGGAGGCGGCGATC

F – ggatccactagttctagaGATGAAAAAGACTGTTGTGCGCGGC

R – ctatagggcgaattggagctcGATCGCCGCCTCCAGCGGCCAAC

**Pant259**

AGAACATTGTTCGATCCAAGAAAGCTTCGAGTCATTTGCTAATAACCAAAGTGCAAGGAAAGCCCCTTCCATAGGCTCTGAATCGGGCGTTGCCGGATCGCTTCGTCATGCGCCTGCAATATCAACCTTATCGATAAGTACCATGCCGCGAAGTCGCTGCAGTTCTCGCTTTGGGCGGTGATACCGTCGAATCGTGAACGGATCGACAAAG

F – ggatccactagttctagaAGAACATTGTTCGATCCAAGAAAGCT

R – ctatagggcgaattggagctcCTTTGTCGATCCGTTCACGATTCGA

**Pant260**

ATGGACTACGGCAGAAGGGAAAAAAAGGCCATTACCAAGCGGGGGGAGGGTTGGTAATGGCCTGTACAAGGGACATTTGCCAGGCCTTGGGGATCTGGCGAAAAAGACGATATCAAGGGGGAATTTGATTAGCCAGCTAACAAAGTGGAATGAAGTTTTGCGGCAGGCGTAACAATTCTGT

F – ggatccactagttctagaATGGACTACGGCAGAAGGGAAAAAAA

R – ctatagggcgaattggagctcACAGAATTGTTACGCCTGCCGCAAA

**Pant263**

GCAAAAAAAATCCCGGCCGCGTGGAGCAGGCCGGGAAAGGATCCAGGGATGTACAGCGCCCACAGCTTATGTCGGCGGCGGTTTCGATGAAGCTGCCGGATCTGGTAGGCGCGATTTTCGCGTCGATGTGTGACCCGCTTCGCCGACTATCCTGGCAAGTGGGGTCGCGAACCAATAGGATTTGCGGGTTTTATTCAATCTGGGTGAATGCGTGCAGGAAATCTACTCTGAAGAACAGATGCGCAAGGCGCTGGGCCTTGCCGAAACGCGACCGA

F – ggatccactagttctagaGCAAAAAAAATCCCGGCCGCGTGG

R – ctatagggcgaattggagctcTCGGTCGCGTTTCGGCAAGGCCC

**Pant267**

CGGTCAAAACTGACCCCGTTCAGGACCTGCATTTTTCGGAGCGCTGTGCTCTGGCTGTGTATCCGCTCTGCGGATTTCCTTTACGTAATCTTCTAATTCAAAAGCAATCTCGCAAATCCCCGCCCCAGCTGCCTGGGTGGCCGTTTCGGCCGTACAGGGCATGAGCAGCCATGGGTCATGGGTCTTTGCGGATGTATACGAG

F – ggatccactagttctagaCGGTCAAAACTGACCCCGTTCAGG

R – ctatagggcgaattggagctcCTCGTATACATCCGCAAAGACCCAT

**Pant269**

AATTGGCCATACCTGCGTGACTATGAAACGTCTTTGCCGAATTTGCGTCGTTGTTCTTTTCGGTTGCTTCCTGGCGGCCTGCGATGACGGCTCCAAGGCACCGGAAAAGAAAGCCCCGGAAGCACCGCCGCAGGAGCAGCGCGCCCCTGCCAAGCCAGTGAAGCCTGAACGAGCGGAGCCAGCGCCTGCCGTGCCTGCCGCGCCGAAAACAGCTTCGAAGAAGGTCGCGCCAGCCGCGGAGCAGGTTGCCGAACCCAAGCCGCCGGCCAAGCCGAAGCCTGCGGCTGC

F – ggatccactagttctagaAATTGGCCATACCTGCGTGACTATG

R – ctatagggcgaattggagctcGCAGCCGCAGGCTTCGGCTTGGC

**Pant270**

GATAGCCGCTCCGGCCGCGCTTCTGGCGACGGCCCGGGCGTTTCGTGGCGACGCCGATGCGTGCCGCGAAGGATGTGGCCGATGAGCCTGCTCTCGGTCGCCTTTGACACAGGACAGGAGTCGACCAGCGGCATGCATGCCTCCACCAGCGCCACGACGGCCGCCGGCAGCGACGAAACGCTGCTGGCGCGTTACCGCAAA

F – ggatccactagttctagaGATAGCCGCTCCGGCCGCGCTTC

R – ctatagggcgaattggagctcTTTGCGGTAACGCGCCAGCAGCGT

**Pant277**

TCGAGGTTCCTCGCCGGCGACGGCGGGGCTAAGAGGGAACGCGGTCGATGCCGCGGCTGCCCCCGCAACTGTGAACGGCGATCGTTCCCCAATGCCACTGCGTGACGCGGGAAGGCGGGGAACCGGCGGAGACGCCAGACCGTGAGCCAGGAGACCTGCCTCGTCGATCCCGTGGCGCGATGCCCGGGGACAAGACTTCCATACAACCGGGCGGGGTGATCCGGTGGCGAATCCGTTGCGCGGCCCCTGCGTCCGCGCCACTCGTCCGCATGCCCG

F – ggatccactagttctagaTCGAGGTTCCTCGCCGGCGACGG

R – ctatagggcgaattggagctcCGGGCATGCGGACGAGTGGCGC

**Pant278**

CCATCGATCCGCCGGCGGGACGTCGGCTGCCCATTTCCGGCGAATCCGCCGGTGGCTCCACGTTCGTTCAGGAGTTCTCCGATGAGTGTCCGTGAATTCCCCATCCACTACATCGAACCGGTGTTCCGGCCCCCGAGCGAGGCGCATTCGCTGATCCTCCCGGTGACCAATGGCTGTT

F – ggatccactagttctagaCCATCGATCCGCCGGCGGGACGT

R – ctatagggcgaattggagctcAACAGCCATTGGTCACCGGGAGGA

**Pant279**

GTTTCACCGTGGTCGGCACCTTCCGCGTCGGCGCCGGCGAACTGGATGGCGGCCTGTCGCTGATCCACCTGGAAGACGCCGCGCGCCTGCAGCGCTGGAAGACCAACCAGGTACAGGGGCTGCGCCTGAAGCTCGACGACCTGTTCCAGGCGCCGCGGGTGGCCTGGGAGATCGCCCGGACCCTGACCGACAATGACTTCTACGCCCGCGACTGGACGCGCAGCCAC

F – ggatccactagttctagaGTTTCACCGTGGTCGGCACCTTCC

R – ctatagggcgaattggagctcGTGGCTGCGCGTCCAGTCGCGG

**Pant282**

TTGAACTTACACATCGGACGCCGTGTCGGGACCCTTTCGGCAACCGCCGAGCATAGTCCCAAACCCTTCGGCACACCCGCCGACGCGATAACCGGGTCTCTATTGTCGAGGCCTGCGCGAGGCAAACGCTCGATTCATACGCCCGTTTGACTTGGGTGCGCACACATTCCGGCAAAAGCCGGGAATTGTCAATCAGCCATCCGGAAAACACCTGCCCTCCTCCAGGCCCTCTGCGCAGGGCCATTCAGCCAGCCTCTGTCGTACCATTCAGATGTTCGATGATCGGCGCCG

F – ggatccactagttctagaTTGAACTTACACATCGGACGCCGTG

R – ctatagggcgaattggagctcCGGCGCCGATCATCGAACATCTGA

**Pant283**

ATTATGGACTTTCGCGCCGCGCGTGGAACCTGTTCCGTTGCGCTGGGCAAAAAGCTGCTCCGCCGTGGTCGCATTCGCCTGTAAACTGCGCCTCCCTTGGCGACTGCGACCGATCCTGCCTGCGCGCGCGGATCGAGGCAAATGCCTTAACCGTTTTTCGAGTGTCCGTTCCTTATG

F – ggatccactagttctagaATTATGGACTTTCGCGCCGCGCGT

R – ctatagggcgaattggagctcCATAAGGAACGGACACTCGAAAAA

**Pant284**

GCAATCCGGCCGCGCCGCGCGGAGCTTGCCGGGGTTCGCCCGCGGCTCCAGCGGCGTCGGCTACTGTCTGTAATGCGAGGCTCCGCCAATGGAGCGTCAATCCAGGAGATGCCGCCATGGCTACTAATCGTTCCCGCCGCCTGCGCAAGAAATTGTGCGTCGATGAATTCCAGGAGCTGGGTTTCGA

F – ggatccactagttctagaGCAATCCGGCCGCGCCGCGCGG

R – ctatagggcgaattggagctcTCGAAACCCAGCTCCTGGAATTCAT

**Pant287**

CTACAAGGATTTCGTCAAGGCCCTGAGCCTGGCCCTGCCGCGTTTCACCAACGGCGACAGCATCCGCCTGCGCCAGGCCGCCTGGCACGGCGAGCGCCTGTTCTGGGCGGGCGAACGCCAGGCCTGCGCCTTCGCCGCGGCGATCGTCTACGCCCGCCGCCGCGAACTGGAACTGAAGCTGCCGGCACATCTGGAACGCTACGAACTGGAGCCCGGCGCGCTGGACGAACTGGAACAGCGCTAC

F – ggatccactagttctagaCTACAAGGATTTCGTCAAGGCCCTG

R – ctatagggcgaattggagctcGTAGCGCTGTTCCAGTTCGTCCAG

**Pant294**

GAGGCAGAGCAGGAAAATCTGGACTTGAACTCGGCAAGGCGGTTTCGCGACAGAAACCTCCTACAACACGAGAATGACCTCCAGCAGCACCTCCCTGCGCGGCAGCACCTTGTCGTTCCAGAAACGCAGTACCCTGGACCCCGGCTCTCCAGCCAGGCATCGGCCGGCGAACCCACATGC

F – ggatccactagttctagaGAGGCAGAGCAGGAAAATCTGGACT

R – ctatagggcgaattggagctcGCATGTGGGTTCGCCGGCCGATG

**Pant295**

GTTTGCGCGGGCATCGATTGCTCGGGCTGAAGTTTCGTCGGCAGAAGGTTCTCGGGCCGTACATCGTCGATTTCGTCTGTCATGAGCGGATGCTGGTGATCGAACTGGATGGTGGGCAGCATGTGGGTTCGCCGGCCGATGCCTGGCTGGAGAGCCGGGGTCCAGGGTACTGCGTTTCTGGAACGACAAGGTGCTGCCGCGCAGGGAGGTGCTGCTGGAGGTCATTCTCGTGTTGTAGGAGGTTTCTGTCGCGAAACCGCCTTGCCGAGTT

F – ggatccactagttctagaGTTTGCGCGGGCATCGATTGCTCG

R – ctatagggcgaattggagctcAACTCGGCAAGGCGGTTTCGCGAC

**Pant296**

CGCAGCCACGGCACACGTCGGCATCGAACTCACAGATATTGATGCAAGGACTTTTCAAGCGTTCCCCCTTTTTCGGCGCGGACCGCAACCGGTCCTCGCCCGTCACTTCACGACAGGCGGCGGGCCGGCGTCCAGGAGGGAATGACTATCGGGCCGATCGAGCGGCCCGCGGTCACTCGGCCTCGGCGTCGCGCAGGCTTTCGTAGGCAGGCGCGGCATAGATGCCGGCCTCGACG

F – ggatccactagttctagaCGCAGCCACGGCACACGTCGGCA

R – ctatagggcgaattggagctcGCGTCGAGGCCGGCATCTATGCC

**Pant297**

CCTCTGGATGTTCTGAAACAGTCATCCTGTCTTTCCGAATGCGTCAGGCCTGTGCCGCAGGCGCATTCGCCAGGTGGAGCAGGCTCCCCGGACCCATGAACGGTACGTCTCGATACCGCGAGGCAGCGAATCGGCGTTGCTTCATCACCATTCTTCACTGGAATCAGGATTCAGCCATGCGTGTCGATCTGCATTGCCACAGTACCGCCTCCGATGGCGTGCTCGCGCCCGCCGCCCTGGTGCAGCGGGCCCACGAGAAAGGCGTCGGGCTGCTGG

F – ggatccactagttctagaCCTCTGGATGTTCTGAAACAGTCAT

R – ctatagggcgaattggagctcCCAGCAGCCCGACGCCTTTCTCG

**Pant298**

ACTCCCGGTAGCGACAGATGGAAAAATGGCAACACCCTGAAAACCACGGCATCCCGTACGTCAAGAGAATGGACAGCTACCCCGTACAGGACGCCGACGACACGATAAGCGAAGTCGCCCCTCCGGCGCACTCGTCATCGCCTGCCGGCCGCGGAAACGCCGGCGAGGCATCCCTGCATTCGAGGAGGCGGAAATGAAAGCGCCACGCG

F – ggatccactagttctagaACTCCCGGTAGCGACAGATGGAAAA

R – ctatagggcgaattggagctcCGCGTGGCGCTTTCATTTCCGCCT

**Pant300**

GACTTATAACGAGAACGCCGGATGCCCGCAAGCGTGGCAGATAGCCGGGGCGGCGTCGATCGGCAACGCGGCGTCCCGCCGGGACGGCGGGCAGGGTAGAATGGCGGGCTTTGATCGTCGTCGGAACCGTCCATGGATCGCCCCCGTTTCTCCAGCGCTTTCCTCCATCCCCGCTACTGGCCGCTCTGGTTCGGCCTGGGACTG

F – ggatccactagttctagaGACTTATAACGAGAACGCCGGATGC

R – ctatagggcgaattggagctcCAGTCCCAGGCCGAACCAGAGCG

**Pant301**

CTTCATCTTCGGTGTCGGCCTGCTCGACGATCTCGCCGTCCTGGCCTGGGTAGTGCGTCGCTGGCAGGCCGAGCTGGATGCCTTCAAGGTCTGGCGCGACGCCCAGGGCGTCGAGACGCAACAGGCGCTGCGAGAGCTGCCGGCTCCCAGGGCGGAGCGCGTTCAGGGTTCCTGAGGCGCTTGCAGGCTGTTTTCCAGCAGGCGCCCGATGCTGCCGTCCCTGACCA

F – ggatccactagttctagaCTTCATCTTCGGTGTCGGCCTGCT

R – ctatagggcgaattggagctcTGGTCAGGGACGGCAGCATCGGG

**Pant304**

TTGCAAGGAACTGGTCGCCCGGTCCAGCGACTTTCTCGATGGGCAACTGGACTTCCGTGGACAACTGGCGGTGCGCAGCCACCTGCTCATGTGCCGGCATTGCCGGCGCTTCATCCGCCAGATGCGCCTGACCCAGGCGACCGTGCGCCATCTGCCGGAAGGGCAGGGGCCGGAACTCGACCGTCTGGCCGCGCATCTCTCCGAATTGCGCAAGGACGCCGCGCGGCGCTGAGCCGGGGTACCC

F – ggatccactagttctagaTTGCAAGGAACTGGTCGCCCGGTC

R – ctatagggcgaattggagctcGGGTACCCCGGCTCAGCGCCGC

**Pant307**

TCACTAGCGTCGTCGGGGTTCGAACAGCTTGCTGCCGAGTGAGCCTGCGGGCGTCATGGATGCGCTCGGCAGCCCTGTGCCACACCCTGACCTGGTGAGTCCCCCGATGATTTCGAAAAGCAGAAGAAGCTTCATCCGCCTGGCCGCCGGTACGGTCGGCGCCACCGTCGCCACCAGCATG

F – ggatccactagttctagaTCACTAGCGTCGTCGGGGTTCGAA

R – ctatagggcgaattggagctcCATGCTGGTGGCGACGGTGGCGC

**Pant308**

AGCAGGAATGGCTGGCTTTTGCAAAGTCTCACGTCGCGCGCCCGTTATTTTCAATTATTCTATCGAGCGACATTTTTCAGGCTTACGGAAATTACCTTCCGTCTGCCGGCCTTGTTTGACTCGCCCTGACGTAACAATGGACAAGCGCCGGGAAAGTTCCAGCTTCCGCGGCGCTTGCCGGTATTTCCAGTGAATTGTTTTCAGGCGTTCGCCGCGCGCCGGGCCGCCGGCGAGTTGGCCAGGTATTCCAGGGCTTCCTGGGTATTCGCCTCGTGCTCGGGGTGGTACC

F – ggatccactagttctagaAGCAGGAATGGCTGGCTTTTGCAAA

R – ctatagggcgaattggagctcGGTACCACCCCGAGCACGAGGCG

**Pant310**

CGCTCTTGCTTCGGGTGCCGCCGCTTCCCTGCCAACCACGCTGGCTACGGCGCGCTGAAACGTCGACGGGTGGAACCTGTCCGGCGAGGCGTGCGGTGCCCGTGTTCGCCGAATCGATGGATTGTTGAAGAGGACGCAGGGATGGTTCGTTTCGCTCGCTTGCCGCTATCGCCCTACCAACGGGACATCTGGGTCGCCGCCGCGCAGTTTCCGGAACTCGACCAGTACACCATCTTCAGCT

F – ggatccactagttctagaCGCTCTTGCTTCGGGTGCCGCCG

R – ctatagggcgaattggagctcAGCTGAAGATGGTGTACTGGTCGAG

**Pant313**

CTGGTCGCCGCAGCGGCGACGGCTACCCACTGGCCGTCGTCCGGGTCGCCGGTTCCATCCCGGCCGCGCCAACCCCCGCATTCAGGCCCCGCTGCGGCGACTGCATTCCCGCTGGATTCGAGGCTCGTACCCTATGACTCCCGATCAACGATTTGCCCGTTGGGTTCAAGTCGCTATCGCGGTATTCGTGTTGCTGTTCGTCTACTT

F – ggatccactagttctagaCTGGTCGCCGCAGCGGCGACGG

R – ctatagggcgaattggagctcAAGTAGACGAACAGCAACACGAATAC

**Pant314**

ACGATGCGTCCTTCCTGCCTGCGCATCGCCTGCCGCCAAGGACTGCGGATTTCTCCCGACACGCCATACCGCGCGCCAGACGGCTGCCTGAAAAGGCAGGCCAGGTATTCAGTGGAGATACACCATGGCAACACAAGGAGTGTTCACCCTTCCCGCCAACACCCGGTTCGGCGTCACCGCCTTCGCCAACTCGTCCGGAACCCAGACG

F – ggatccactagttctagaACGATGCGTCCTTCCTGCCTGCG

R – ctatagggcgaattggagctcCGTCTGGGTTCCGGACGAGTTGG

**Pant315**

GCAAAATGAATTGTCTGGATAATGCTTATCCAGAATATGGATATGGGATAACACGATATCGGTCGGCCGCCGGCCGCCGAGGGCTCCCATCGCTCCCGCGGACGGGGCGCAAGGATAACCGAGCCAACCCCGCCGACGGAACGGCAAAATGTCGCGGGGCCTTTCGCTCTAGACGAGGAAGCCTGGATGAAATTCACCCTCCGCCAGCTCGAGGTGTTCGTCGCCGTGGCCCAGCAGGAGAGCGTCTCCCGCGCCGCCGAGGGGC

F – ggatccactagttctagaGCAAAATGAATTGTCTGGATAATGCTT

R – ctatagggcgaattggagctcGCCCCTCGGCGGCGCGGGAGA

**Pant316**

CTGAAACTACGCTGCGGAGACTGTCAGGCTCATGAGCCCGGCGTGACATTTCTGTCAGACGACGACCGCCCCGGCAATGCGCCAGCAATCCTGGCGCTATCCACGGACGGCAAGACGCCATATACTGTATGGATAACCAGTCAAAGCAACCTTGCGACTCAGAAGGCACAGAGGTGATGAATGGCCGTCGAAGTGGTGTACCGCAGCAGCCGGG

F – ggatccactagttctagaCTGAAACTACGCTGCGGAGACTGT

R – ctatagggcgaattggagctcCCCGGCTGCTGCGGTACACCACT

**Pant321**

CCGCTTCGGAGGGCGGATAACCGTTTGCTGTTATCCGCCGCGCCGGCGTTGGATGGCGGATAACGCCGCTGGCGCTGTCCGCCCCGGGGGACTGCCTGGCCCCGTTCTCAGCTCCCCGCCGTCGCGCCCGCCTCGTCGTTGGCCGGCGGCGTGGCGCTCTTCACCAGCGGCGTATCGTAGCTGCTCGCCTCGTAGCTCACGCAA

F – ggatccactagttctagaCCGCTTCGGAGGGCGGATAACCG

R – ctatagggcgaattggagctcTTGCGTGAGCTACGAGGCGAGCAG

**Pant322**

TATTTTGACGCCGGCCTTCTGACCATTCGTCGAATGATCGGTTCCGGATGTGACGCGCCGGTTTCGCCGTGCAAGTGGCGATACTTGCGGGTCCGGCAATGACCTGTTGTTCGCCGTGGCCTTGGAAAGCCTCTGCGCCGGGGGTATGTTCGTGTTCCCCGTACCCGAGTGAAGCCCGTAGAAGCGAGTCCAGATATGACCGAAACAGCCAAGCGTCCCTTGTACGTTCCCCATGCCGGCCCATCGCTGCTGGAGATGCCGCTGCTGAACAAAGGCAGCGCGTTCAGTACC

F – ggatccactagttctagaTATTTTGACGCCGGCCTTCTGACCA

R – ctatagggcgaattggagctcGGTACTGAACGCGCTGCCTTTGTT

**Pant325**

CATAGTACTGAGACTCAGGCGCGCCGCGGTTTTCCGGCGATGTGCGCAACAATTTGCACAGTTTTTTTGACGTAGCATCCGCGCGATCACGCACTTCGGATCGAGCACGGCGAACAGGGACGCCAGCCGTGCAGGTTTTTCGAGAAACAGGGGACAGCGGATGTTCAACCCGGCCAACCAGACCCACTTCAGCCTCAGCCTCGACGGCCTCCGTCACGACCTGCAGGTTCTCGAATTCAGCGGCCACGAAGGCATCAGC

F – ggatccactagttctagaCATAGTACTGAGACTCAGGCGCGC

R – ctatagggcgaattggagctcGCTGATGCCTTCGTGGCCGCTGAA

**Pant329**

ATACTAATAATAATGAGAATGATTGTCAAAGTTCCCTGGAGCGTTTTAGTCGGGTTTTTTTCGCCCCCCGACAAATGGCTTTGTCGGACAAAGACTTAGCGCCGGCTGGGGCGGGGTGTATGATGGTTGTTTCCCGGGGAACGGCGCCCCTGCCATCGAGGCTTCACGAGGAGCCGGCAGGCTGCGGTGCCGTCCTTCAACCTTCCGCTTAAAGGAGACAT

F – ggatccactagttctagaATACTAATAATAATGAGAATGATTGTCA

R – ctatagggcgaattggagctcATGTCTCCTTTAAGCGGAAGGTTGAA

**Pant330**

TCGACGACTAGACCGGTCGTCTGGTCCTCGCGTACGCGCCGGGCCGCGACGGCCGGCGACGACAATGAAGAGGCGAGCCATGAGCAAGACCCTCCACTACCGTGCGTGCCATCTCTGCGAGGCCATCTGCGGGCTCGCCATCGAGACCGAGAGCGACGAGGGCGGCGTGCCGCGGATC

F – ggatccactagttctagaTCGACGACTAGACCGGTCGTCTGG

R – ctatagggcgaattggagctcGATCCGCGGCACGCCGCCCTCG

**Pant332**

TCCAGCGCTGTACTATCCCTTCCAGCGCTGATCGCGACCCGGTGCCCGCACCACCGTGCCGCACGAGCCCCGGAGATCCGCACCTCCGGGGCTCACCTTTTGCTCCTCTCGCACCCTCCTTCCCCGGCTGAGCCCCTCCCCCTGCAAATCCGCGACGAATGGCTGCAATCCGGCAATCCGATGAACGAGCGGCCGAACCG

F – ggatccactagttctagaTCCAGCGCTGTACTATCCCTTCCAG

R – ctatagggcgaattggagctcAAAGGTGAGCCCCGGAGGTGCGGA

**Pant335**

ATAGTCACGTAGGGCGAATACCGCCACAGGCGGTATCCGCCGATGCCCTGGAGAGCCGGCGGATAACCGCAAGCGGTCATTCGCCCTACGGATCGGGCTCCTGGCATGCCCTCCGAGTGGCAGGGCGAGTCTTTTGGACGCTTGCAGAAGGAGCTCGCCTGGAAAGTTGAAACAAGAGGCCAAGGCAAGCACGACAAGCAGCTTG

F – ggatccactagttctagaATAGTCACGTAGGGCGAATACCGC

R – ctatagggcgaattggagctcCAAGCTGCTTGTCGTGCTTGCCTT

**Pant339**

CTGATTATTCCTTGAAACGCGGTTTTCTACACTTTCCGTTTCAGTGGGTCGCTTAGCGGCCCGCCGGCTTCCATCGTTCAGGAGTTCCCAGATGATCAAGTCCCGTGCCGCCGTCGCCTTCGCCCCCAACAAGCCGTTGGAAATCGTCGAGGTGGATGTGGCGCCGCCGCAGAAGGGCGAGGTAC

F – ggatccactagttctagaCTGATTATTCCTTGAAACGCGGTTTT

R – ctatagggcgaattggagctcGTACCTCGCCCTTCTGCGGCGGC

**Pant340**

TTCCAGTAGACTACGCTCCCGTCTTGTACTGCTCCCGCCGATTCCCTCCGGAATCAGCAGGAGTTTGCTTTGCCCACGGATTTGTCCGTGGCGGGAGCTGGCCTCCCTTCTCCGAATCCCCGGCGGTTGATCCCGCCTAAACGCTGACTTCTAGGGTTTTTCATGACGCGCTACATCTTCGTCACGGGTGGTGTTGTTTCTTCATTGGGGAAAGGCATCGCTTCGGCTTCTCTGGCTGCGAT

F – ggatccactagttctagaTTCCAGTAGACTACGCTCCCGTCTT

R – ctatagggcgaattggagctcATCGCAGCCAGAGAAGCCGAAGCG

**Pant342**

TTTCGACTGTCGCCCCGGAAGAGGCTGCCCGCTGATGCGTGCGGCCTCTTCGTCAAATGGGGAAGCGTTCGGGGCAGCTTCCGCGTTGCCGGCCCGCTCGGCGGGCTTGCACGCAACGCAGGCGCAAAGTCGTCTCCGCCCACGACGGCCCGGCCGTCACGTATTTTTATCGCCGCAGGAGAAAAGGTCATGGCTCAGCAACTGAGCGCTCGTCAACCTCGCTATAAACGCATTCTTCTAAAGTTGAGCGGCGAAGCCCT

F – ggatccactagttctagaTTTCGACTGTCGCCCCGGAAGAGG

R – ctatagggcgaattggagctcAGGGCTTCGCCGCTCAACTTTAGAA

**Pant343**

TTTTCCGGAGCACAAACCCACACATGCATCGACACGATGGCCTGGGTGCCCGCAAGGGTTGGTCATTGGGATGCGTGGAGGCCTAACCCGACTTATCGAGGAACTATCATGTCCCAAGTCAACATGCGCGATATGCTGAAGGCCGGTGTGCACTTCGGCCACCAGACCCGTTACTGGAACCCGAAAATGGGCAAGTTCA

F – ggatccactagttctagaTTTTCCGGAGCACAAACCCACACAT

R – ctatagggcgaattggagctcTGAACTTGCCCATTTTCGGGTTCCA

**Pant344**

ACATGGCCAATCGCCAAGCCCCTGAAACAGAGGCCTGGCAGAGGCTTGCTCAGTGAGCAGGAAGAAAAGACCGGAGGGCCGGAAACGCGCCCACGGTAATGGCCAGAGATGAAAGCCAAGGAATCAGCATCCCTGTCAGAGGAAATTTCCCATGGAGCAAAAAGCGATGAGCAAGCAACACACCGGTTCCCTGCGTGGCCTCA

F – ggatccactagttctagaACATGGCCAATCGCCAAGCCCCTG

R – ctatagggcgaattggagctcTGAGGCCACGCAGGGAACCGGTG

**Pant346**

ATTTCCGGCGAAATCAAGGCTACCTGCCAGTTCTGGCAGGTTTGGCCGCGGGTTCTTTTTGGTACACGAAAGCACCGTCGAAAACGGGACCGAGCCAGGGGAGTGCAGTTCCTTCTACCCGAAGGACTGATACGGCTGTTCCGATCAGCCCACAAGGCGGCGGTAAGCGTCGGCCGAGTACTTCGGCCTGAAAAAACCAGGAGAACTGAACAAGATGAAGAAGGTTTCTACGCTTGACCTGTTGTTCGTTGCGATCATGGGTGTTTCGCCGGCCGCTTT

F – ggatccactagttctagaATTTCCGGCGAAATCAAGGCTACCT

R – ctatagggcgaattggagctcAAAGCGGCCGGCGAAACACCCAT

**Pant347**

GTCGATACAGGATGTAGCGCGCCAGACACCGAGCCAGGGAGGCCCCTCGTGCCATTTCTTTCTCTCTTTCCTCCCCGTGTTTCTTTTCCATGCTCCGGCCGGCCCGGCGTAGATATGCGCGTGCGTTGGTTTTTATTACATGTGATCTGTTGAAATCTTACATGTGATCTCGTAGTGTGAGTCCATGCAACGGAAATCATCGGCCCATTATCAGCGTGTCTTT

F – ggatccactagttctagaGTCGATACAGGATGTAGCGCGCCA

R – ctatagggcgaattggagctcAAAGACACGCTGATAATGGGCCGAT

**Pant349**

CTCTTTTGTTTTCATCATTGGCGATTTCCGCCGCCCGCTGGCGGAATCCATCGCCAGGATGTGTCGTGGCCCGTACAGTGGTCGCGTCAACCTCGATTCGCTCGGGGAACCATAGCAGGCCGGCGCGTCCGGCCGTCGTAGGTGAGATGGGGAGTTCCAGCGTGAAACCGGTCAAAGTAGGCATCTGTGGGTTGGGGACCGTCGGTGGCGGTACCTTCAATGTA

F – ggatccactagttctagaCTCTTTTGTTTTCATCATTGGCGATTT

R – ctatagggcgaattggagctcTACATTGAAGGTACCGCCACCGAC

**Pant351**

ATCTACCACAGCCGACGCCAAAAAGAAGCCCCGCGGATGCGGGCTTCTTGTCGCGCCAGGGGCGGTTTGCACCTGTTCGGTCTGCGAGTCTTTTGCTCATCCGTGACGAATAAGCTGGCAGCTCGCCCCTGGCGCGGGACTTTTAGCCGCTAGCCTCGGTCCGCCGGAAGTGCGACGACGGAAGCCCCCTGCCCTTCCTGCACAGTCAGGCTGGAAAGCAGACTCTCCAGCGGCATCGGCCG

F – ggatccactagttctagaATCTACCACAGCCGACGCCAAAAAG

R – ctatagggcgaattggagctcCGGCCGATGCCGCTGGAGAGTCT

**Pant356**

CGTTCATTCCTTAACCACTACGAGATCGCCGTGCTCAAAGCACTCAAGAAAATCTTCGGCAAGGCCGACGGAACCCAGCCGCAGAGCGGTTCCTCCCCGGTCCCGCAAGGCCCTGCCGGCGCTACCGCGAAAGGTACCGCCCCCACCGGCGAAAAGCCGACCGAGAAACGCCCACGCAAACCGCGCCCCCCGCGCGCCGACAACGAGTCCGGCGCCCCGCGCGCCGCTGAAAAGGCCGAGAAAGCCGAAAAGCCCGCCAAACCCGCCGGCAAAGAAGGCCGG

F – ggatccactagttctagaCGTTCATTCCTTAACCACTACGAGAT

R – ctatagggcgaattggagctcCCGGCCTTCTTTGCCGGCGGGTT

**Pant358**

ACGGACAGCGTTCAGCGAGAGCCTAGGGAAGGTGTTCCTATGCAAATTTGATAGGAATCAATGAAATACCGGCCGTTCCGTGCCTAGATGCGCGCCCCTACCTCGAAAGAGGTACGGCTGAAATTATTGCGCAACTCACTGTTTTAAAAGGCTTTTCTCATGAACAAGGCGACCGCGCCGGACGCTGACCAGCGGGACAGA

F – ggatccactagttctagaACGGACAGCGTTCAGCGAGAGCCT

R – ctatagggcgaattggagctcTCTGTCCCGCTGGTCAGCGTCCG

**Pant359**

CAAAGGTCGGAAAAAGGATGCACGCATGCACTGGCTGGACTACCTGCAATGGCCCGCCATGCTGGTCACGGTGGTGGCCGCCTGGCTGGTGGCGTCGGCGCATCGGCGAAGACGCAAGGTCGGCTTCTGGGTGTTCCTCGCCAGCAACCTGCTGTGGATCGCCTGGGGCCTGTACGCCCAGGCCTATGCGCTGATCCTGC

F – ggatccactagttctagaCAAAGGTCGGAAAAAGGATGCACGC

R – ctatagggcgaattggagctcAGCAGGATCAGCGCATAGGCCTGG

**Pant360**

ATGACCGGGCCTGGGCAAGCGGCCGCAGCCGCCGTCTCGCCTGGGCTGGCGGCGAACGAAGAAATTTTCCCGGGGAAGCGGTCGATAATGGTGCTGCCGCGTCGGCGGCGCGCCATGCACCGTATCGGTGCGCTGGACGTCCAGGGCTTTTCTGCGGTTATGATGCGGGTTCCGAAATTTTCTCGGGCTTTTCCAGACATTCGCACCCCACGGTTCACGGATCGGCCTTTTGGGGTAGGTGTTTTAC

F – ggatccactagttctagaATGACCGGGCCTGGGCAAGCGGC

R – ctatagggcgaattggagctcGTAAAACACCTACCCCAAAAGGCCG

**Pant363**

CATTGGAAAGCGACCGCAGCCCTGGATGGCAGGAATGCGGGAAGGGTCGCAAGCAGATTCCCGGACCGCCCGCCTTGATGCAGAGCAATTCGGTACGAAAATCTTACCCGTTAGCCTAGACCGTCCTGCTGTGGTCGGTTGTCGCGAGACAAATTGCCTCAGCTCTCTCATATTCATTAATAGAATATAAAAATAAACAAATAATATTTTTATGGAATAAAAAGAGTCGCTACTCTCCCCTTCAAGCCGCCCCTCGCGTGTTGGCGCCC

F – ggatccactagttctagaCATTGGAAAGCGACCGCAGCCCTG

R – ctatagggcgaattggagctcGGGCGCCAACACGCGAGGGGCG

**Pant364**

ATCCTTAACTAAGAACCGGAGGCATGCGCCCTGGCAGAACCATGAGGATGCGTCGGGCAACCGACGCCGGCATCATTCCGGACAGCCGCGAAAACCTCTGTAACGGCTCTGTCATGCGCGTCGTGCGAGGAAACAAATAAGCGGAGAAAAGGGAACCTTATTCGCGCCAAGCTGTTTAAAATCACGCCCGTTTCTGTGCGAGGCCCCTCGCGCAGTCTTTTACCAAGGAGCTACACCA

F – ggatccactagttctagaATCCTTAACTAAGAACCGGAGGCAT

R – ctatagggcgaattggagctcTGGTGTAGCTCCTTGGTAAAAGACT

**Pant365**

TTTGGTGCAATCCATCCTTCCTTCTCGAAATGCAGTCGAGGCGTGCGATGAAATCCGATGAGAAAACCCTGATCGACGGCCTGTTCAGCCGCCTGCGCGAGGCCGAGGGGCAGTCCGCTCCGCGTGACGCCGAGGCCGAGGCGCAGATCAACCAGCACCTGGTGCGGCAGCCGGCGGCGCCGTACTACATGGCGCAGGTGGTGCTGATCCAGGAAGCCGCGCTGAAGCGCCTCGACCAACGGGTGAAGGAGCTCGAGGCGCAGGT

F – ggatccactagttctagaTTTGGTGCAATCCATCCTTCCTTCT

R – ctatagggcgaattggagctcACCTGCGCCTCGAGCTCCTTCAC

**Pant373**

ATCCAAAAAGCCCTGTTTTTCACGAAACAGGAGTTCGTCATATGTCTTCATCTTGGAATACCCGCGCTATGGCACGCAGGCCGCTCGGCGTCGCGCCGGCCATGCTGCGCGCCTGTGCGGCGGAAAAACGGGAGGTCGCATGTTCACCGGCATAATCGAGTCGATCGGCAGCGTCCGCGCAATGACGCCCAAGGGCGGCGACCTGCGGGTCTACATCGCCACCGGCAAGC

F – ggatccactagttctagaATCCAAAAAGCCCTGTTTTTCACGAA

R – ctatagggcgaattggagctcGCTTGCCGGTGGCGATGTAGACC

**Pant374**

TCACCACCAGGGCTTGTTCAGCGCCAGGTAGAAGATCCCGCCCAGCAGGGTGAGGCTGAGGACGCTGAGCAGGCCCTTGCCGGGGCCCCGCGCCATCCGCAGGGCGAACGCCGCGCAGACGATGTAGCCCAGCAACAGCCCCAGCTTCGCCAGCAGCCAGAGCGGCAGCGGCCAGGGCATCGCCAGGTGCA

F – ggatccactagttctagaTCACCACCAGGGCTTGTTCAGCGC

R – ctatagggcgaattggagctcTGCACCTGGCGATGCCCTGGCCG

**Pant375**

GTGTCAGGTTGCATGAAAATAACGGCGGCGCTGTCCGGGGTACCTGCCGCGACAAGCAGCACCCGATGCCCGCACCTTTCATGTCGCCAGCGAGGCGCAGGATGTCATTGCCTTGGGGCGTGCGCCGCGTCGCTATTCACTATCAGATTTCGAGTGCCCCCCTACACCATGCGAATGCGCCTGATGCTTTTGGGCGGCGGTAATGCCCTTGGACAGGCGCTGATCCGCCTAGGGGCGGAGGAA

F – ggatccactagttctagaGTGTCAGGTTGCATGAAAATAACGG

R – ctatagggcgaattggagctcTTCCTCCGCCCCTAGGCGGATCA

**Pant376**

ATTAAATATCGTCGGTTTCACTAGAAAATACACATGTTCACTTTGAACCGGGGCAGTGGCGTCGGCGGATTCCGGACCGCGCTTTCCTCCTTGCCGGCCGAGCGGCGGCCGCCTGCCTGCGGCAGGCATGACCGAGACCACAGGTTTCCTGGTAACGCTTTCGCCTGCCGTGTACCGCTTAAGGAGACGCAAGGATGTCCCTACATCGCATTCGCGTAATGGTTGCGGATGATCATCCCGCCATTTCGCTGGGTATTTCCTACGA

F – ggatccactagttctagaATTAAATATCGTCGGTTTCACTAGAAA

R – ctatagggcgaattggagctcTCGTAGGAAATACCCAGCGAAATGG

**Pant378**

AATCCGCCGACAGTTTTCAAGAGGCCATATGAGTCCGCTCCTGGAGACAATCGAAGAGCACAGCAGAAATGGCTGTGCTCCATCCAAAGCGTTGATTGCCCTGTGGTCCGCAGGCGCCGACCTGAGGGTTTCCCCGAGGAAGGCTGGCCGCCTATGCGAACTTGCCGTCGTTGCCGCAGCAGGGAAAGCAGGCATGCTCGAGAG

F – ggatccactagttctagaAATCCGCCGACAGTTTTCAAGAGGC

R – ctatagggcgaattggagctcCTCTCGAGCATGCCTGCTTTCCCT

**Pant379**

TCAAGTGTCGATGAAGAAGTAATCACCTTCCTCGCGCCTTTTTTTATACGCTCGGGCCCTCCCAAGGTTGATACGGTTTGAGGCGTTGGGGTTGTGATTGGAATTGCTGACGGCGTGGCGGATATGGACCGGCTCGTCGGCGTTGCTGATCGCTCCGCTAAGCGTCACTCCCAGGCTGGCGGCATAGGCCCGGCGTGCCGCAACGATCTCGCTCTCGAGCATGCCTGCTTTCCCTGCTGCGGCAACGACGGCAAGTTCGCATAGGC

F – ggatccactagttctagaTCAAGTGTCGATGAAGAAGTAATCAC

R – ctatagggcgaattggagctcGCCTATGCGAACTTGCCGTCGTTG

**Pant380**

ATTCGGCAGTTCCCTCCAATACCTATAACGACCATCCAACGCACATCGCCGCCCCGGACCCTTGCGTCCGGGGCGTTTTCCGTCGTGCGTGGCGAGGGTCGCGGGACCTGCCCGTCCGCCCCCTGCTCCGGAACCGAACGCCCCATGGACAGCACCTCCGAAACCCGCAGCGGCAGCTGGCTGAGCGTGATCGCCCTGGCCCTGGCCGCCTTCATC

F – ggatccactagttctagaATTCGGCAGTTCCCTCCAATACCTA

R – ctatagggcgaattggagctcGATGAAGGCGGCCAGGGCCAGGG

**Pant381**

ATGAATCGTGCAGCCCTGACCTTCAAGCGCTATTACGCCTATCTGCTCCCTCATTGAGGCGGTAGATGCGTCGCTGCATTCCCGAACCGCCCGAGGCGGCGGTCCGGTGAACTCCCTCCCAACCTGTCTCCTCGGGCATCCACCGTGCCAAAGGAGATACCCGTGTCCGTCCCGACCCATCAGCAAGACCTGATCGCCCTGCTCGAAGAGCGTGGCTTCGTCCACCAGTGCACCGACCGCGACGGCCTCGCCG

F – ggatccactagttctagaATGAATCGTGCAGCCCTGACCTTC

R – ctatagggcgaattggagctcCGGCGAGGCCGTCGCGGTCGGT

**Pant382**

ATGATTAAGGCCGGTTCGTGCGACACCTCGATAAGACCTGTTGAACAAGTCAACGCCGTATCGAGGGCTCATTCATATCGGCTCCGGCCAACCGCTCTTCGTCCGAGTCAGTACGGCCTCGCATAGCGGCATTTCATGGCATCAGCCGGTCGAGCAGCGGCGAGCTGAACAGCCGATGCGGATCGGCTTCGTTCAACTGGCGCACCGCGCTGTCCCAATCGTTGTCTGCG

F – ggatccactagttctagaATGATTAAGGCCGGTTCGTGCGAC

R – ctatagggcgaattggagctcCGCAGACAACGATTGGGACAGCGC

**Pant383**

GAGCCTTGCCCCGTTAATGCCGGTGGGCTTTCCCTGTAGCGTTCCGGGCAGCGGCGCAACCCCTTCGGTCGCGCCCCGCCAGGAGCCTACGGAGGAAACCCATGCCCGCCCCGCACCAGCCAGCCCGCAATCCGACCGGGGAGTTCGACCCATGCCGCTAGCCCTGCTGCTCGCCATGGGCGCGTTCTCCCTGAGCCTGTC

F – ggatccactagttctagaGAGCCTTGCCCCGTTAATGCCGG

R – ctatagggcgaattggagctcGACAGGCTCAGGGAGAACGCGCC

**Pant384**

GGCGAGAGAACGAAACCCGCAAAGGGGAGCGAAAAAGCCGGCCAGTCGAATTGTTATGGACAAGCGCGCGACCGGGACGGGGCTACCGTATGGCCAAGGGCCATGCGCCTGGCCCGGTTGGGCCGACGGCTGACAGACAGCGCTCGGGGCGATTGAGTTCACTCGTTTCCGCAGGAATGGATGGATGGTTGTAACAGCAGAATGCCAGGCCTGCCGACGGCCAGCGCTGCCCAGGCTGCGACCTGTCCGGGAGGTCCCCGGGTACGACGGTCTCTACGCCAAGGCAGAGGCTCCCT

F – ggatccactagttctagaGGCGAGAGAACGAAACCCGCAAAG

R – ctatagggcgaattggagctcAGGGAGCCTCTGCCTTGGCGTAG

**Pant386**

GTTTAACTTCCATATATTTTTCATCGTTGCTATAAATCGCCCCAACCCAAGGAGCGCTTCGCCTGGTCCTTGGAAGCTGTTCACGATCTGCCGAGCCAGAGAAACCAGCCGTTTTCGACGCAGTCTCTCCGCGCGCGGCAGATCGTGACCCTTACCCAGAACAGAGCTAACAATCAGCCACGGGGTACTCTTCATGTCGATCTTCGAACAGGGTCTCGCGCCCGCCGCCGTCAACCACATCGCCCTGACGCCGCTGAGCTTCATCGAGCGCACCGCCAGCGTCTATCCGCA

F – ggatccactagttctagaGTTTAACTTCCATATATTTTTCATCGT

R – ctatagggcgaattggagctcTGCGGATAGACGCTGGCGGTGCG

**Pant390**

ACGACATTCGCCGAAAATTCGCGCTTGAACTCGCAAATTTTACCTTGACTTGAATGATCACCAGTGAAAGAGATCATTCAGTCTTACTTCTATCACATACCCGAATTTTTAAAGAACAGTTCTGGTGCAAAGACCAGAAATCAATGTTCATTCACGAACATTCATTTCTGAGCTTTCGACGATTGTTTTAGATGGTGGAGC

F – ggatccactagttctagaACGACATTCGCCGAAAATTCGCGC

R – ctatagggcgaattggagctcGCTCCACCATCTAAAACAATCGTCG

**Pant391**

ACACCAGCCAGAACGCCTTGTCGGCCGCCGGCGTGGCGTTCTCGGTGAAGCGCCTGGCGGAGAAGCTGCAATCCCAGCTCGACGCCGCCGAACAGATCGTCGGCAGCGCCGAAGTGATGATCCACACCGAGCGCGCCACCTCCAATCTGGCGCGCCAGGCCATGGACGCCGCCACCCGGGCGCGCGCCGGCAGCGACGAAGGCCAGGTGCTGCTGGCCTCGACCATCGAGCGCATGCATCGGCTCAGCGAGCGGATGGGCGCCAGCCGCGAGGCGATCGAGGCGCTGAACC

F – ggatccactagttctagaACACCAGCCAGAACGCCTTGTCGG

R – ctatagggcgaattggagctcGGTTCAGCGCCTCGATCGCCTCG

**Pant393**

GCAAGGTTATATGGCGGGTTACAAGGCTTGGACTACATCTAATGACAAATTGACATTGATTATCCCGAGTCTAGATTCAGGCCTATCGTCGCAAGTTCGAAGTTATGAACTTTGGTTCTTGAATAACGACTGTCGCCAGTCGATAACCCCAATGTACCTGCCATGGAAAGCCAGTCCCGGAGTATGGCCATGACACGGACAGCGAGACGCCCGTTTTTCTTTTCCGATCATCGGCGATCGCCGGTGATTTTCCGTTCATCGACCTGTCCGGACGCATAGCAGGG

F – ggatccactagttctagaGCAAGGTTATATGGCGGGTTACAAG

R – ctatagggcgaattggagctcCCCTGCTATGCGTCCGGACAGGT

**Pant395**

GCAAGGTTATATGGCGGGTTACAAGGCTTGGACTACATCTAATGACAAATTGACATTGATTATCCCGAGTCTAGATTCAGGCCTATCGTCGCAAGTTCGA AGTTATGAACTTTGGTTCTTGAATAACGACTGTCGCCAGTCGATAACCCCAATGTACCTGCCATGGAAAGCCAGTCCCGGAGTATGGCCATGACACGGAC AGCGAGACGCCCGTTTTTCTTTTCCGATCATCGGCGATCGCCGGTGATTTTCCGTTCATCGACCTGTCCGGACGCATAGCAGGG

F – ggatccactagttctagaGAACTGTATCGGCGCGTATTGGCG

R – ctatagggcgaattggagctcGCCAGTGGTCACCTGGGCGACGA

**Pant401**

AGGAAGGCGCGGTGTCACCCCTGTGACAGGGGCGTGATGCTTGTATGACATTGTCGCCGGATGTAACGCGCCGTTGTTCAACGCTGGCACCCTCGGCAGGATGCCGGTTCCGACACATTCCACACTGGAGTCCGCCATGTCCTGGCAACGCTTGCTCGGCCCGCTCGACCGCCTGCCCACCGAAGGCTCCCTGGAAGACTGGCACGCCGCCGCCCTGGCGCGCGCCGACGG

F – ggatccactagttctagaAGGAAGGCGCGGTGTCACCCCTG

R – ctatagggcgaattggagctcCCGTCGGCGCGCGCCAGGGCG

**Pant404**

TCCGCGAAAGTCGGAAGCGACCTGCAACACGCTTGAAATCTTTCGCCAATCCTGTGCTTTGACAAGCCCTGAAAGCTGCACGTAATGTGTCGGATTCGCCCCGAAACGGGGCGCTCTCGGCATACCAGGGACCAGGATTATGTTGCTCCGCCGCATGTTGATCATGCTCGCCGCGGTGATCGCCGTGGTGGCGATTCTCGCCGGCTACAAGGTCTACTCCATCC

F – ggatccactagttctagaTCCGCGAAAGTCGGAAGCGACCTG

R – ctatagggcgaattggagctcGGATGGAGTAGACCTTGTAGCCGG

**Pant406**

CGCTTTTTCCCGGTTTTATCCGTCCGTCGCAAAAGCGAAAAGCCGCAACTCTTGATAAATCACTTTAAGTTCGAAGGCTAAGACTTTGGACTTATTGTGTTTTGTCATCTCTTTTACTTCTAAAAGCGCTCCGCAACCTTCCAAAAGCACGCTAAATCTCCGTCCTGTAAGGATTTTTCACTTCTGACGCGCCTTGACGGTGGGG

F – ggatccactagttctagaCGCTTTTTCCCGGTTTTATCCGTCC

R – ctatagggcgaattggagctcCCCCACCGTCAAGGCGCGTCAGA

**Pant407**

TGCCCCGCTTACACCAGCCATGCTGTGAAGTCGGTCCGCATCCCCCGCGGCGCAATGTTGTATGGACTGCAATGCTCTGTTACGCCGCCGTTATCTGCACTCCGCGCTATGACTGCATAAATGTCGATATATTCGACGTCGACGCGATTTTTTTCTGTCCGTAGTGAACCTGAAGTGTGCGATATGTCCTAAAGGGCTGAGAGAACGC

F – ggatccactagttctagaTGCCCCGCTTACACCAGCCATGC

R – ctatagggcgaattggagctcGCGTTCTCTCAGCCCTTTAGGACA

**Pant408**

AACGGATGCCGCGGTCGGCGGTCTAATTCCGCCTGGACGGGCCTTGTGGGGCCGGGAAAAGCCTGCAATTTAGCATATTGCCCGCCTTGCCCAAAATCCCCGTCGTTGTTAGAGTTTGCCGCACTTTTTTACCCGCGTATTTTTCACCCACGCTTCGCCTAGGGTTTCTTTCCCATGTTCAAAAAATTGCGTGGCATGTTTTCCAGCGATCTGTCGATCG

F – ggatccactagttctagaAACGGATGCCGCGGTCGGCGGTC

R – ctatagggcgaattggagctcCGATCGACAGATCGCTGGAAAACAT

**Pant410**

TCCTTGTACTAAGGCTGAATTCACACTGTAGAGCATCGTTCCGGCAGGCGCGGCAAACACGCCTATACGCCAGTCTTGGACCATGAGACGAAGGCTTTTCCCGGAACGGGTTAGGCGATTTCGGAAAAAACGTGAAATTTACTGATCTGAGCCCTGACAGCGCTCGGCGAAACAGGGCAGGATGGCGACCTGCGAGGTTTCTGAGTCACTTTGCCGCAGAGTTGCCG

F – ggatccactagttctagaTCCTTGTACTAAGGCTGAATTCACAC

R – ctatagggcgaattggagctcCGGCAACTCTGCGGCAAAGTGACT

**Pant411**

TTCAAACGGATAACAACATCTACACTGAACCGACCTCGACGCTTGCGGCCGGCACATGCCCTGCATAGCTTCGCGCAAGACAGCTCACGAGGCTGTCCCTCGCAGACAATCAAAATAACCAGAGGTCACTCCATGCGTAGAAACGCCGTCATCCGCTCCGCGATCATTCCCTCGCTGCTCGGCGCAGCGCTGGTCGCCGCCGTTCCCCAGGCCTTCGCCAGCAACCT

F – ggatccactagttctagaTTCAAACGGATAACAACATCTACACT

R – ctatagggcgaattggagctcAGGTTGCTGGCGAAGGCCTGGGG

**Pant412**

TTTTGCTTTTCCTGCCCGTGTTCCAGAACGGCGCTACGCGCGTTCCGCCAGGAACGCTCGCCGCGCCGTCTTCGACCGCCGCGCCCCGGCGCAGGCGAAACCGGCGCAAACCTGGTGGTGAAACAGGTGCCAACGACAACAATAAAGGGAGCAATCAGGTGATGAGAAACCAACGTGTGACCAGCGCTACGCTGGGACTGGCCTTGCTGGCTGC

F – ggatccactagttctagaTTTTGCTTTTCCTGCCCGTGTTCCA

R – ctatagggcgaattggagctcGCAGCCAGCAAGGCCAGTCCCAG

**Pant417**

TCTTTGTCCCAGGGGGTGCCTGGGGCGCCAATCATAACAGACGGTTGAGGCCAAGCCGACCGTCCCAGGGACGCAAGCCGCCATGCAAGTGAAACACCTGCTGCTGATCGCCATCCTCGCCCTCACCGCAGCCTGCTCCTCGAACAAGGAGACTGTCGACGAGAACCTGAGC

F – ggatccactagttctagaTCTTTGTCCCAGGGGGTGCCTGG

R – ctatagggcgaattggagctcGCTCAGGTTCTCGTCGACAGTCTC

**Pant418**

ACGGTCGTATCTCGCCGGATTTTCCCCGTTGCCTCAATCAGCTGCGGGATGAGGCGTATGACCGACTTTTCTGCAACCCCTGTGTGATCGTGGAGGCCCTGGTGTCGGAAAGAGTTGTGGTAGTGGGCGCTGGCGTCATCGGCCTGTTGACCGCCCGGGAGCTGGCGCTCGCCGGACTGCGGGTGACCCTGGTGGAGCGGG

F – ggatccactagttctagaACGGTCGTATCTCGCCGGATTTTC

R – ctatagggcgaattggagctcCCCGCTCCACCAGGGTCACCCG

**Pant419**

ATGTCGGGCTGCCGGCATGTCGAAATTGATCCTGTCCAAGACCGCGACCGGCCGGGCCGGTTCATCGCGCGAGTGCGCCCTCGCCGCCGAACGGCGGTCCGCGAGGGCGCGCCGAGTGTAGAATGCGCGCCGGAATAAATCACCCGCAAGAAAGCAGGCGACATCCGTCGCCGATCTTTCCGGCCGCATGCGCAGCCCCT

F – ggatccactagttctagaATGTCGGGCTGCCGGCATGTCGA

R – ctatagggcgaattggagctcAGGGGCTGCGCATGCGGCCGGA

**Pant423**

GAGACTTGACGAACTGTCAAACTCGGACACATGCCGCCGATCCAGCCGCACAGGCGGGGACGGACGGCACCGCCGATCGAATTCCGCCACTTCCCCGCCGGCATGGCTTGGCTATAGTGGTATCCAGCAGCGCGCACCAAGGGATCGCGACATGAGTGACCAGCACGACGAACGTCGACGTTTCCACCGGATCGCCTTCGACGCCGACAGCGAGAT

F – ggatccactagttctagaGAGACTTGACGAACTGTCAAACTCG

R – ctatagggcgaattggagctcATCTCGCTGTCGGCGTCGAAGGC

**Pant432**

ATCGGAGAAAAGCGCATCGCCGATTCCAAGTGCTCCGGCGACAGATGTGCATACCGCATCATCATCGAGGAATGCCCGAGGATCCGCTGTAGGGCCACGCCATGCGCAGCGCCCCGTGTCAGGCATTTCATCTTGCTTTGGATGCCAGGCATGCCTCATTGAAACAGTTCGAATCATTTGATAATCATTATCGATTTGTTTAGCTTTGCCGCCCATCAAAACAACAGCAACTCGGAAATCTCCTCATGCCCCGCTCCATCCCTTTGCGCCCGGCTCCCCTGGCGCTTTCCCTGTC

F – ggatccactagttctagaATCGGAGAAAAGCGCATCGCCGAT

R – ctatagggcgaattggagctcGACAGGGAAAGCGCCAGGGGAGC

**Pant433**

ATTCCGATTCTCGCCTGCATACCGCAGGCAGGTCCCTTCATGCGGTAACCCTTCGCGGGAGTGCGAAGTCAGGTCGACAGCGGCCGGTGGCAGGATGCGCAGACTGGAGCCCCCCATGTGCGACACCCTGATCCTTCGCCACCAGGGCCAGACCTGGTTCGCCAAGAACAGTGACCGCGAGCCCGCCGAACCGCAGCGTCTGCTGCGCCTGC

F – ggatccactagttctagaATTCCGATTCTCGCCTGCATACCG

R – ctatagggcgaattggagctcGCAGGCGCAGCAGACGCTGCGG

**Pant434**

TATCATTGCCTGCCTGAACGCCGGGGGCGAAACCGTCCCCGCGCATCGCCTAATTGCCGGTGCCCGAGATTTGATCGAAGAACAGCGTCGCCGCGCCTTCCTGGGCGCGATGCAGATAACCAGTTGGCTGCCCCGCCAGCCGTTGCCGTTCGCCGCACCCTCGCGGCCGGAGTTGCTGGAGACCCCGCCCCGGGAAGAGCCGGCGGTGCCTGTGCGTGCGCGCGCCGCGATCGATGCGCCGCAGGCCCGCGTCCCGCAAGCCGAGGCGCCGGCCAGCGCGCCGAGCGTGGCTCCCGCCGC

F – ggatccactagttctagaTATCATTGCCTGCCTGAACGCCGG

R – ctatagggcgaattggagctcGCGGCGGGAGCCACGCTCGGCG

**Pant439**

AGACTATGCGGCCATCCGCATGCCTTGCCAGATTCACCGCAGCCTTCCCGGCCCTCTCCCGCTTCGCGGGAACAGGATGTACGGCCCCTGTTCGTTTCTGTCAGGATGCATGGCGCAGTCGGTTCCGGCGCCCGGGACGCCCTGTGCGTCGCGGGGCCAAACATTCAACGGGTACAGAACACGCCTCGCCCGAGAGGTCCTGATAATGCCGCAGCATTACCCGACGTCCGTCCTGTTTGAGCAACGCAGGCGTGCAATCACCCACATCGTGCT

F – ggatccactagttctagaAGACTATGCGGCCATCCGCATGCCC

R – ctatagggcgaattggagctcAGCACGATGTGGGTGATTGCACGC

**Pant440**

TGCCAGTTTTCCTCTAAAATAAAATCCTTTAAAAACAATAAGTTAAAAAACAGACTGGAAGCGAGACGGTTGCAGAATGCCGAATTGGGGATGACAGGGCTTGCAAAATGCAAGGATTCGCCCCCTTCGCCGAGAAGCGTGTCGCGGAAACGAAAACAGGGCCCGAGGGCCCTGTTTTCATCGATCACGACTCGACTTACACGCCGGAAGCTTCGGCGGCAGCCACGTCCTTGATCGACAGCTTGATGCGGCCACGGTTGTCCACGTC

F – ggatccactagttctagaTGCCAGTTTTCCTCTAAAATAAAATCC

R – ctatagggcgaattggagctcGACGTGGACAACCGTGGCCGCAT

**Pant441**

ACTCTTTTCCTGGCCGCTGGCCAAGGTTTTGTAAGAAAAGTCTTCAAGTGGCGTCAGGCTGCGCTGAGCGTTTTTTCTGCCGAACCCTGGCCCCGGTCGACGGGCGCAGCGCGCCGCCGTGGCGGATGCCGCCGTCCCGCCGGCTATGCTTGGGGAGTCGCCCGCTCGACAGCAGAAGTGAGAGTTCTATGAGCACCCATATCCAGCGTTCGGCGCTGTTGCCTTATCCCGCCCGCGCGCTGTTCGATTTGGTGAACGACGTGAAGCGCTATCCGGAGTTCCTGC

F – ggatccactagttctagaACTCTTTTCCTGGCCGCTGGCCAA

R – ctatagggcgaattggagctcGCAGGAACTCCGGATAGCGCTTCA

**Pant442**

TCGCTTTTGTAAATTGGTCTTACCAATTATGCGAGCTTTGCCAGGTTCGGCTGCCCAGCGCCCTTGCACACACAGGTCTTGGCGCGTATCCGATCTGGTCACAGGCAAACCCAACAACAATTAGGGAGCCAACCCCCATGCAAGCCTGGCAACAACTCTATACCCCCCTGGGCAGTCTCGGCCTGTCGGCCGCCGCGGCCGTCATCCCGAT

F – ggatccactagttctagaTCGCTTTTGTAAATTGGTCTTACCAA

R – ctatagggcgaattggagctcATCGGGATGACGGCCGCGGCGG

**Pant443**

TTTTGCATCGAGCGCAGTGGAGCGGGACACGGCGGAAATGGCCAAGGGAGCCGGGCCTACGATCGTAGGCGAGCCTCCCCGGGCCATTTCCTGCGCAGTATCGCCGACGCGCGGCCGATCAGGGCGCGGTCAGTGCACCAGCATGCCGGTGAATAGGTATGCCTGGGCCAGGGTGATCAGTCCGACCAGCGCGGCGAAGAT

F – ggatccactagttctagaTTTTGCATCGAGCGCAGTGGAGCG

R – ctatagggcgaattggagctcATCTTCGCCGCGCTGGTCGGACT

**Pant445**

TCCAATAGGGCGAGCGGCCTTGTCGCTCGACGATGTAAGCAACTCCGTGACTTGGGCGGTGCCGTGCGAACGGGACCGCCTTTTTTTTTGCTGGCGCCCTACGGTTCGGGTTCGTCGGCGGCGTTGGAGACAGCGAATTCGACGCTTGTACGGAGAATCCCCTCTCTCTTCAGGGAGAGAGTTGGGGTG

F – ggatccactagttctagaTCCAATAGGGCGAGCGGCCTTGTC

R – ctatagggcgaattggagctcCACCCCAACTCTCTCCCTGAAGAG

**Pant447**

ACAACGACAAGAACGAGCCGTACCGTCCATGCAGGCCATCGCCCCGAACGCGCTCGACCGCTGGCACGAACACCTGGCGCTGGCGATCCGGCGCCTGCGCCAACCCGACTTCCTCGCCGCGCTGGTCGACGCCATCCACCAACTGGTGCCGATCGAGTCGAGCATGATCAGCCTGGAACGCAAGGGCCACGCGCCCACCCTGCTCTACGAGCG

F – ggatccactagttctagaACAACGACAAGAACGAGCCGTACC

R – ctatagggcgaattggagctcCGCTCGTAGAGCAGGGTGGGCGC

**Pant449**

GCCCGCAGAGCCGGGCACTTTCTTTCCCCCTACGAAGAGAGCCATGGACAGTATTCCCAGTAGTCGTCGCCCGGCCCAGCAGATGCCGGCTGGTGCGATGACCCAAACCAACGCACGACGGAACCCGCAACGGGTCGGTCGGTTGAGCGGCATCCTGGGCTGACCCACTGCTTCTCACCCGTGTCCGGCACGCCGGGTACAGGTGAGTCATGAACTTCGAGAATTCGGTATCCCCCGGCGTATCCGCCTTCGTGGCGAAGATGCGCGTGCGCGCCGCGCAGTCCCC

F – ggatccactagttctagaGCCCGCAGAGCCGGGCACTTTCT

R – ctatagggcgaattggagctcGGGGACTGCGCGGCGCGCACGC

**Pant452**

CTGTGCACCGCGACGTGGCTTCCGGCCGCCGCGTGGCGTCGGAGTCTCGCCGAACGCAGGACGGCCTGCGCCCGCGATCGGCCAGCAGCGTCGATCCTTCCCGAATCTAACAATGCTCGGCGGATGCCGGCCGTGGGGCCCGTCCGTCCAGGAACCGTCATGTTCAGCCCGCTCGTCACCTTTCCCGCGCTCTACACCGCCACCCTGCTCATGCTGGCCGGCTCCGGCCTGTTCACCACCTACATCGGCCTGCGC

F – ggatccactagttctagaCTGTGCACCGCGACGTGGCTTCC

R – ctatagggcgaattggagctcGCGCAGGCCGATGTAGGTGGTGA

**Pant453**

AATGAAAGTGCAGGACAGAGCGGTTTTTAAAATTAATATGAACATGAACTTCCCGGAATTCGGTACTTGGCGGAAAAAGTCGATTCGAATACGACATGAACATATCCGTTAGCGACGCCACGTTCGGCGCAAAACCCTGTGACGGATTCGTCATAAGCCAGCGAATGCTCTATCGCGAGACTTGCCGGCAGTAATAAGCGTCGCCCGGCACTCCGGCAAGCGAGCTTTCCTCCCTACGTCATTCCGTTT

F – ggatccactagttctagaAATGAAAGTGCAGGACAGAGCGGTT

R – ctatagggcgaattggagctcAAACGGAATGACGTAGGGAGGAAAG

**Pant454**

GTAAACGCCGGGCAGATCCCGCTCGATGCCCCGCCACGTCCGGTTCGGGTTTGACCTGAATCAGTGGAACTCGGTGCCCGATCGGGCAGTCTGCTCTTTCAGGATTCATCGCCCAACCTGCCTAGGAGGCTGCTCCATGCTACGTAAACTCGCTGCGGTATCCCTGCTGTCCCTGCTCAGTGCGCCACTGCTGGCTGCCGAG

F – ggatccactagttctagaGTAAACGCCGGGCAGATCCCGCT

R – ctatagggcgaattggagctcCTCGGCAGCCAGCAGTGGCGCAC

**Pant457**

AGCCGTGGCCGGGAAACACGCCCGTGCGGCGAGCGCCACGAAGTTTTCCCGAGCTGTCCTGAAAGTGGCTGCGCCCTGCGCCGGGCCGCGATAGGATGGCGCGAAACCCACATACCCGAGTGAAGTGAGTCGAGGATGTCCGAAACCTTCAAACCCGCCCCCGGTGACGTCGAACTCGTGCA

F – ggatccactagttctagaAGCCGTGGCCGGGAAACACGCCC

R – ctatagggcgaattggagctcTGCACGAGTTCGACGTCACCGGG

**Pant459**

TCGTTCAACAAGACCACAATCCACCCATTCGGGTGTATGTAACGGCCCTGCGGCAGGCGCAAAGCCACACGCCATGCGATCCGCCGAAAAGCGGCGGAGGCCCGCGCGAGCCGGCTGTTACAACTTCGAACAAACCAACCAGGCGAAAACGAAAACGCCCGCCGGATGCGGCGGGCGTCGGGTCCTGCGAATGGCCGTTCAGCCGGCCCGTCCCAGGGCGGGGTCGCCCAGTCCCTGCGGCTGCCGGCGACGAACCACGTAATAGGCCAGGTAGCAGGCGGCGAT

F – ggatccactagttctagaTCGTTCAACAAGACCACAATCCACC

R – ctatagggcgaattggagctcATCGCCGCCTGCTACCTGGCCTAT

**Pant463**

GCAGCCCCTTGTCCGGGTTCCCTTCCCTGACGTGCGTCCGCCCCGCCCTGAAACGTGCGGGTGGGCTGGTAGTCGAGCAGTAGCCGGCCGGACACCTAGTCCCCGGTTTCGTTGCGGTCCCCTGTTTCGCCTCGAACAATGGAGCGTTGCCGATGAGTCAGAAGAACAATAACGAGCTTCCCAAGCAAGCCGCGGAAAACACGCTGAACCTGAATCCGGTGATCGGCA

F – ggatccactagttctagaGCAGCCCCTTGTCCGGGTTCCCT

R – ctatagggcgaattggagctcTGCCGATCACCGGATTCAGGTTCA

**Pant465**

ATTCTTTTTGTCAGATCGCGCCAAATCTGGCTATCTATCGGGTTTGGCAGCGAATGTGGCGAAACCATGTTCGACCGGCACTCCGGTGCCGTCGGAGACCCCTGAACGGTCAGGGCGTCTTCAGTAATTCATGTTCACGGACGAGGGGGCCTCGTGATTTTCCGTTCCGTTTCAAACACCGATTTCCGCGCCCGCGTGCGCACCCTGCTGCTTGCCGGCTCGACCGCGCTGGCATTCGTCGCC

F – ggatccactagttctagaATTCTTTTTGTCAGATCGCGCCAAAT

R – ctatagggcgaattggagctcGGCGACGAATGCCAGCGCGGTCG

**Pant467**

ACATGGAGCACCATAACGGTGCACCCGAAGGCCGCCCAGCTACCCTGAAGAAGACCTGGCCCCGCCAAACATGGGGCCCTGCCCGATTCCGGGCACTGGCACGCAACTTGCTCACTTGTGAGGCAGGTTGCCCTGGCGGAGTATTCCGGCCGGCATCACCCGATACTGGGGCACAGAAAAAGCTCCCAGCTCTAATCCCT

F – ggatccactagttctagaACATGGAGCACCATAACGGTGCAC

R – ctatagggcgaattggagctcGAGGGATTAGAGCTGGGAGCTTTTT

**Pant468**

TGCAGGGGCGCTGGCAGTGGTCGGAGGATGGCGAGGGCTGGCGCCTCGTCGTCGAACTGCAGGAGCGCGACGGCCGGGAATAAAAGGGGGAATTCTCAGCCTGCCTGTACCGAGGTCCCCAAACGGGAGGGCGGAGGCGCGGATCGCTGCGCTACCGCCGGGCATAAAAGAGGGGAGTTCTCAGCCTGCCTGTACCGAGGTCCCCGAAACGGGTGTGGCGGTTGCGCGCGGATGCTGCGTCTGCCGCCGGGTGTAAAAGAGGGGATTC

F – ggatccactagttctagaTGCAGGGGCGCTGGCAGTGGTCG

R – ctatagggcgaattggagctcGAATCCCCTCTTTTACACCCGGCG

**Pant470**

CCTTTTCTGTGAAGCTGGCCAGTTCCGGCCCGGGCACGGTTCGGGCGGGACAGAGGGAGCGAGGATAGCGGCATTCGGTTGCGGATCGGCGTCATCGGTTTAATCAATCAGCCTCTTCCTACGTCTTGAACGACAATGTTCCCACTAAATTTGGTCAGTTTCTGAACCCCGCGGAAATAGAGCCGGACATATGACTGCCACGCCCAAACCCCTGGTCCTGATCATCCTGGACGGCTTCGGCCACAGCGAAAGCCCTGACTACAACGCCATCTACGCCGCGA

F – ggatccactagttctagaCCTTTTCTGTGAAGCTGGCCAGTTC

R – ctatagggcgaattggagctcTCGCGGCGTAGATGGCGTTGTAGT

**Pant474**

GAGCGTTCCAACAAGCAGAAGACCAGGCGCTTTTCCGATTCACGCAGCATCTGCCGCTGCGCTGCCTAGACTTCCCTTGTTCGGCCACGCCGAGAGGACACCCGTCCTCTTCATAAAAGACAGACAAGAACAAAGAGGAAGTCTCATGCTCAAGCACACCGCGAAGGCGCTCGTCTGCGCACTTTCCCTGACCGTCGCCG

F – ggatccactagttctagaGAGCGTTCCAACAAGCAGAAGACC

R – ctatagggcgaattggagctcCCGGCGACGGTCAGGGAAAGTGC

**Pant477**

CTCATCGTTTCAATCGGGAGGGAAATACGGTCGTTCGCACAAAGGCCGGACCATCCGGCCCGGCCTTTGCGTCGTAGGGAGCGGCGGGTTCAGTCGTCGTAGTAGCGACGATGATGCTTGTGGCGCTTCCACTTCTTGTACTGGCCATAGTGATGGCCGTTGTCATGCCGGTAATA

F – ggatccactagttctagaCTCATCGTTTCAATCGGGAGGGAAA

R – ctatagggcgaattggagctcTATTACCGGCATGACAACGGCCATC

**Pant480**

GAAAACGGGCCGTGTAGACAACGCCCCGCCGCAAAAGCGCCGCATTATACCTGCCAAGTTTCGCCTTGGAGGGCGGCTGGTCGGGGAAAAATCTGCTAACAATGGACAGTCCGGCCCAGCCGCTACCTGGGCGATAATGCCCGCCTGACTCATTCTGGGAGTTTCTATGTCGAACCCTGCCGCCCTGGCCGAAGGTGAA

F – ggatccactagttctagaGAAAACGGGCCGTGTAGACAACGC

R – ctatagggcgaattggagctcTTTCACCTTCGGCCAGGGCGGCA

**Pant481**

CGGCCCCAGGCCCCGTACCATGGTCGAATCCCGCGGCCGGGGGGCCGCCGAGGGTTTAAACTCGCGCGTGATCCATCCGTCTCGTTAACCAGAAAGTGAGGAGAAGCCTGTCGATGTTGCTGCGACGACGATAACGGCGCCCTTCGGGCCGTCCCGGCCGCTCACGCTTTGCTCTCGCTCACGAAACGACAGGCTTTCCCCACCATGCCCGACTACGTCAACTGGTTACGTCACGCTTCGCCCTACATCAACTCGCACCGGGACCGCACCTTCGTGGTCATGCTCCCCGGCGA

F – ggatccactagttctagaCGGCCCCAGGCCCCGTACCATG

R – ctatagggcgaattggagctcTCGCCGGGGAGCATGACCACGAA

**Pant482**

GAAATGTTACAGGTTGAGTGCATTGGTCGGAAATAAGAAAGCCCCGGTCGGAAACCGGGGCCTTCATAGGGGAAAAACATCTGACAGCGTGGGCCTCGGCCGGTGTATCGCTCCGGCCCGCCATGCCTTTCTCCGCACGGGCGGGAGGCAATACTCGCCGCCTTCGTGGCTCAGCGAACCTCCACCACCACCTTGCCGACCGCCCTGCGCTGGCCGAGGGCGTCGATAGCGTCGCCGGCCCGTTCCAGCGGGAAGCGCTGCGA

F – ggatccactagttctagaGAAATGTTACAGGTTGAGTGCATTGG

R – ctatagggcgaattggagctcTCGCAGCGCTTCCCGCTGGAACG

**Pant483**

GAACGCAGGATCCGGACCGGACGGGCCTGGCCGGGGTCGGTATCGCGCAAGCATAGCGAGCCGCCGCCCCCGGCGGCCAGCCGGAGCGGAGGGTACAAGG AGTTGGAGTGTACGAAGGGGCCGGCTGTTCAGTCGCGCCGGCGCGGGTTCACTCGGATTTGTCGGCGTTGCCGCCCGCGCCGTTGCCGC

F – ggatccactagttctagaGAACGCAGGATCCGGACCGGACG

R – ctatagggcgaattggagctcGCGGCAACGGCGCGGGCGGCAA

**Pant486**

CCTTGTGATCGGATTGCCCGCCGAAGGCGGAAGACGAAACAGCCTAACACATCGTGATAGCTAATTTGATTCGAAAAGCCTTAATCGATAGCTTTGCTGAATTATTACGCTTCACGTGGAGGTCATTGCCCGATACTGCAGGGCTTCCGCCAGGTGCCGGCGTTCGATGGCATCGATGCGCTCCAGGTCGGCCAGCGTCCG

F – ggatccactagttctagaCCTTGTGATCGGATTGCCCGCCG

R – ctatagggcgaattggagctcCGGACGCTGGCCGACCTGGAGC

**Pant488**

TTAACATGCCCATCCGCAGCGGTTGGGCGCAGTCATGCAAGGCCGTTGGCGACCTTGAATCGCTACCCTAGACCATGCGGCGGGCAGCGACAACGAGGCAAGGCGACGAACGGCCGCGGTGCGAAGCCCGCAGCGAGGCGGGCCGCGGCTGCGCCATGGGCCTTCGCCCTTGCGGCGCGGGGCGCCTTTCTGATATAAAGCTGCGCTCTTTTCTGGGGCCACGCTCTCCTTCGTCGGGATGCAGCGGGTAAGACCCCGGTGAAA

F – ggatccactagttctagaTTAACATGCCCATCCGCAGCGGTT

R – ctatagggcgaattggagctcGTTTCACCGGGGTCTTACCCGCTG

**Pant494**

ATGAACGACATGGCCAACTCCCCGATGAACTCCTCCCTTGCTCCCGAGCGGATTGACTTCAATACGCCAGCCCTGCAACGCAAGCGCCGCATCCGTGCCCTCAAGGACCGCCTGACCCGCTGGTACGTGCTGGTCGGCGGCCTGGCCGTGCTGGCGGCGATCACCCTGATCTTCTTCTACCTGGCCTACGTCGTGCTCCCGCTGTTCCGCGGCGCCGACCTGGAGACCCGCGAAGCGCTGACTCCGGCCTGGATGCAGCAGGCGCAG

F – ggatccactagttctagaATGAACGACATGGCCAACTCCCCG

R – ctatagggcgaattggagctcCTGCGCCTGCTGCATCCAGGCCG

**Pant499**

AACGGCTCGCCGCCCGTCGTTACCTTTGTCGCCACGGCCGCTGCCCCGGCTTCTCGCCGGGGGCCGCCCTGGTGCTACCGAAATGCCCAGGCGCGCGGCGCGCCGCCAGCCGTCGCCCATGCTGTCGTTGTGCCAGGTCCGAGTGGAAATACCGGGCTAAAGCCTTCCGGCTTTTTTGATTGAACGCCCAATCAATATAGGCATGGCATTTGCCTTGC

F – ggatccactagttctagaAACGGCTCGCCGCCCGTCGTTAC

R – ctatagggcgaattggagctcGCAAGGCAAATGCCATGCCTATATT

**Pant501**

TTTCCTCCGCGACCTGCGCCGGCGCCTGCCCCGCGAATACCGCTTGAGCATCACCGGGTTGCTCGACTGGGGCAGCAACGCCGAGCCGGCGGCGATCAACCGGCTCAAGGGCGTGGTCGATGAAGTGGTGGTGCAGACCTACCAGGGGCGCCACAGCATTCCCAACCACGCCGCCTACCTCCCGCGGGTCAGCCGCCTGCAGCTGCCGTACCGGATCGGCCTGGTCCAGGGAGGCGACTGGCAAGCCCCCGACTACCTGGAAAAGAGCCCCTGGTT

F – ggatccactagttctagaTTTCCTCCGCGACCTGCGCCGGC

R – ctatagggcgaattggagctcAACCAGGGGCTCTTTTCCAGGTAG

**Pant502**

TTGCCGTCCGTCGAGGCCGCGGTGCGCGCCGAGGACTACGGCGCCTTCTGGCTGTGGAGCGGCGTCGCGCCGCAGCCGGTGCTGGCGCGGGCCGAGACGCTGTACATCCTGCAGGGACAGATCAGCCAGTCGCGCCGGCGCCCCGGCGAGGGCGTGCGGCTGATCGCCCAGGGCATCTCGGTGCCGCGCCTGGCGCGGGGTGAGGTCTGGGTGGTGTACCGCGCCCATACCCTGCGCTGGACGCCGCAGATCTACCAGGCCGTGCTGAACCAGGTGCAGCG

F – ggatccactagttctagaTTGCCGTCCGTCGAGGCCGCGG

R – ctatagggcgaattggagctcCGCTGCACCTGGTTCAGCACGGC

**Pant503**

ACGTTCGTGTTACAACTCGCCTGAACGCTGCCTTTCCATCGTGGCCGTTGGCGCCGTTGTCCGGGCTCCGCGGCGCAACGCACGCGGAACCTGGCGCGAGGTTGGCCATTCGTCGTAGTGCCAGGGGGGCCACGCCATGCTGAAACTGCTCGACCTGCTCTCTGCCGTCGCCCTGCTGGTGTGGGGCACGCATATCGTCCGCACCGGAATCCTCCGGGTCTACGGCGCCGACCTGC

F – ggatccactagttctagaACGTTCGTGTTACAACTCGCCTGAA

R – ctatagggcgaattggagctcGCAGGTCGGCGCCGTAGACCCG

**Pant509**

GACCCGCGAGCCAGACGCGGCGGGCGAAAAAACGGCGGCCGGGGAGCAATAGCCCGAAACAAAATCCGGCTGGTCCTGTCGCCCGTACCGCCGCAACATGGCGCGCGTCGATTCCCCCAAGAGAGAAAGACTACAATGCAGCGTGTTCTCAGCATCGCCCTCGCCTTCTGCCTGGCGCTGACCGTCAGCCTGGATGCCAATGCCAAGCGCTTCGGCGGCG

F – ggatccactagttctagaGACCCGCGAGCCAGACGCGGCG

R – ctatagggcgaattggagctcCGCCGCCGAAGCGCTTGGCATTG

**Pant511**

TTCTCTTTGGCTGAATGAGCGGACGAACGCCAAGGGGGCGCCGGCTCGTGGAGCCGGCGCCAGGGTCAGGGATGAACCGCTCGGGCTATGGAGGGCATGGCTGTTTGGTTGATGCAGGAACGGCGGGGAGAAAAGAAGAGAACGGAGGGAGATTCTGGGGGCCAGTGCACGATTTCGAAGAAGGCTT

F – ggatccactagttctagaTTCTCTTTGGCTGAATGAGCGGACG

R – ctatagggcgaattggagctcAAGCCTTCTTCGAAATCGTGCACTG

**Pant512**

AGCGGCTTTTCTGTTGATAAGTACGTCGAGCCCAGTAAAGACATAGCCTGTAGCGAATGATAAGGCTGTCCCAAACCCTATCTGGGCCCTGTTGATAAGCGTTTTTTAGCTGTGGATGGAATGGCTCGTTATCAACATGTGGAGTTATGCACAGAGCTTCGGCGGGTTTATCTACCCTCCTGTCCCTGGGGTTTTCCACAGGTTTCAGGCGAGCGTATCCGGGCCGTGGCAGGACCGCAGCCAGGTAGGAAGAAGGAGATG

F – ggatccactagttctagaAGCGGCTTTTCTGTTGATAAGTACGT

R – ctatagggcgaattggagctcCATCTCCTTCTTCCTACCTGGCTG

**Pant513**

AGCAGATCGCGGCTACGGGATTGCCAGTGATGGAGCCGAGTGGAGGAAACCGCACTCGGGCGACCATTGCGTCGCGGGCCATGCTGCGCGATAGCCTCGTACGCTAGGGAAGTACAACTTGGCTGGGAACGACGGTGCCGGGTCATCGAAGGGGGGATGACACTACAGGGATCGGGATGCGCGGGCATCGACCTATGCATGAG

F – ggatccactagttctagaAGCAGATCGCGGCTACGGGATTGC

R – ctatagggcgaattggagctcCTCATGCATAGGTCGATGCCCGCG
